# Temporal Metabolomics Reveals Additional Diterpene Resin Acid Metabolites in *Pseudomonas abietaniphila*

**DOI:** 10.1021/acsomega.6c00600

**Published:** 2026-03-28

**Authors:** Kristina Kshatriya, Christian Paetz, Jonathan Gershenzon, Axel Schmidt

**Affiliations:** † Department of Biochemistry, 28298 Max Planck Institute for Chemical Ecology, 07745 Jena, Germany; ‡ NMR/Biosynthesis Group, Max Planck Institute for Chemical Ecology, 07745 Jena, Germany

## Abstract

Diterpene resin acids are abundant oleoresin compounds in conifer trees and persistent pollutants in pulp mill effluents. The bacterium *Pseudomonas abietaniphila* BKME-9 has previously been reported to metabolize abietane-type diterpene resin acids, but the complete catabolic pathway and the identities of downstream metabolites are not yet fully elucidated. In this study, time-resolved metabolomics was used to characterize the metabolism of dehydroabietic acid (**1**) by *P. abietaniphila*. In addition to the known intermediates 7β-hydroxy-dehydroabietic acid (**2**), 7-oxo-dehydroabietic acid (**3**), and 11,12-dihydroxy-7-oxoabieta-8,13-dien-18-oic acid (**4**), four previously undescribed diterpenoid acids were detected at later incubation times. These new metabolites were identified as 5-hydroxy-dehydroabietic acid (**5**), 5,7β-dihydroxyabietan-18-oic acid (**6**), 5-hydroxy-pimara-8-en-18-oic acid (**7**), and 5-hydroxy-7-oxo-pimara-8-en-18-oic acid (**8**) by spectroscopic analysis. Time-course data revealed sequential formation and accumulation of these compounds through new oxidations and rearrangements of dehydroabietic acid. These findings expand the known diversity of microbial diterpene metabolites and provide insights into the metabolic network of *P. abietaniphila* involved in transforming plant-derived diterpenoids.

## Graphical abstract

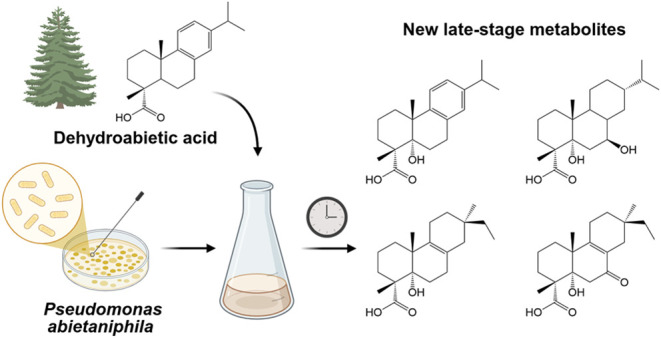

## Introduction

Conifer trees synthesize large quantities of diterpene resin acids (DRAs) as part of their oleoresin-based defensive strategy.^1–3^ DRAs constitute the major nonvolatile fraction of oleoresin and are highly abundant in conifer tissues, such as needles, bark, wood, and roots. These tricyclic diterpenoids are C_20_ carboxylic acids, with structural diversity arising from differences in oxidation state, stereochemistry, and the degree of unsaturation of the fused-ring system, including whether the C ring is aromatic or reduced. Many DRAs possess antimicrobial and insect feeding-deterrent properties that contribute to the chemical defenses of conifer trees. Nevertheless, certain organisms associated with conifers have developed metabolic pathways that allow them to tolerate, transform, or utilize DRAs as carbon sources.

Beyond forest ecosystems, DRAs are also prevalent in industrial settings, particularly in pulp and paper mill effluents, where they can enter aquatic systems as persistent and toxic pollutants.^4^ In these settings, high concentrations of DRAs have been shown to negatively affect aquatic organisms.^5–8^ Currently, most wood-processing facilities that handle material from conifers treat their wastewater to reduce the concentration of DRAs and other toxic byproducts. These treatments often incorporate biological processes that enable microbial bioremediation, such as activated sludge basins and aerated lagoons.^4^

Previous research on microorganisms involved in wastewater treatment has led to the discovery of bacteria capable of degrading DRAs.^9–11^ Among these is *Pseudomonas abietaniphila* BKME-9, a bacterium isolated from a pulp and paper wastewater treatment system.^12^ This strain has been found to catabolize abietane-type DRAs via a diterpene degradation (*dit*) gene cluster.^13^ This gene cluster, first described in *P. abietaniphila*, encodes several enzymes involved in early steps of DRA degradation. Dehydroabietic acid (DHAA; **1**), a major DRA in conifer oleoresin, is converted by early enzymatic steps in the pathway into several intermediates. These include 7β-hydroxy-DHAA (**2**), 7-oxo-DHAA (**3**), and 11,12-dihydroxy-7-oxoabieta-8,13-dien-18-oic acid (**4**), which have been detected in *P. abietaniphila* cultures grown with DHAA, abietic acid, or palustric acid.^13,14^

Still, many aspects of DRA catabolism by *P. abietaniphila* remain unclear. In particular, the identities and structures of downstream metabolites and the timing of their appearance during growth are not well understood. It is also uncertain whether additional catabolic branches or enzyme activities contribute to diterpene degradation beyond those already characterized in the *dit* gene cluster.

In this study, we used a time-course analysis to investigate the degradation of DHAA by *P. abietaniphila* under carbon-limited conditions. By sampling cultures at regular intervals and analyzing extracts via high-resolution electrospray ionization mass spectrometry (HRESIMS), the temporal dynamics of DRA-derived metabolites were monitored. In addition to detecting known intermediates, several previously unreported compounds were identified that accumulated at later time points. Structural elucidation of these metabolites revealed new oxidative and rearrangement reactions, expanding the known diversity of abietane metabolites. These findings provide new insights into the metabolic potential of *P. abietaniphila* and demonstrate the utility of a time-resolved approach for uncovering novel microbial transformations of plant-derived natural products.

## Results and Discussion

### DHAA Metabolism Occurs under Carbon-Limited Conditions

To investigate DHAA metabolism by *P. abietaniphila*, we first sought conditions under which derivatives would be detected. Preliminary tests showed that while the bacterium grew well in a rich medium such as LB, it did not metabolize DHAA (**1**) when the compound was added to the culture (Figure S1). This is consistent with previous reports suggesting that DRA metabolism in this species is repressed in the presence of other carbon sources.^15^

Therefore, a minimal medium comprising M9 minimal salts was used to ensure that DHAA (**1**) was the sole carbon source available to *P. abietaniphila*. However, while DHAA (**1**) metabolism was very efficient under these conditions, no bacterial growth was detected, as measured by OD_600_ (Figure S2). To enable DHAA metabolism while avoiding growth limitations, cultures were initially grown in LB and then transferred to DHAA-supplemented minimal medium for subsequent analyses. These results confirmed that *P. abietaniphila* metabolizes DRAs under carbon-limited conditions. Additionally, no DHAA-derived metabolites were detected in uninoculated controls, indicating that the observed transformations require active bacterial metabolism.

### Known *dit* Pathway Intermediates Accumulate Transiently

Previous studies on *P. abietaniphila* and the *dit* gene cluster have shown that DHAA (**1**) is initially hydroxylated at the C-7 position by the cytochrome P450 monooxygenase DitQ, forming 7β-hydroxy-DHAA (**2**).^16^ This intermediate is then predicted to be oxidized by an unidentified dehydrogenase to produce 7-oxo-DHAA (**3**).^14^ DitA, a ring-hydroxylating dioxygenase, further oxidizes **3** at the C-11 and C-12 positions of the C-ring to generate a nonaromatic dihydrodiol (**4**).^14^ Compound **4** is then predicted to undergo ring cleavage, potentially catalyzed by the putative dioxygenase DitC.^14^ However, the timing and sequence of intermediate formation during DHAA transformation by *P. abietaniphila* remain incompletely characterized. To investigate this, *P. abietaniphila* was cultured in minimal medium supplemented with DHAA (**1**), and metabolites were extracted hourly for analysis (Figure 1).

**Figure 1:**
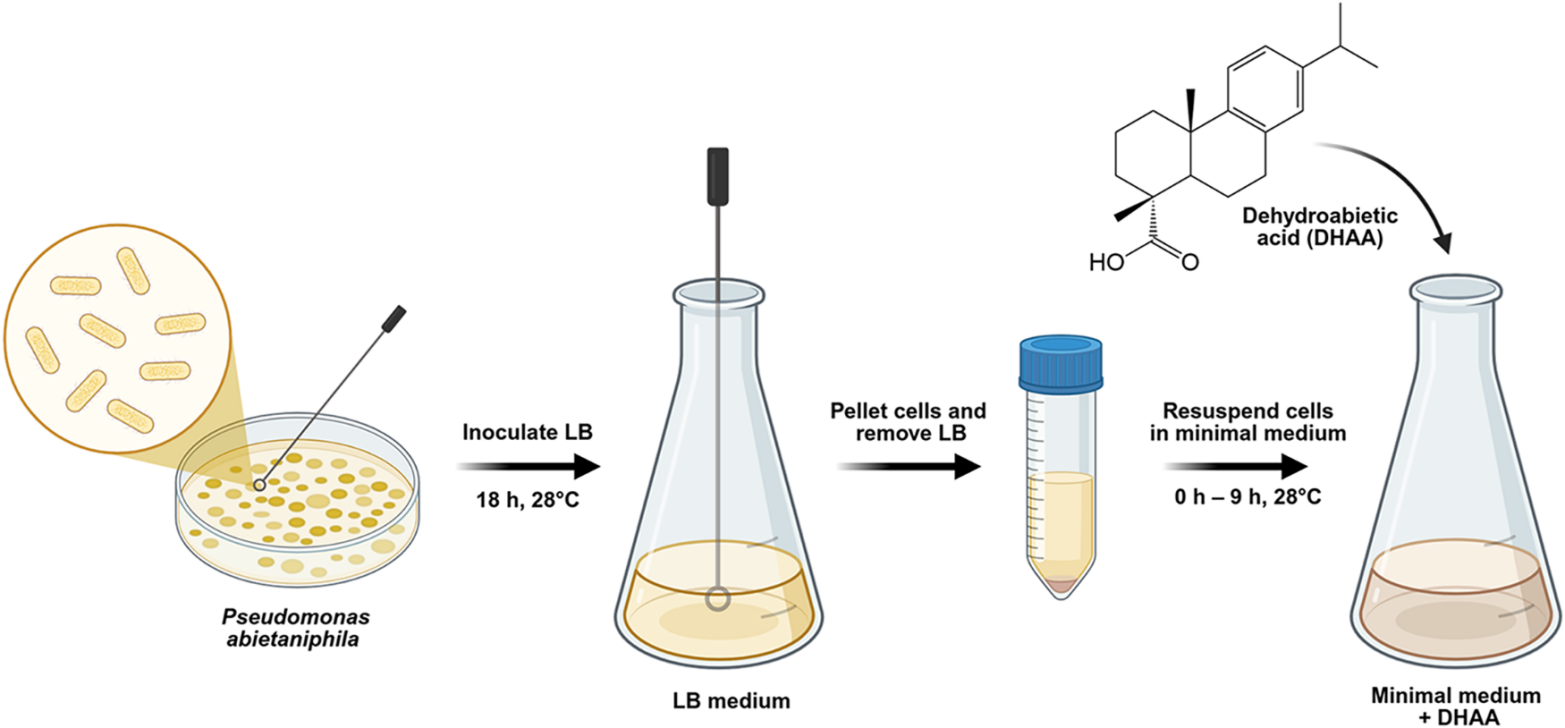
Scheme of the experimental setup. *P. abietaniphila* BKME-9, an environmental bacterium known for its ability to metabolize diterpenes, was inoculated in LB medium for 18 h, pelleted through centrifugation, and resuspended in a minimal medium containing the diterpene resin acid dehydroabietic acid (DHAA; **1**) as a sole carbon source. Samples were collected hourly over the course of 9 h and analyzed by UHPLC–HRESIMS to monitor metabolite production.

First, the presence of DHAA (**1**) after the medium exchange was confirmed by HRESIMS (Figures 2–3). DHAA (**1**) was detectable only at early time points and steadily declined with incubation time (Figure 4). Over 99% of **1** was metabolized within 6 h. Previous studies reported similar levels of metabolism by *P. abietaniphila*, but over longer time frames,^12^ likely due to differences in culture conditions.

**Figure 2:**
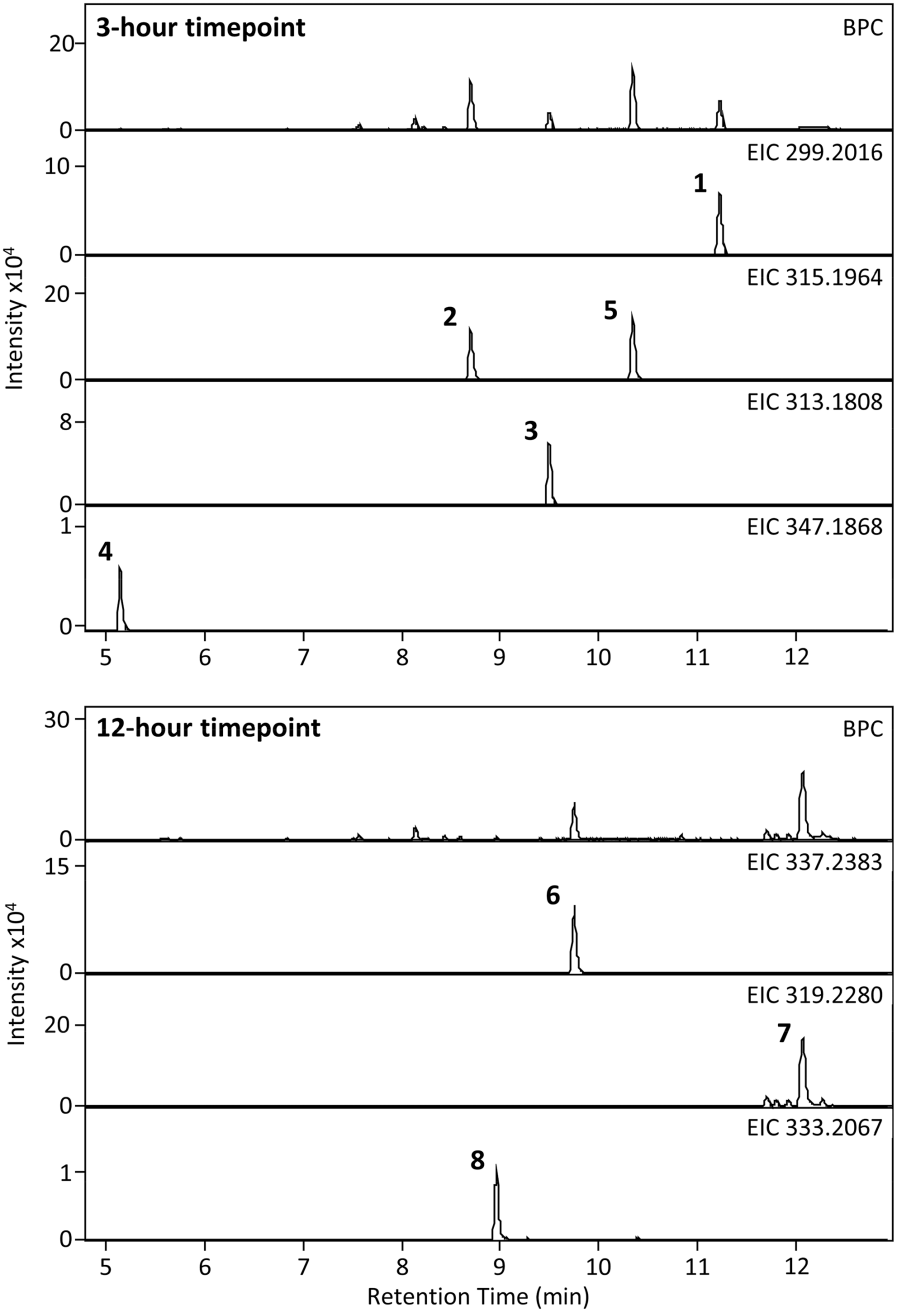
Identification of diterpene resin acid metabolites from *P. abietaniphila*. The base peak chromatogram (BPC) of metabolites from liquid cultures grown in a minimal medium supplemented with DHAA (**1**) is depicted at two time points. The extracted ion chromatograms (EICs) of the seven metabolites (**2**–**8**) that were identified after 3 and 12 h of incubation is shown. Four of the products (**5**–**8**) had not been identified previously.

**Figure 3:**
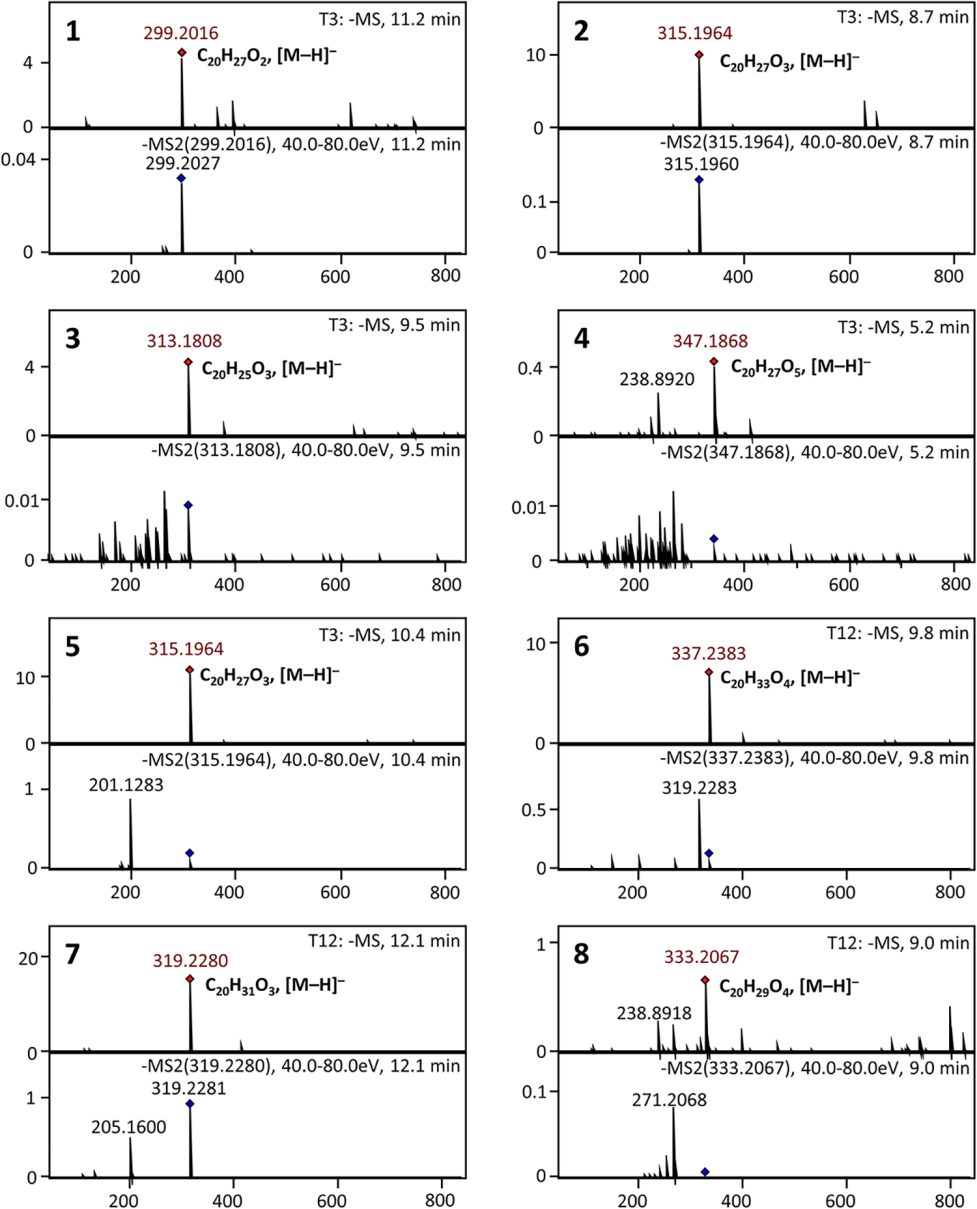
Negative-ion electrospray ionization MS and MS/MS of DHAA (**1**)-derived metabolites found in *P. abietaniphila* liquid cultures grown in minimal medium.

**Figure 4:**
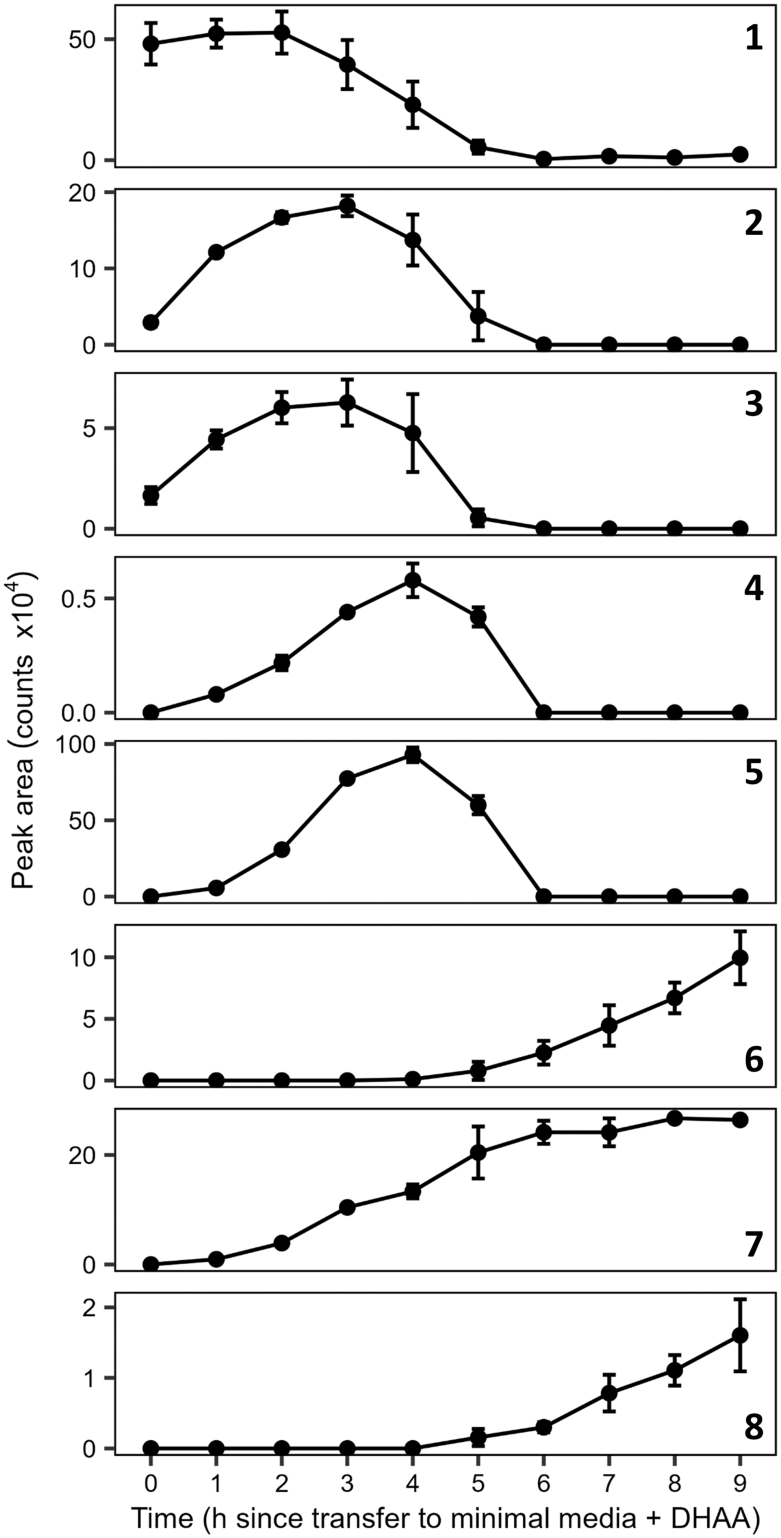
Time-course analysis of *P. abietaniphila* DHAA (**1**) metabolites. Bacterial cultures were harvested every hour over a 9 h period and analyzed by HRESIMS. The seven compounds identified had distinct patterns of abundance over the time course of measurement. Error bars represent the standard deviation among three biological replicates at each time point.

The three previously characterized *dit* pathway intermediates (**2**–**4**) were monitored by HRESIMS analysis (Figures 2–3). All three metabolites were detected at early time points but were no longer observed after 6 h of incubation. Compounds **2** and **3** increased in abundance until 3 h, followed by a steady decline (Figure 4). Compound **4** followed a similar trend but peaked at 4 h. The turnover of these intermediates suggests that once formed, they are processed into downstream products, supporting the idea that further metabolism may occur beyond the known *dit* pathway. The identities of the known DHAA catabolites (**2**–**4**) were confirmed by NMR spectroscopy, since no standards were available. A comparison of NMR data reveals that the DHAA scaffold remains intact in all isolated structures, with structural modifications at C-5 resulting in distinctive chemical shift changes (see Supporting Figure 5 and Table 1). Based on this, we assumed the β-orientation of the methyl groups C-19 and C-20 in all structures and developed our structure elucidation accordingly. We would like to emphasize that our report is based on the relative stereochemistry of the metabolites, as determined by ROESY correlations. Due to the small quantities isolated, particularly for compound **4**, absolute stereochemistry could not be determined, nor was crystallography or derivatization a feasible alternative.

Compound **2** (*m*/*z* 315.1964; C_20_H_27_O_3_, [M–H]^−^), 7β-hydroxy-DHAA,^17^ was isolated as a colorless solid. Its NMR spectra resembled those of DHAA (**1**), but a major difference was the methine signal H-7 (δ_H_ 4.74, *dd* (8.5/8.5)/δ_C_ 71.1) attributed to a hydroxylation. The HMBC spectrum showed correlations of H-7 to C-6 (δ_C_ 33.1), C-8 (δ_C_ 138.8), C-9 (δ_C_ 147.8), and C-14 (δ_C_ 126.0), and a COSY correlation to the methylene H-6α/β (δ_H_ 1.82), which appeared as broad singlet. The hydroxyl group in position 7 was found to be in β-orientation, as H-7α showed correlation with the methine H-5α (δ_H_ 2.20) in the ROESY spectrum (see Supporting Figures 6–35 for details of the structure elucidation and Supporting Table 2 for NMR data).

Compound **3** (*m*/*z* 313.1808; C_20_H_25_O_3_, [M–H]^−^), was confirmed to be 7-oxo-DHAA and its NMR data in CDCl_3_ have been previously reported.^17^ The NMR data in MeOH-*d*_3_ showed the chemical shifts of H-6/C-6 to be at low field (δ_Hβ_ 2.78, *dd* (14.1/17.8 Hz) and δ_Hα_ 2.39, *dd* (3.0/17.8 Hz)/δ_C_ 38.6), indicating an electronegative substituent at C-7. Also, typical for an α,β-unsaturated ketone, the chemical shift of C-9 (δ_C_ 154.9) appeared at low field. Further evidence for the 7-oxo substitution came from the HMBC correlations of H-14 (δ_H_ 7.80, *d* (2.1 Hz)), H-5 (δ_H_ 2.66, *dd* (3.0/14.1 Hz)), and H-6 to C-7 (δ_C_ 200.8). See Supporting Figures 36–62 for details of the structure elucidation and Supporting Table 2 for NMR data.

Compound **4** (*m*/*z* 347.1868; C_20_H_27_O_5_, [M–H]^−^) was only isolated in very small quantities. It was predicted to be 11,12-dihydroxy-7-oxoabieta-8,13-dien-18-oic acid, but this assumption was based solely on the interpretation of ^1^H NMR data. No ^1^H–^13^C correlation data have been published. In accordance with Martin et al.,^14^ we obtained our NMR data in MeOH-*d*_3_. We confirmed the presence of an 11,12-dihydroxylation and the dearomatization of ring C of the DHAA scaffold. However, we observed a signal cancellation, which was probably caused by the proton resonance of H-19 occurring at exactly the same frequency as an aliphatic impurity in our sample. This resulted in the absence of signals for the C-19 methyl group in our HSQC and HMBC data. Consequently, important correlation data are missing and the structure must therefore remain incompletely assigned (see Supporting Figures 63–69 for details of the structure elucidation and Supporting Table 2 for NMR data).

### Identification and Characterization of Previously Undescribed DHAA Derivatives

In addition to the known intermediates, untargeted HRESIMS analysis revealed four previously undescribed DHAA derivatives **5**–**8** (Figures 2–3). To determine compound identities, metabolites **5**–**8** were extracted from large-scale *P. abietaniphila* cultures at time points corresponding to their highest abundance. Compounds were purified using solid-phase extraction followed by HPLC fractionation, and their structures elucidated by NMR spectroscopy (Scheme 1).

**Scheme 1:**
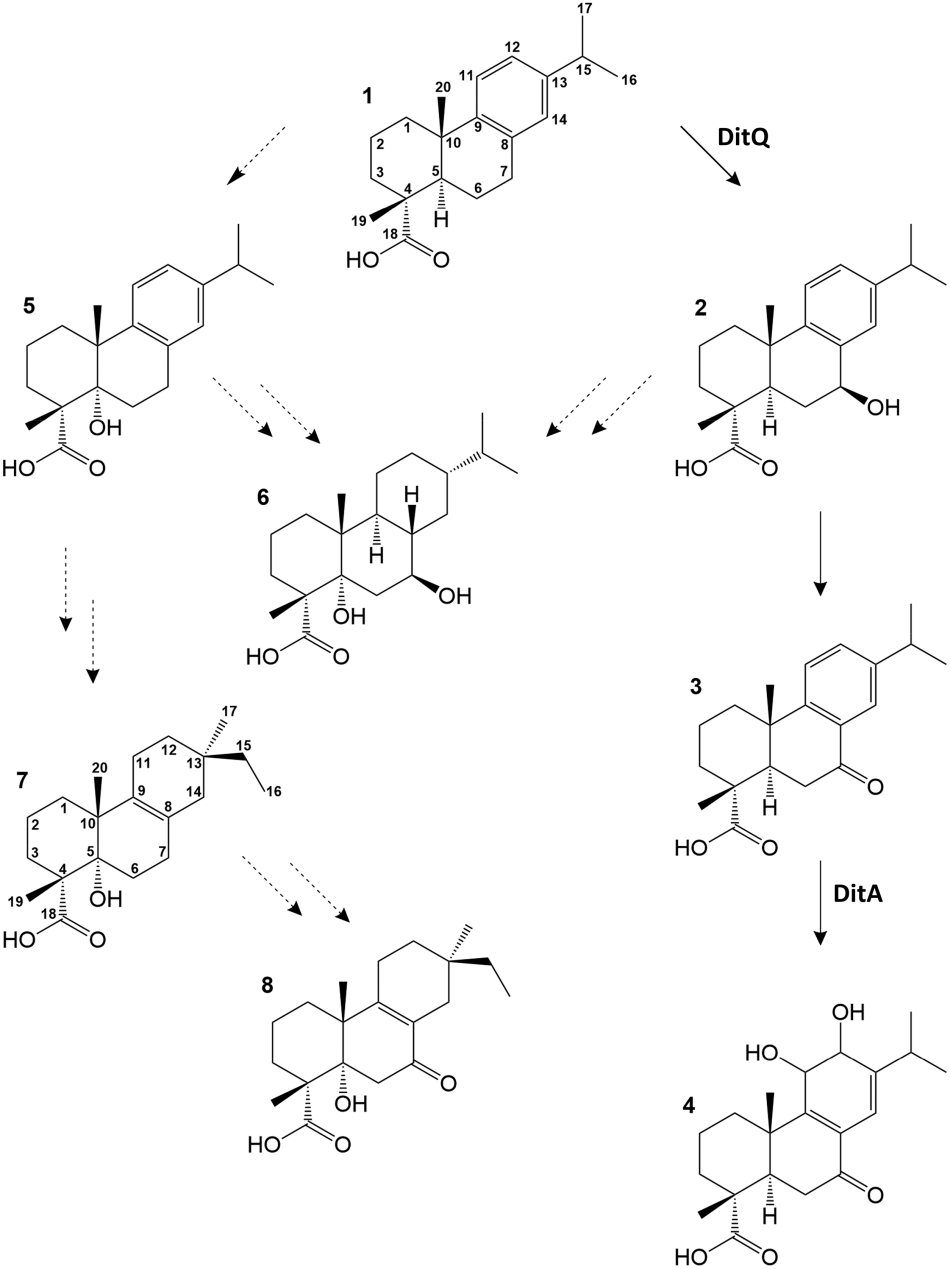
Chemical Structures of *P. abietaniphila* DHAA (**1**)­^a^

a Metabolites depicted in possible order of conversion (dashed arrows represent speculated pathway reactions). Compounds **2**–**4** were previously reported and **5**–**8** are new.

Compound **5** (5-hydroxy-dehydroabietic acid; *m*/*z* 315.1964 [M-H]^−^, calcd for C_20_H_27_O_3_, 315.1960) shared the same molecular formula as **2** but eluted at a different retention time (10.4 min vs 8.7 min), indicating structural differences (Figure 2). As with **2**, compound **5** was detected only at early time points, although it reached peak levels at 4 h (Figure 4). The NMR HMBC data for compound **5** showed correlations from H-19 and H-20 to the hydroxylated quaternary carbon at position 5 (δ_C_ 76.8). This hydroxy group is α-oriented, as revealed by a ROESY correlation between H-19 and H-6β, which can only occur if rings A and B of the molecule are *trans*-fused. Therefore, the molecule was deduced to be 5-hydroxy-dehydroabietic acid (see Supporting Figures 70–94 for details of the structure elucidation). While cytochrome P450 enzymes such as DitQ might be expected to catalyze such a reaction, previous gene knockout studies showed that DitQ specifically modifies C-7,^16^ indicating that a different P450 monooxygenase may be responsible for the formation of **5**.

In the incubation assays, compound **7** (5-hydroxy-pimara-8-en-18-oic acid; *m*/*z* 319.2280 [M–H]^−^, calcd for C_20_H_31_O_3_, 319.2273) showed a linear increase in abundance until 6 h, after which levels plateaued (Figure 4). Similar to compound **5**, the NMR data for **7** show 5α-hydroxylation, as revealed by HMBC correlations between H-19 and H-20 with C-5 (δ_C_ 77.6). Additionally, dearomatization of the former DHAA (**1**) structure is evident, as well as a change in the side chain of ring C. C-13 appears to be quaternary (δ_C_ 32.1) and is substituted with an α-oriented methyl group (position 17, δ_H_ 0.82/δ_C_ 24.0) and a β-oriented ethyl group. The orientation of C-17 was deduced from a ROESY correlation with H-11α (δ_H_ 1.92) and H-12α (δ_H_ 1.39); these correlations would not occur if the substitutions at position 13 were inverted. The low-field shifts of the methylene proton signals of position 7 (δ_Hα_ 1.91, δ_Hβ_ 2.07) and HMBC correlations from H-20 and H-11/12 revealed the presence of a double bond between rings B and C (δ_C‑8_ 126.4, δ_C‑9_ 134.7). See Supporting Figures 121–140 for details of the structure elucidation.

Compounds **8** (5-hydroxy-7-oxo-pimara-8-en-18-oic acid; *m*/*z* 333.2067 [M–H]^−^, calcd for C_20_H_29_O_4_, 333.2066) and **6** (5,7β-dihydroxyabietan-18-oic acid; *m*/*z* 337.2383 [M–H]^−^, calcd for C_20_H_33_O_4_, 337.2379) only began accumulating after 4 h, showing steep increases in abundance at later time points (Figure 4). The NMR data showed that compound **8** shared several structural features with **7**, including the same orientation of the rearranged side chain at C-13, and the α-hydroxyl group at C-5. However, unlike in **7**, C-7 was oxidized to a keto function (δ_C_ 201.1). Consequently, the resonance for C-9 appeared at a lower field (δ_C_ 165.3). Also, the proton resonances of position 6 showed a low-field shift (δ_Hα_ 2.45, δ_Hβ_ 2.93). See Supporting Figures 141–163 for details of the structure elucidation.

The large number of hydrogen atoms in the molecular formula of **6** suggested a highly saturated structure. Indeed, as revealed by the NMR data, **6** contained no double bonds except for the carboxyl function at C-18. The hydroxy function at position C-7 of **6** has β-orientation as revealed by the large coupling constants for the correlations of H-7α (δ_H_ 3.59) with H-6β (δ_H_ 1.79, *J*_HH_ = 10.5 Hz) and H-8β (δ_H_ 1.20, *J*_HH_ = 10.5 Hz). The C-20 methyl group (δ_H_ 1.05, δ_C_ 41.5) shows a strong ROESY correlation with H-8 (δ_H_ 1.20). H-9 (δ_H_ 1.62) shows ROESY correlations with H-7α and H-14α. Based on these data, we determined H-8 to have β- and H-9 to have α-orientation. The isopropyl side chain at position 13 has α-orientation as revealed by the ROESY correlation of H-8β with H-13β (δ_H_ 0.98). See Supporting Figures 95–120 for details of the structure elucidation. The two hydroxylations at C-5 and C-7 suggest that compound **6** may arise from the combined activities of the enzymes responsible for the formation of **2** and **5**.

### Late-Stage Metabolites Accumulate and Persist as Potential End Products

Unlike the other intermediates, compounds **6**–**8** did not decline in abundance at later time points and may therefore represent persistent late-stage metabolites in the pathway. To further investigate this possibility, the time-course experiment was repeated over a longer period. *P. abietaniphila* was incubated for 24 h in minimal medium supplemented with DHAA (**1**), and samples were collected every 3 h. Compounds **6**–**8** remained abundant throughout later time points and persisted until at least 24 h (Figure S3). While no downstream products beyond compound **4** have been previously identified, prior work by Hemingway and Greaves^11^ demonstrated that approximately 80% of the radiolabel from ^14^C-labeled DRAs was released as ^14^CO_2_ following microbial feeding, signifying substantial catabolism. However, the fate of the remaining 20% of the radiolabel was unaccounted for, raising the possibility of alternative metabolic end points, such as **6**–**8**.

### Evidence for a More Complex DHAA Transformation Network

The metabolic pathway leading to compounds **5**–**8** is still uncertain, as most of these metabolites appear to result from multiple structural modifications. Nevertheless, the discovery of these new intermediates and potential end products suggests that *P. abietaniphila* metabolizes DHAA (**1**) through a more complex pathway than previously proposed. Earlier studies hypothesized that compound **4** undergoes C-ring cleavage and is further degraded for use in primary metabolism.^9,14^ This process likely still occurs, although downstream intermediates in this pathway may have escaped detection using the analytical methods applied. However, this does not exclude the possibility of additional branches of DHAA (**1**) degradation in which *P. abietaniphila* introduces alternative modifications to the original carbon skeleton. This hypothesis is supported by previous observations from Smith et al.,^16^ who found that a *dit*Q knockout mutant retained the ability to grow slowly on DHAA (**1**) despite silencing the first gene in the pathway, thus suggesting the presence of at least one alternate metabolic route.

Although the enzymatic steps responsible for producing compounds **5**–**8** are currently unknown, it is possible that the *dit* gene cluster is involved. While all 19 genes in the cluster have been sequenced and annotated with putative functions,^13,16^ most remain functionally uncharacterized. To date, only DitQ (cytochrome P450)^16^ and DitA (ring-hydroxylating dioxygenase)^14^ have been directly linked to specific steps in DRA metabolism in *P. abietaniphila*. Insertional mutations in four additional *dit* genes have resulted in *P. abietaniphila* losing the ability to grow on several DRAs, including DHAA (**1**), abietic acid, and palustric acid, thus implying active roles in DRA degradation.^13^ These four genes include *dit*I (putative dehydrogenase), *dit*H (putative isomerase), *dit*F (putative sterol carrier-like protein), and *dit*C (ring-cleavage dioxygenase). Nevertheless, it is also possible that enzymes responsible for the newly identified metabolites might be encoded by genes outside the *dit* cluster.

What is clear from both the previously reported and newly identified DHAA derivatives is that their formation generally involves modifications that increase polarity. While it was not tested in this study, increased polarity can reduce the toxicity of these metabolites relative to the initial DRA by enhancing aqueous solubility and decreasing membrane permeability.^5^ DRAs, in their unmodified form, have been reported to negatively affect microbes,^18^ insects,^19,20^ and aquatic organisms.^5,6^ DRAs in the wastewater of wood processing facilities may affect fish populations in surrounding aquatic ecosystems as these compounds are known to act as fish endocrine disruptors, sometimes causing lethal outcomes. However, oxidized DRA products have been shown to be significantly less toxic for fish and other aquatic species.^21^

Although *P. abietaniphila* was originally isolated from pulp mill effluent, other microbes capable of metabolizing DRAs have been isolated from forest soils and conifer trees.^9^ This suggests that the ability to metabolize DRAs is a trait present in both artificial and natural nutrient-poor niches where these metabolites accumulate. A homologue of the *dit* gene cluster has been identified in *Burkholderia xenovorans* LB400,^22^ which produces several intermediates from DHAA identical to the ones identified here (**2**, **3**), and a similar cluster has been reported in *Pseudomonas diterpeniphila* A19–6a with high homology in several genes,^23^ suggesting that the metabolism catlayzed is very close to that described for *P. abietaniphila*. Previous studies have proposed that the *dit* cluster may be mobile and widely distributed among proteobacterial genomes as a part of a large catabolic transposon.^22^ The full metabolic potential of these gene clusters remains to be uncovered, but their presence across diverse taxa and habitats rich in DRAs points to a possible adaptive advantage in such environments.

## Conclusions

In summary, our findings reveal that *P. abietaniphila* metabolizes DHAA through an extended process that includes both previously characterized and novel intermediates. These transformations expand the known chemical diversity of DHAA-derived metabolites formed during microbial metabolism. The temporal appearance of specific intermediates suggests sequential transformation steps, some of which remain to be elucidated. Altogether, this work expands our understanding of microbial diterpenoid metabolism and provides a foundation for future studies into the biochemical and ecological factors shaping these pathways.

## Methods

### General Experimental Procedures

NMR spectra were acquired on a Bruker Avance III HD 700 MHz spectrometer equipped with a cryoplatform and a 1.7 mm TCI cryoprobe. For spectrometer control and data processing Bruker TopSpin ver. 3.6.1 was used. NMR data were acquired at 25 °C. For all NMR measurements, MeOH-*d*_3_ was used and data were referenced against the residual solvent peaks (δ_H_ 3.31 and δ_C_ 49.15). Metabolite profiling was carried out by UHPLC–HRESIMS on a Dionex Ultimate 3000 system equipped with a Bruker timsTOF mass spectrometer. Bacterial culture extracts were screened for target metabolites using an HPLC instrument (Agilent HP1100 series, consisting of a degasser, binary pump, autosampler and UV diode array detector) coupled to an ion trap mass spectrometer (Bruker Esquire 6000). Preparative HPLC was performed on the same HPLC system equipped with a fraction collector (Advantec SF-2110). DHAA (**1**) was purchased from Fujifilm Wako Pure Chemical Corporation.

### Bacterial Strain and Growth Conditions

*P. abietaniphila* BKME-9 (DSM 17554) was obtained from the DSMZ (German Collection of Microorganisms and Cell Cultures). The strain was cultured in LB medium at 28 °C and 200 rpm for 18 h or on LB agar plates at 28 °C for 18 h.

### Cultivation of *P. abietaniphila* with DHAA

A single colony of *P. abietaniphila* was used to inoculate 25 mL of LB medium in a 100 mL Erlenmeyer flask, which was incubated for 18 h at 28 °C and 200 rpm. Cells were harvested by centrifugation (850*g*, 5 min, 21 °C) in sterile 50 mL tubes. The supernatant was discarded and bacterial pellets were resuspended in 25 mL of a minimal medium supplemented with DHAA as the sole carbon source. This was repeated with three biological replicates.

### Minimal Medium Preparation

The minimal medium was prepared by combining 200 mL of 5× M9 minimal salts (Sigma), 1 mL of 1 M MgSO_4_, 0.3 mL of 1 M CaCl_2_, 1 mL of biotin (1 mg/mL), 1 mL of thiamine (1 mg/mL), 1 mL of a 1000× trace element solution, and 1 mL of DHAA sodium salt stock solution (100 mg/mL). The final volume was adjusted to 1 L with ultrapure water. All stock solutions were individually sterilized by filtration (biotin, thiamin, trace elements, and DHAA sodium salt) or autoclaved (M9 minimal salts, MgSO_4_, CaCl_2_, and ultrapure water). The minimal medium was prepared under sterile conditions in a laminar flow hood. The final concentration of DHAA in the minimal medium was 0.1 mg/mL.

The 1000× trace element solution contained per L: 5.00 g of EDTA, 0.10 g of CuCl_2_·5H_2_O, 2.00 g of FeSO_4_·7H_2_O, 0.10 g of ZnSO_4_·7H_2_O, 0.02 g of NiCl_2_·6H_2_O, 0.20 g of CoCl_2_·6H_2_O, 0.03 g of Na_2_MoO_4_, and 0.03 g of MnCl_2_·4H_2_O. The final volume was brought to 1 L with ultrapure water.

The sodium salt of DHAA was prepared to increase the solubility of DHAA in an aqueous medium. This was produced as described previously,^24^ where 1.33 g of DHAA was dissolved in 12 mL MeOH (0.3 M) and mixed with 372 mg of NaHCO_3_ dissolved in 3 mL of water (0.3 M). The solution was left at room temperature for 14 days and then filtered through a 0.45 μm syringe filter to remove any precipitate.

### Time-Course Experiments

Three replicates of *P. abietaniphila* were cultured as described in the previous section. After resuspension in minimal medium supplemented with DHAA (0.1 mg/mL), cultures were incubated at 28 °C and 200 rpm. During the time course, 1 mL samples were collected every 1 h for 9 h, starting at the time of medium transfer (0 h).

At each time point, under sterile conditions, 1 mL of culture was transferred to a 4 mL glass vial and extracted with 1 mL ethyl acetate by vortexing, followed by centrifugation (4300*g*, 15 min, 21 °C). The organic phase was transferred to a 2 mL glass vial, dried under nitrogen, and resuspended in 0.5 mL methanol for UHPLC-HRESIMS analysis.

To better resolve early time point metabolites, this procedure was repeated as described above, but samples were collected and extracted every 3 h over a 24-h period.

### Metabolite Profiling by UHPLC–HRESIMS

UHPLC–HRESIMS analysis was performed following the general method described by Mueller et al.^25^ with minor modifications. Chromatographic separation was achieved on a ZORBAX Eclipse XDB-C18 column (100 mm × 2.1 mm, 1.8 μm; Agilent Technologies) maintained at 25 °C and operated at a flow rate of 0.3 mL/min. The mobile phases were water with 0.1% formic acid (solvent A) and acetonitrile (solvent B). The gradient was programmed as follows: 10% B from 0–0.5 min, ramped to 90% B from 0.5–11.0 min, then to 100% B from 11.0–11.1 min, held at 100% B until 12.0 min, and re-equilibrated at 10% B from 12.1–15.0 min. Mass spectra were acquired in negative ESI mode with data-dependent MS/MS acquisition. Source parameters were as follows: capillary voltage 3500 V, end plate offset 500 V, nebulizer pressure 2.8 bar, drying gas flow (N_2_) at 8 L/min, and source temperature at 280 °C. Spectra were acquired over an *m*/*z* range of 50–1500 at 12 Hz. Stepped collision energies of 20 and 50 eV were applied. External calibration was performed using 10 μL of a sodium formate-isopropanol solution (10 mM NaOH in 1:1 isopropanol-water with 0.2% formic acid) injected at the beginning of each run.

### Scale-Up Cultivation and Extraction

A starter culture of *P. abietaniphila* was prepared by inoculating 25 mL of LB medium and incubating for 18 h at 28 °C and 200 rpm. For large-scale cultivation, 3 mL of the starter culture was transferred into each of four 1-L Erlenmeyer flasks containing 200 mL of LB medium. The resulting 800 mL of culture was incubated for 24 h under the same conditions, then centrifuged in sterile 250 mL bottles (1900*g*, 15 min, 21 °C). The supernatant was then discarded, and the bacterial pellets from each flask were resuspended in 200 mL of minimal medium supplemented with DHAA (0.1 mg/mL) and returned to the original Erlenmeyer flasks. Cultures were incubated for either 1.5 or 24 h to capture early- or late-stage metabolites, respectively.

After incubation, the four 200 mL cultures were combined and extracted with an equal volume of ethyl acetate by gentle inversion in a separatory funnel. The extraction was repeated, yielding a total of 1600 mL of the ethyl acetate phase. This phase was evaporated to dryness using a rotary evaporator, and the residue was resuspended in 10 mL of methanol.

### Solid-Phase Extraction and HPLC Analysis

The methanol extract was diluted with 90 mL of ultrapure water to obtain a 10% (v/v) methanol solution for solid-phase extraction. A CHROMABOND C18 column (45 mm, 45 mL, 5000 mg; Macherey-Nagel) was conditioned with 45 mL methanol followed by 45 mL ultrapure water. The 100 mL diluted extract was applied to the column and eluted under vacuum. After collecting the flow-through, the column was eluted stepwise with 10 mL aliquots of aqueous methanol ranging from 20% to 100% in 10% increments.

SPE fractions were screened for target metabolites by HPLC-UV-ion trap MS using a C18 reversed-phase column (NUCLEODUR Sphinx RP, 250 mm × 4.6 mm, 5 μm; Macherey-Nagel). Chromatographic separation was performed with a mobile phase consisting of 0.05% formic acid in water (solvent A) and acetonitrile (solvent B), applied in gradient mode at a flow rate of 1.0 mL/min and a column temperature of 25 °C. The gradient was programmed as follows: 50–100% B from 0–10.0 min, held at 100% B from 10.1–12.0 min, and re-equilibrated at 50% B from 12.1–16.0 min. UV detection was carried out across a wavelength range of 190–360 nm. Mass spectrometric detection was performed in full-scan mode (*m*/*z* 80–1200) in alternating positive and negative ionization modes, with the following settings: capillary exit voltage ±113.5 V, capillary voltage +3000/–5000 V, nebulizer pressure 35 psi, drying gas flow 11 L/min, and source temperature 330 °C.

### Preparative HPLC and Compound Isolation

Fractions containing target metabolites were evaporated to dryness using a rotary evaporator and resuspended in 1 mL of methanol for further purification by preparative HPLC on the same C18 column using the same solvent system and gradient as described in the previous section. The flow rate was maintained at 1.0 mL/min and UV absorbance was monitored at 200 nm. Fractions were collected using an automated fraction collector and concentrated again by rotary evaporation. The dried, purified compounds were resuspended in 1.5 mL of methanol and reanalyzed by HPLC-UV-ion trap MS to assess compound purity. Samples were subsequently dried using N_2_ gas and analyzed by NMR spectroscopy for structural elucidation. All compounds were obtained as colorless solids in quantities below 500 μg.

#### Compound **2**, 7β-Hydroxy-dehydroabietic Acid

UV–vis (HPLC) λ_max_ 200 nm; NMR data, see Supporting Table 2; HR-(−)­ESI–MS: *m*/*z* 315.1964 [M–H]^−^ (calcd 315.1960 for C_20_H_27_O_3_), *t*_R_ = 8.7 min.

#### Compound **3**, 7-Oxo-dehydroabietic Acid

UV–vis (HPLC) λ_max_ 210, 254, 302 nm; NMR data, see Supporting Table 2; HR-(−)­ESI–MS: *m*/*z* 313.1808 [M–H]^−^ (calcd 313.1809 for C_20_H_25_O_3_), t_R_ = 9.5 min.

#### Compound **4**, 11,12-Dihydroxy-7-oxoabieta-8,13-dien-18-oic Acid

UV–vis (HPLC) no distinct absorption maxima in the 190–360 nm region; NMR data, see Supporting Table 2; HR-(−)­ESI–MS: *m*/*z* 347.1868 [M–H]^−^ (calcd 347.1864 for C_20_H_27_O_5_), *t*_R_ = 5.2 min.

#### Compound **5**, 5-Hydroxy-dehydroabietic Acid

UV–vis (HPLC) λ_max_ 200 nm; NMR data, see Tables 1 and 2; HR-(−)­ESI–MS: *m*/*z* 315.1964 [M–H]^−^ (calcd 315.1960 for C_20_H_27_O_3_), *t*_R_ = 10.4 min.

**Table 1:** ^1^H NMR Spectroscopic Data (700 MHz, MeOH-*d*_3_) for Compounds **5**–**8**

5α-hydroxy-dehydroabietic acid (5)		5α,7β-dihydroxyabietan-18-oic acid (6)		5α-hydroxy-pimar-8-en-18-oic acid (7)		5α-hydroxy-7-oxo-pimara-8-en-18-oic acid (8)	
position	δ_H_ [ppm], mult. (*J*_HH_ [Hz])	position	δ_H_ [ppm], mult. (*J*_HH_ [Hz])	position	δ_H_ [ppm], mult. (*J*_HH_ [Hz])	position	δ_H_ [ppm], mult. (*J*_HH_ [Hz])
**1α**	2.07, *td* (3.9/13.0/13.0)	**1α**	1.51, *d* (13.0)	**1α**	1.80, *td* (3.0/13.0/13.0)	**1α**	2.03, *td* (4.0/13.0/13.0)
**1β**	1.99, *d* (13.0)	**1β**	1.30, *d* (13.0)	**1β**	1.43, *m*	**1β**	1.50, *d* (13.0)
**2α**	1.71, *m*	**2α**	1.52, *d* (13.0)	**2α**	1.58 *m*	**2α**	1.60, *m*
**2β**	1.88, *m*	**2β**	1.70, *ddddd* (3.9/3.9/13.0/13.0/13.0)	**2β**	1.72, *ddddd* (3.0/3.0/13.0/13.0/13.0)	**2β**	1.80, *ddddd* (3.9/3.9/13.0/13.0/13.0)
**3α**	2.28, *dd* (4.1/13.5)	**3α**	2.22, *ddd* (3.3/13.0/13.0)	**3α**	2.29, *ddd* (3.0/13.0/13.0)	**3α**	2.21, *td* (4.0/13.0/13.0)
**3β**	1.51, *d* (13.5)	**3β**	1.40, *d* (13.0)	**3β**	1.41, *d* (13.0)	**3β**	1.40, *d* (13.0)
**4**	-	**4**	-	**4**	-	**4**	-
**5**	-	**5**	-	**5**	-	**5**	-
**6α**	1.92, *dd* (8.3/13.8)	**6α**	2.02, *dd* (5.3/13.1)	**6α**	1.85, *dd* (7.5/12.8)	**6α**	2.45, *d* (17.8)
**6β**	2.31, *ddd* (8.3/10.5/13.8)	**6β**	1.79, *dd* (10.5/13.1)	**6β**	2.13, *m*	**6β**	2.93, *d* (17.8)
**7α**	3.02, *ddd* (9.0/9.0/17.2)	**7α**	3.59, *ddd* (5.3/10.5/10.5)	**7α**	1.91, *m*	**7**	-
**7β**	2.83, *dd* (8.1/17.2)	**8β**	1.20, *dddd* (3.3/10.5/10.5/10.5)	**7β**	2.13, *m*	**8**	-
**8**	-	**9α**	1.62, *m*	**8**	-	**9**	-
**9**	-	**10**	-	**9**	-	**10**	-
**10**	-	**11α**	1.60, *m*	**10**	-	**11α**	2.23, *m*
**11**	7.09, *d* (8.2)	**11β**	1.01, *m*	**11α**	1.92, *m*	**11β**	2.23, *m*
**12**	6.94, *dd* (1.5/8.1)	**12α**	0.95, *ddd* (2.8/11.9/12.1)	**11β**	1.92, *m*	**12α**	1.45, *m*
**13**	-	**12β**	1.76, *ddd* (2.8/2.9/12.1)	**12α**	1.29, *ddd* (6.0/6.0/12.5)	**12β**	1.35, *m*
**14**	6.87, d (1.5)	**13β**	0.98, *m*	**12β**	1.39, *ddd* (6.0/6.0/12.5)	**13**	-
**15**	2.78, *hept.* (6.9)	**14α**	0.63, *ddd* (10.5/10.5/13.1)	**13**	-	**14α**	1.98, *d* (17.4)
**16**	1.19, *d* (6.7)	**14β**	2.21, *d* (13.1)	**14α**	1.66, *d* (16.8)	**14β**	1.91, *d* (17.4)
**17**	1.19, *d* (6.7)	**15**	1.40, *m*	**14β**	1.61, *d* (16.8)	**15α**	1.22, *m*
**18**	-	**16**	0.88, *d* (6.7)	**15α**	1.20, *m*	**15β**	1.22, *m*
**19**	1.42, *s*	**17**	0.88, *d* (6.7)	**15β**	1.23, *m*	**16**	0.84, *t* (7.6)
**20**	1.28, *s*	**18**	-	**16**	0.83, *t* (7.6)	**17**	0.85, *s*
		**19**	1.35, *s*	**17**	0.82, *s*	**18**	-
		**20**	1.05, *s*	**18**	-	**19**	1.42, *s*
				**19**	1.34, *s*	**20**	1.28, *s*
				**20**	1.16, *s*		

**Table 2:** ^13^C NMR Spectroscopic Data (175 MHz, MeOH-*d*_3_) and Key HMBC Correlations for Compounds **5**–**8**

5α-hydroxy-dehydroabietic acid (5)			5α,7β-dihydroxyabietan-18-oic acid (6)			5α-hydroxy-pimar-8-en-18-oic acid (7)			5α-hydroxy-7-oxo-pimara-8-en-18-oic acid (8)		
position	δ_C_ [ppm], type	HMBC	position	δ_C_ [ppm], type	HMBC	position	δ_C_ [ppm], type	HMBC	position	δ_C_ [ppm], type	HMBC
**1α**	33.6, CH_2_	2, 20, 3, 10, 9	**1α**	32.1, CH_2_	20	**1α**	30.2, CH_2_		**1α**	29.9, CH_2_	2, 20, 10, 9
**1β**	33.6, CH_2_	2, 20, 3, 10, 5	**1β**	32.1, CH_2_	3	**1β**	30.2, CH_2_		**1β**	29.9, CH_2_	2, 20, 3, 10, 5
**2α**	19.1, CH_2_	1, 3, 10	**2α**	18.7, CH_2_		**2α**	18.7, CH_2_		**2α**	18.7, CH_2_	
**2β**	19.1, CH_2_	1, 3	**2β**	18.7, CH_2_		**2β**	18.7, CH_2_		**2β**	18.7, CH_2_	1, 3
**3α**	32.3, CH_2_	2, 19, 1, 4, 18	**3α**	33.0, CH_2_	2, 19, 4	**3α**	33.3, CH_2_	2, 19, 1, 4, 5, 18	**3α**	33.6, CH_2_	2, 19, 4, 18
**3β**	32.3, CH_2_		**3β**	33.0, CH_2_	19	**3β**	33.3, CH_2_		**3β**	33.6, CH_2_	
**4**	51.8, C		**4**	52.2, C		**4**	51.9, C		**4**	51.3, C	
**5**	76.8, C		**5**	78.3, C		**5**	77.6, C		**5**	78.2, C	
**6α**	26.7, CH_2_	7, 10, 4, 5, 8	**6α**	38.7, CH_2_	7, 10, 4, 5, 8	**6α**	26.4, CH_2_	7, 10, 5, 8, 9	**6α**	44.8, CH_2_	7, 10, 4, 5, 8
**6β**	26.7, CH_2_	7, 10, 4, 5	**6β**	38.7, CH_2_	7, 10, 5	**6β**	26.4, CH_2_		**6β**	44.8, CH_2_	7, 10, 5
**7α**	26.6, CH_2_	6, 14, 8, 9	**7α**	73.3, CH	14, 6, 8	**7α**	28.9, CH_2_		**7**	201.1, C	
**7β**	26.6, CH_2_	6, 5, 14, 8, 9	**8β**	44.8, CH	7	**7β**	28.9, CH_2_	8, 9	**8**	129.3, C	
**8**	135.6, C		**9α**	46.4, CH	20, 11, 12, 10	**8**	126.4, C		**9**	165.3, C	
**9**	146.8, C		**10**	41.5, C		**9**	134.7, C		**10**	45.6, C	
**10**	43.2, C		**11α**	26.1, CH_2_	9	**10**	43.8, C		**11α**	23.7, CH_2_	
**11**	124.8, CH	10, 8, 13	**11β**	26.1, CH_2_		**11α**	22.1, CH_2_		**11β**	23.7, CH_2_	
**12**	124.7, CH	15, 14, 9	**12α**	31.1, CH_2_		**11β**	22.1, CH_2_		**12α**	33.7, CH_2_	11, 13, 15, 14, 9
**13**	146.1, C		**12β**	31.1, CH_2_	11, 13, 9	**12α**	35.0, CH_2_		**12β**	33.7, CH_2_	11, 13, 15, 14, 9
**14**	127.2, CH	6, 15, 12, 9	**13β**	44.9, CH		**12β**	35.0, CH_2_		**13**	31.3, C	
**15**	34.7, CH	16/17, 12, 14, 13	**14α**	35.1, CH_2_	13, 7	**13**	32.1, C		**14α**	35.1, CH_2_	8, 9, 13, 15, 17
**16**	24.3, CH_3_	17, 15, 13	**14β**	35.1, CH_2_	12	**14α**	43.8 CH_2_		**14β**	35.1, CH_2_	8, 9, 13, 15, 17
**17**	24.3, CH_3_	16, 15, 13	**15**	34.3, CH	13	**14β**	43.8, CH_2_		**15α**	33.9, CH_2_	16, 17, 14, 13
**18**	183.9, C		**16**	20.3, CH_3_	17, 13, 15	**15α**	35.0, CH_2_		**15β**	33.9, CH_2_	16, 17, 14, 13
**19**	20.1, CH_3_	3, 4, 5, 18	**17**	20.3, CH_3_	16, 13, 15	**15β**	35.0, CH_2_		**16**	8.0, CH_3_	13, 12
**20**	29.7, CH_3_	1, 10, 5, 9	**18**	183.1, C		**16**	8.3, CH_3_	13, 12, 15	**17**	24.1, CH_3_	13, 12, 14
			**19**	20.2, CH_3_	3, 4, 5	**17**	24.0, CH_3_	13, 12, 15, 14	**18**	184.9, C	
			**20**	17.8, CH_3_	1, 10, 5, 9	**18**	182.2, C		**19**	20.1, CH_3_	3, 4, 5, 18
						**19**	20.1, CH_3_	3, 4, 5, 18	**20**	23.5, CH_3_	1, 10, 5, 9
						**20**	24.6, CH_3_	1, 10, 5, 9			

#### Compound **6**, 5,7β-Dihydroxyabietan-18-oic Acid

UV–vis (HPLC) λ_max_ 202 nm; NMR data, see Tables 1 and 2; HR-(−)­ESI–MS: *m*/*z* 337.2383 [M–H]^−^ (calcd 337.2379 for C_20_H_33_O_4_), *t*_R_ = 9.8 min.

#### Compound **7**, 5-Hydroxy-pimara-8-en-18-oic Acid

UV–vis (HPLC) no distinct absorption maxima in the 190–360 nm region; NMR data, see Tables 1 and 2; HR-(−)­ESI–MS: *m*/*z* 319.2280 [M–H]^−^ (calcd 319.2273 for C_20_H_31_O_3_), *t*_R_ = 12.1 min.

#### Compound **8**, 5-Hydroxy-7-oxo-pimara-8-en-18-oic Acid

UV–vis (HPLC) λ_max_ 254 nm; NMR data, see Tables 1 and 2; HR-(−)­ESI–MS: *m*/*z* 333.2067 [M–H]^−^ (calcd 333.2066 for C_20_H_29_O_4_), *t*_R_ = 9.0 min.

## Supplementary Material



## Notes

LC–MS chromatograms; growth curves; time-course metabolite profiles; UV absorption spectra; full NMR data sets for compounds **2**–**8**, including ^1^H, COSY, HSQC, HMBC, ROESY, and DEPTQ spectra, as well as structural correlation diagrams; and chemical-shift tables (PDF)
