# Supplementary material for: Temporal Metabolomics Reveals Additional Diterpene Resin Acid Metabolites in Pseudomonas abietaniphila
Source: ACS Omega. 2026 Mar 28;11(13):21190–9. doi: 10.1021/acsomega.6c00600 (PMC13063091; doi:10.1021/acsomega.6c00600)
Supplement: Supplementary file 1 [file ao6c00600_si_001.pdf]

Supporting Information

Temporal Metabolomics Reveals Additional Diterpene Resin Acid Metabolites in *Pseudomonas abietaniphila*

AUTHOR ADDRESS

*Kristina Kshatriya [a], Christian Paetz [b], Jonathan Gershenzon [a], and Axel Schmidt [a]**

[a] Department of Biochemistry, Max Planck Institute for Chemical Ecology, 07745 Jena, Germany

[b] NMR/Biosynthesis Group, Max Planck Institute for Chemical Ecology, 07745 Jena, Germany

* Email: aschmidt@ice.mpg.de

TABLE OF CONTENTS

Supplemental Figure 1. LC-MS base peak chromatograms (BPCs) of *P. abietaniphila* incubated for 24 h in either LB or minimal medium (MM) supplemented with DHAA (**1**). Bacteria grown in LB did not metabolize DHAA (**1**), whereas bacteria incubated in MM completely metabolized DHAA (**1**) and produced two other compounds at this timepoint (**6** and **7**)....... 11

Supplemental Figure 2. Growth of *P. abietaniphila* (PA) cultures after 24 h of inoculation in different media. OD_{600} measurements were taken of cultures grown in LB or minimal medium +/- DHAA (**1**). Two different inoculation methods (PA 1 and PA 2) were tested. NC, negative control....... 11

Supplemental Figure 3. Time-course analysis of *P. abietaniphila* DHAA (**1**) metabolites **2** – **8**. Bacterial cultures were harvested every 3 h over a 24-h period and analyzed by HRESIMS. Error bars represent the standard deviation among three biological replicates at each time point....... 12

Supplemental Figure 4. UV absorption spectra of compounds **2**, **3**, **5**, **6**, and **8** obtained from HPLC-DAD. Spectra were taken at the peak apex of each compound from the corresponding chromatographic run. Compounds **4** and **7** are not shown due to insufficient UV signal. 13

Supplemental Figure 5. Structure elucidation of **2**—**8**, NMR key correlations for the elucidation of ring A 14

Supplemental Figure 6. Structure elucidation of **2**, overview 15

Supplemental Figure 7. Structure elucidation of **2**, ^{1}H-NMR spectrum, full range 15

Supplemental Figure 8. Structure elucidation of **2**, ^{1}H-NMR spectrum, detail aromatic range 16

Supplemental Figure 9. Structure elucidation of **2**, ^{1}H-NMR spectrum, detail low field aliphatic range 16

Supplemental Figure 10. Structure elucidation of **2**, ^{1}H-NMR spectrum, detail high field aliphatic range 17

Supplemental Figure 11. Structure elucidation of **2**, comparison ^{1}H-NMR spectrum, positions 1 to 3, vs. SELTOCSY with transmitter frequency on H-1β 17

Supplemental Figure12. Structure elucidation of **2**, ^{1}H-^{1}H COSY spectrum, full range....... 18

Supplemental Figure 13. Structure elucidation of **2**, ^{1}H-^{1}H COSY spectrum, detail aromatic range 18

Supplemental Figure 14. Structure elucidation of **2**, ^{1}H-^{1}H COSY spectrum, detail aliphatic range 19

Supplemental Figure 15. Structure elucidation of **2**, ^{1}H-^{1}H COSY spectrum, detail aliphatic range, positions 1 to 3 (SELTOCSY projections)....... 19

Supplemental Figure 16. Structure elucidation of **2**, ^{1}H-^{1}H ROESY spectrum, full range 20
Supplemental Figure 17. Structure elucidation of **2**, ^{1}H-^{1}H ROESY spectrum, detail aromatic range 20
Supplemental Figure 18. Structure elucidation of **2**, ^{1}H-^{1}H ROESY spectrum, detail methyl correlations 21
Supplemental Figure 19. Structure elucidation of **2**, structure of **2** with important ROESY correlations of β-oriented substituents 21
Supplemental Figure 20. Structure elucidation of **2**, ^{1}H-^{1}H ROESY spectrum, detail H-5α correlations 22
Supplemental Figure 21. Structure elucidation of **2**, structure of **2** with important ROESY correlations of α-oriented substituents 22
Supplemental Figure 22. Structure elucidation of **2**, ^{1}H-^{13}C HSQC spectrum, full range...... 23
Supplemental Figure 23. Structure elucidation of **2**, ^{1}H-^{13}C HSQC spectrum, detail aromatic range 23
Supplemental Figure 24. Structure elucidation of **2**, ^{1}H-^{13}C HSQC spectrum, detail aliphatic range. Gray rectangles indicate impurities and the solvent signal. 24
Supplemental Figure 25. Structure elucidation of **2**, ^{1}H-^{13}C HSQC spectrum, detail low field aliphatic range 24
Supplemental Figure 26. Structure elucidation of **2**, ^{1}H-^{13}C HSQC spectrum, detail methyl range 25
Supplemental Figure 27. Structure elucidation of **2**, ^{1}H-^{13}C HMBC spectrum, full range 25
Supplemental Figure 28. Structure elucidation of **2**, ^{1}H-^{13}C HMBC spectrum, detail aromatic range 26
Supplemental Figure 29. Structure elucidation of **2**, structure of **2** with fragments elucidated from correlations in the aromatic range 26
Supplemental Figure 30. Structure elucidation of **2**, ^{1}H-^{13}C HMBC spectrum, detail correlations from H-7α 27
Supplemental Figure 31. Structure elucidation of **2**, structure of **2** with fragments elucidated from correlations of H-7α 27
Supplemental Figure 32. Structure elucidation of **2**, ^{1}H-^{13}C HMBC spectrum, detail aliphatic range. Gray rectangles indicate impurities. 28
Supplemental Figure 33. Structure elucidation of **2**, structure of **2** with fragments elucidated from correlations in the aliphatic range........ 28
Supplemental Figure 34. Structure elucidation of **2**, ^{1}H-^{13}C HMBC spectrum, detail methyl range 29

Supplemental Figure 35. Structure elucidation of **2**, structure of **2** with fragments elucidated from correlations in the methyl range 29

Supplemental Figure 36. Structure elucidation of **3**, overview 30

Supplemental Figure 37. Structure elucidation of **3**, ^{1}H-NMR spectrum, full range 30

Supplemental Figure 38. Structure elucidation of **3**, ^{1}H-NMR spectrum, detail aromatic range 31

Supplemental Figure 39. Structure elucidation of **3**, ^{1}H-NMR spectrum, detail low field aliphatic range 31

Supplemental Figure 40. Structure elucidation of **3**, ^{1}H-NMR spectrum, detail high field aliphatic range 32

Supplemental Figure 41. Structure elucidation of **3**, comparison ^{1}H-NMR spectrum, detail positions 1 to 3 32

Supplemental Figure 42. Structure elucidation of **3**, ^{1}H-^{1}H COSY spectrum, full range........ 33

Supplemental Figure 43. Structure elucidation of **3**, ^{1}H-^{1}H COSY spectrum, detail aromatic range 33

Supplemental Figure 44. Structure elucidation of **3**, ^{1}H-^{1}H COSY spectrum, detail aliphatic range 34

Supplemental Figure 45. Structure elucidation of **3**, ^{1}H-^{1}H COSY spectrum, detail aliphatic range, positions 1 to 3 34

Supplemental Figure 46. Structure elucidation of **3**, ^{1}H-^{1}H ROESY spectrum, full range 35

Supplemental Figure 47. Structure elucidation of **3**, ^{1}H-^{1}H ROESY spectrum, detail aromatic range 35

Supplemental Figure 48. Structure elucidation of **3**, ^{1}H-^{1}H ROESY spectrum, detail methyl correlations 36

Supplemental Figure 49. Structure elucidation of **3**, structure of **3** with important ROESY correlations of β-oriented substituents 36

Supplemental Figure 50. Structure elucidation of **3**, ^{1}H-^{13}C HSQC spectrum, full range........ 37

Supplemental Figure 51. Structure elucidation of **3**, ^{1}H-^{13}C HSQC spectrum, detail aromatic range 37

Supplemental Figure 52. Structure elucidation of **3**, ^{1}H-^{13}C HSQC spectrum, detail aliphatic range 38

Supplemental Figure 53. Structure elucidation of **3**, ^{1}H-^{13}C HSQC spectrum, detail methyl range 38

Supplemental Figure 54. Structure elucidation of **3**, ^{1}H-^{13}C HMBC spectrum, full range 39

Supplemental Figure 55. Structure elucidation of **3**, $^{1}H-^{13}C$ HMBC spectrum, detail aromatic range 39

Supplemental Figure 56. Structure elucidation of **3**, structure of **3** with fragments elucidated from $^{1}H-^{13}C$ HMBC correlations in the aromatic range 40

Supplemental Figure 57. Structure elucidation of **3**, $^{1}H-^{13}C$ HMBC spectrum, detail low field aliphatic range 40

Supplemental Figure 58. Structure elucidation of **3**, structure of **3** with fragments elucidated from $^{1}H-^{13}C$ HMBC correlations in the low field aliphatic range 41

Supplemental Figure 59. Structure elucidation of **3**, $^{1}H-^{13}C$ HMBC spectrum, detail resonances ring A 41

Supplemental Figure 60. Structure elucidation of **3**, structure of **3** with fragments elucidated from $^{1}H-^{13}C$ HMBC correlations of resonances in ring A 42

Supplemental Figure 61. Structure elucidation of **3**, $^{1}H-^{13}C$ HMBC spectrum, detail methyl range 42

Supplemental Figure 62. Structure elucidation of **3**, structure of **3** with fragments elucidated from HMBC correlations in the methyl range 43

Supplemental Figure 63. Structure elucidation of **4**, overview 43

Supplemental Figure 64. Structure elucidation of **4**, ^{1}H-NMR spectrum, full range. The intensity of the signals for position 11 and 12 was decreased because of the applied water suppression. 44

Supplemental Figure 65. Structure elucidation of **4**, ^{1}H-NMR spectrum, detail resonances ring A. The upper spectrum is a SELTOCY with the transmitter set on resonance with H-1β. The positions have been assigned based on signal shapes and chemical shifts determined for the other compounds described in this publication (Supplemental Table 1). 44

Supplemental Figure 66. Structure elucidation of **4**, $^{1}H-^{1}H$ COSY spectrum, detail aliphatic range 45

Supplemental Figure 67. Structure elucidation of **4**, $^{1}H-^{1}H$ COSY spectrum, detail aliphatic range, positions 1 to 3. A SELTOCY with the transmitter set on resonance with H-1β is used for the projections in F1 and F2. 45

Supplemental Figure 68. Structure elucidation of **4**, $^{1}H-^{13}C$ HSQC spectrum, detail aliphatic range. An aliphatic impurity (*) appears exactly on the position for the methyl resonance CH_3-19, which leads to cancellation of the signal. 46

Supplemental Figure 69. Structure elucidation of **4**, $^{1}H-^{13}C$ HMBC spectrum, detail aliphatic range. An aliphatic impurity (*) appears exactly on the position for the methyl resonance CH_3-19, which leads to cancellation of the signal and its correlations. 46

Supplemental Figure 70. Structure elucidation of **5**, overview 47

Supplemental Figure 71. Structure elucidation of **5**, ^{1}H-NMR spectrum, full range 47

Supplemental Figure 72. Structure elucidation of **5**, ^{13}C-NMR data. The spectrum represents the combined projections from ^{1}H-^{13}C HSQC and ^{1}H-^{13}C HMBC correlations...................... 48

Supplemental Figure 73. Structure elucidation of **5**, ^{1}H-^{1}H COSY spectrum, full range....... 48

Supplemental Figure 74. Structure elucidation of **5**, ^{1}H-^{1}H COSY spectrum, detail aromatic range 49

Supplemental Figure 75. Structure elucidation of **5**, ^{1}H-^{1}H COSY spectrum, detail aliphatic range 49

Supplemental Figure 76. Structure elucidation of **5**, ^{1}H-^{1}H ROESY spectrum, full range 50

Supplemental Figure 77. Structure elucidation of **5**, ^{1}H-^{1}H ROESY spectrum, detail aromatic range 50

Supplemental Figure 78. Structure elucidation of **5**, ^{1}H-^{1}H ROESY spectrum, detail aliphatic range 51

Supplemental Figure 79. Structure elucidation of **5**, ^{1}H-^{1}H ROESY spectrum, detail methyl correlations 51

Supplemental Figure 80. Structure elucidation of **5**, structure of **5** with important ROESY correlations of β-oriented substituents 52

Supplemental Figure 81. Structure elucidation of **5**, ^{1}H-^{13}C HSQC spectrum, full range...... 52

Supplemental Figure 82. Structure elucidation of **5**, ^{1}H-^{13}C HSQC spectrum, detail aromatic range 53

Supplemental Figure 83. Structure elucidation of **5**, ^{1}H-^{13}C HSQC spectrum, detail aliphatic range 53

Supplemental Figure 84. Structure elucidation of **5**, ^{1}H-^{13}C HSQC spectrum, detail methyl range 54

Supplemental Figure 85. Structure elucidation of **5**, ^{1}H-^{13}C HMBC spectrum, full range 54

Supplemental Figure 86. Structure elucidation of **5**, ^{1}H-^{13}C HMBC spectrum, detail aromatic range 55

Supplemental Figure 87. Structure elucidation of **5**, structure of **5** with fragments elucidated from ^{1}H-^{13}C HMBC correlations in the aromatic range... 55

Supplemental Figure 88. Structure elucidation of **5**, ^{1}H-^{13}C HMBC spectrum, detail low field aliphatic range 56

Supplemental Figure 89. Structure elucidation of **5**, structure of **5** with fragments elucidated from ^{1}H-^{13}C HMBC correlations in the low field aliphatic range... 56

Supplemental Figure 90. Structure elucidation of **5**, ^{1}H-^{13}C HMBC spectrum, detail resonances ring A, high field ^{13}C range 57

Supplemental Figure 91. Structure elucidation of **5**, ^{1}H-^{13}C HMBC spectrum, detail resonances ring A, low field ^{13}C range 57

Supplemental Figure 92. Structure elucidation of **5**, structure of **5** with fragments elucidated from HMBC correlations of resonances in ring A 58

Supplemental Figure 93. Structure elucidation of **5**, ^{1}H-^{13}C HMBC spectrum, detail methyl range 58

Supplemental Figure 94. Structure elucidation of **5**, structure of **5** with fragments elucidated from ^{1}H-^{13}C HMBC correlations in the methyl range 59

Supplemental Figure 95. Structure elucidation of **6**, overview 59

Supplemental Figure 96. Structure elucidation of **6**, ^{1}H-NMR spectrum, full range 60

Supplemental Figure 97. Structure elucidation of **6**, ^{1}H-NMR spectrum, detail resonances of rings B and C. On top the SELTOCSY spectrum with transmitter frequency of H-7α 60

Supplemental Figure 98. Structure elucidation of **6**, ^{1}H-NMR spectrum, detail ring A. On top the SELTOCSY spectrum with transmitter frequency set on H-2β 61

Supplemental Figure 99. Structure elucidation of **6**, ^{1}H-^{1}H COSY spectrum, full range 61

Supplemental Figure 100. Structure elucidation of **6**, ^{1}H-^{1}H COSY spectrum, detail positions 1 to 3. A SELTOCSY with the transmitter set on H-2β is used for projection in F1 and F2 ... 62

Supplemental Figure 101. Structure elucidation of **6**, ^{1}H-^{1}H COSY spectrum, detail resonances of rings B and C. A SELTOCSY with the transmitter set on H-7α is used for projection in F1 and F2 62

Supplemental Figure 102. Structure elucidation of **6**, ^{1}H-^{1}H COSY spectrum, detail aliphatic range 63

Supplemental Figure 103. Structure elucidation of **6**, structure of **6** with fragments elucidated from ^{1}H-^{1}H COSY correlations 63

Supplemental Figure 104. Structure elucidation of **6**, ^{1}H-^{1}H ROESY spectrum, full range .. 64

Supplemental Figure 105. Structure elucidation of **6**, ^{1}H-^{1}H ROESY spectrum, detail correlations for H-8β. Signals from impurities are covered (grey) 64

Supplemental Figure 106. Structure elucidation of **6**, structure of **6** with important ROESY correlations of β-oriented substituents of rings B and C 65

Supplemental Figure 107. Structure elucidation of **6**, ^{1}H-^{1}H ROESY spectrum, detail correlations for H-14α. The covered signals (red) represent COSY-like correlations 65

Supplemental Figure 108. Structure elucidation of **6**, structure of **6** with important ROESY correlations of α-oriented substituents of rings B and C 66

Supplemental Figure 109. Structure elucidation of **6**, ^{13}C-DEPTQ spectrum 66

Supplemental Figure 110. Structure elucidation of **6**, ^{1}H-^{13}C HSQC spectrum, full range.... 67

Supplemental Figure 111. Structure elucidation of **6**, ^{1}H-^{13}C HSQC spectrum, detail.......... 67

Supplemental Figure 112. Structure elucidation of **6**, ^{1}H-^{13}C HMBC spectrum, full range .. 68

Supplemental Figure 113. Structure elucidation of **6**, ^{1}H-^{13}C HMBC spectrum, detail aliphatic range (δ_H 4—2).. 68

Supplemental Figure 114. Structure elucidation of **6**, structure of **6** with fragments elucidated from HMBC correlations in the aliphatic range (δ_H 4—2) 69

Supplemental Figure 115. Structure elucidation of **6**, ^{1}H-^{13}C HMBC spectrum, detail aliphatic range (δ_H 2—1.5).. 69

Supplemental Figure 116. Structure elucidation of **6**, structure of **6** with fragments elucidated from HMBC correlations in the aliphatic range (δ_H 2—1.5) 70

Supplemental Figure 117. Structure elucidation of **6**, ^{1}H-^{13}C HMBC spectrum, detail aliphatic range (δ_H 1.5—1.15).. 70

Supplemental Figure 118. Structure elucidation of **6**, structure of **6** with fragments elucidated from HMBC correlations in the aliphatic range (δ_H 1.5—1.15) 71

Supplemental Figure 119. Structure elucidation of **6**, ^{1}H-^{13}C HMBC spectrum, detail aliphatic range (δ_H 1.15—0.5).. 71

Supplemental Figure 120. Structure elucidation of **6**, structure of **6** with fragments elucidated from HMBC correlations in the aliphatic range (δ_H 1.15—0.5) 72

Supplemental Figure 121. Structure elucidation of **7**, numbering of **7**.................................. 72

Supplemental Figure 122. Structure elucidation of **7**, overview 73

Supplemental Figure 123. Structure elucidation of **7**, ^{1}H-NMR spectrum, full range. Asterisks mark impurities that increase the integrals.. 73

Supplemental Figure 124. Structure elucidation of **7**, comparison ^{1}H-NMR spectrum, detail positions 1 to 3. The upper spectrum is the SELTOCSY with the transmitter set on resonance with H-3α 74

Supplemental Figure 125. Structure elucidation of **7**, comparison ^{1}H-NMR spectrum, detail positions 6 and 7. The upper spectrum is the SELTOCSY with the transmitter set on resonance with H-6α 74

Supplemental Figure 126. Structure elucidation of **7**, comparison ^{1}H-NMR spectrum, detail resonances ring C. The upper spectrum is the SELTOCSY with the transmitter set on resonance with H-14αβ.. 75

Supplemental Figure 127. Structure elucidation of **7**, comparison ^{1}H-NMR spectrum, detail side chain. The upper spectrum is the SELTOCSY with the transmitter set on resonance with H-15ab.. 75

Supplemental Figure 128. Structure elucidation of **7**, ^{1}H-^{1}H COSY spectrum, full range..... 76

Supplemental Figure 129. Structure elucidation of **7**, ^{1}H-^{1}H COSY spectrum, detail aliphatic range, ring A. The SELTOCSY with the transmitter set on resonance with H-3α is used for the F1 and F2 projections. 76

Supplemental Figure 130. Structure elucidation of **7**, ^{1}H-^{1}H COSY spectrum, detail aliphatic range, ring B. The SELTOCSY with the transmitter set on resonance with H-6α is used for the F1 and F2 projections. 77

Supplemental Figure 131. Structure elucidation of **7**, ^{1}H-^{1}H COSY spectrum, detail aliphatic range, ring C. The SELTOCSY with the transmitter set on resonance with H-14αβ is used for the F1 and F2 projections. 77

Supplemental Figure 132. Structure elucidation of **7**, ^{1}H-^{1}H ROESY spectrum, full range .. 78

Supplemental Figure 133. Structure elucidation of **7**, structure of **7** with important ROESY correlations 78

Supplemental Figure 134. Structure elucidation of **7**, ^{13}C-DEPTQ spectrum 79

Supplemental Figure 135. Structure elucidation of **7**, ^{1}H-^{13}C HSQC spectrum, full range.... 79

Supplemental Figure 136. Structure elucidation of **7**, ^{1}H-^{13}C HMBC spectrum, full range .. 80

Supplemental Figure 137. Structure elucidation of **7**, ^{1}H-^{13}C HMBC spectrum, detail aliphatic range (δ_H 2.4—2) 80

Supplemental Figure 138. Structure elucidation of **7**, structure of **7** with fragments elucidated from HMBC correlations in the aliphatic range (δ_H 2.4—2) 81

Supplemental Figure 139. Structure elucidation of **7**, ^{1}H-^{13}C HMBC spectrum, detail aliphatic range (δ_H 1.5—0.7) 81

Supplemental Figure 140. Structure elucidation of **7**, structure of **7** with fragments elucidated from HMBC correlations in the aliphatic range (δ_H 1.5—0.7) 82

Supplemental Figure 141. Structure elucidation of **8**, overview 82

Supplemental Figure 142. Structure elucidation of **8**, ^{1}H-NMR spectrum, full range 83

Supplemental Figure 143. Structure elucidation of **8**, comparison ^{1}H-NMR spectrum, detail positions 1 to 3. The upper spectrum is a SELTOCSY with the transmitter set on resonance with H-2β. 83

Supplemental Figure 144. Structure elucidation of **8**, ^{1}H-^{1}H COSY spectrum, full range..... 84

Supplemental Figure 145. Structure elucidation of **8**, ^{1}H-^{1}H COSY spectrum, detail aliphatic range (δ_H 2.3—1.3) 84

Supplemental Figure 146. Structure elucidation of **8**, ^{1}H-^{1}H ROESY spectrum, full range .. 85

Supplemental Figure 147. Structure elucidation of **8**, ^{1}H-^{1}H ROESY spectrum, detail aliphatic range (δ_H 3.0—2.1) 85

Supplemental Figure 148. Structure elucidation of **8**, ^{1}H-^{1}H ROESY spectrum, correlations from CH_3-17 86

Supplemental Figure 149. Structure elucidation of **8**, structure of **8** with important ROESY correlations 86

Supplemental Figure 150. Structure elucidation of **8**, ^{1}H-^{13}C HSQC spectrum, full range.... 87

Supplemental Figure 151. Structure elucidation of **8**, ^{1}H-^{13}C HSQC spectrum, detail methyl range 87

Supplemental Figure 152. Structure elucidation of **8**, ^{1}H-^{13}C HSQC spectrum, detail aliphatic range 88

Supplemental Figure 153. Structure elucidation of **8**, ^{1}H-^{13}C HMBC spectrum, full range .. 88

Supplemental Figure 154. Structure elucidation of **8**, ^{1}H-^{13}C HMBC spectrum, detail aliphatic range (δ_H 3.1—2.3) 89

Supplemental Figure 155. Structure elucidation of **8**, structure of **8** with fragments elucidated from HMBC correlations in the aliphatic range (δ_H 3.1—2.3) 89

Supplemental Figure 156. Structure elucidation of **8**, ^{1}H-^{13}C HMBC spectrum, detail aliphatic range (δ_H 2.4—1.65), high field F1 range 90

Supplemental Figure 157. Structure elucidation of **8**, ^{1}H-^{13}C HMBC spectrum, detail aliphatic range (δ_H 2.4—1.65), low field F1 range 90

Supplemental Figure 158. Structure elucidation of **8**, structure of **8** with fragments elucidated from HMBC correlations in the aliphatic range (δ_H 2.4—1.65) 91

Supplemental Figure 159. Structure elucidation of **8**, ^{1}H-^{13}C HMBC spectrum, detail aliphatic range (δ_H 1.5—1.15), high field F1 range 91

Supplemental Figure 160. Structure elucidation of **8**, ^{1}H-^{13}C HMBC spectrum, detail aliphatic range (δ_H 1.5—1.15), low field F1 range 92

Supplemental Figure 161. Structure elucidation of **8**, structure of **8** with fragments elucidated from HMBC correlations in the aliphatic range (δ_H 1.5—1.15) 93

Supplemental Figure 162. Structure elucidation of **8**, ^{1}H-^{13}C HMBC spectrum, detail aliphatic range (δ_H 1.0—0.7), low field F1 range 93

Supplemental Figure 163. Structure elucidation of **8**, structure of **8** with fragments elucidated from HMBC correlations in the aliphatic range (δ_H 1.0—0.7) 93

Supplemental Table 1. Chemical shifts of positions 1—3 in ring A of compound **2**—**8** 94

Supplemental Table 2. NMR spectroscopic data (700 MHz, MeOH-*d*$_3$) for DHAA derivatives (**2**–**4**). 95

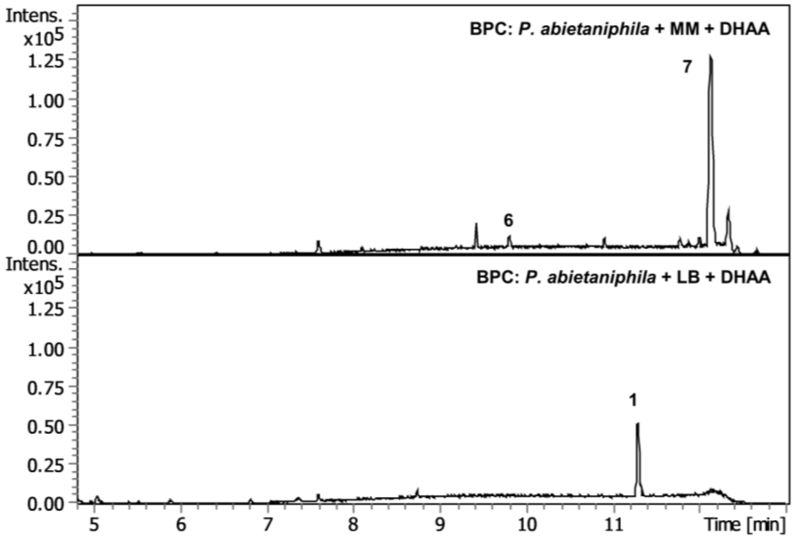

Supplemental Figure 1. LC-MS base peak chromatograms (BPCs) of *P. abietaniphila* incubated for 24 h in either LB or minimal medium (MM) supplemented with DHAA (**1**). Bacteria grown in LB did not metabolize DHAA (**1**), whereas bacteria incubated in MM completely metabolized DHAA (**1**) and produced two other compounds at this timepoint (**6** and **7**).

OD_{600} measurements

**Media Type**	**NC**	**plus PA 1**	**plus PA 2**
LB	-0.002	1.561	2.063
LB + DHAA	-0.003	1.571	2.142
Minimal media	0.001	0.026	1.824
Minimal media + DHAA	0.000	0.049	1.827

PA 1 = 100 µL of PA in LB added to 2.9 mL media
PA 2 = 3 mL of PA in LB centrifuged and resuspended in 3 mL media

Supplemental Figure 2. Growth of *P. abietaniphila* (PA) cultures after 24 h of inoculation in different media. OD_{600} measurements were taken of cultures grown in LB or minimal medium +/- DHAA (**1**). Two different inoculation methods (PA 1 and PA 2) were tested. NC, negative control.

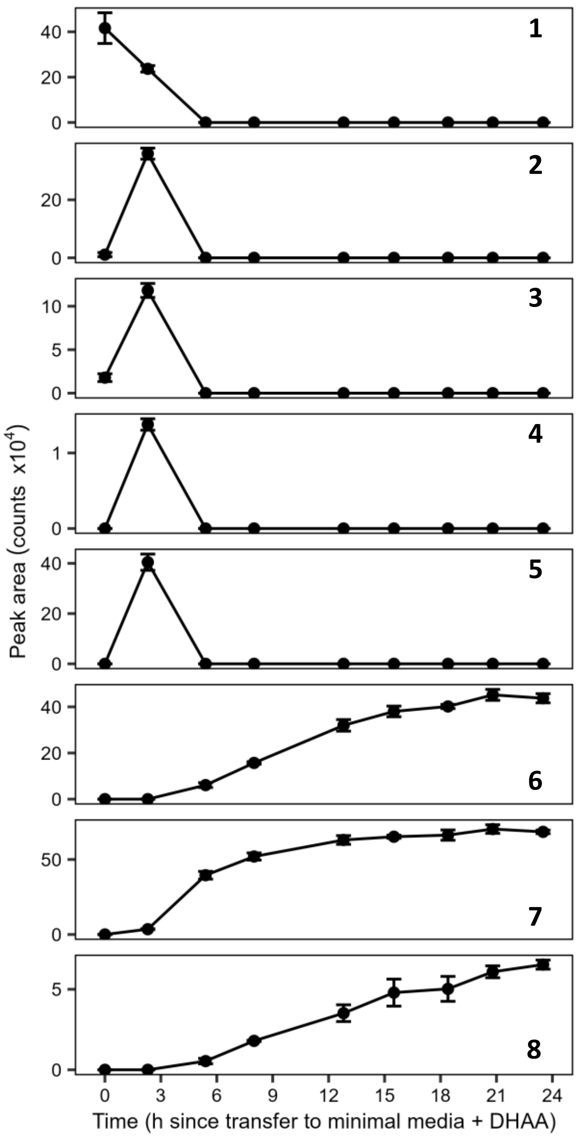

Supplemental Figure 3. Time-course analysis of *P. abietaniphila* DHAA (**1**) metabolites **2** – **8**. Bacterial cultures were harvested every 3 h over a 24-h period and analyzed by HRESIMS. Error bars represent the standard deviation among three biological replicates at each time point.

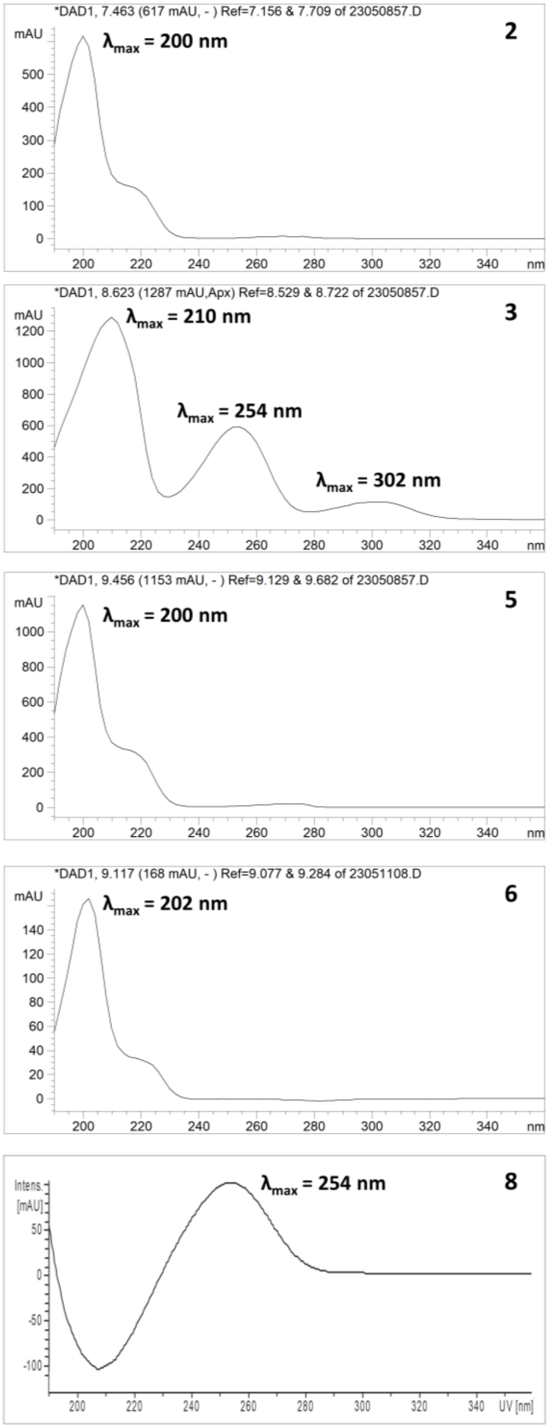

Supplemental Figure 4. UV absorption spectra of compounds **2**, **3**, **5**, **6**, and **8** obtained from HPLC-DAD. Spectra were taken at the peak apex of each compound from the corresponding chromatographic run. Compounds **4** and **7** are not shown due to insufficient UV signal.

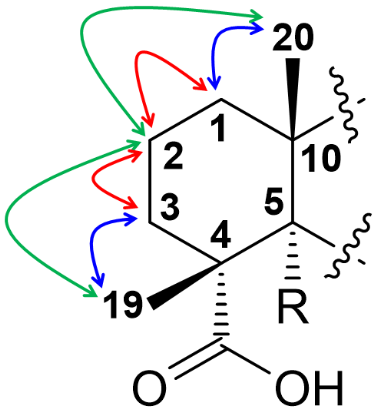

- Strong correlation of $H_{1\alpha}$ to C_{20} and of $H_{3\alpha}$ to C_{19} in HMBC
- Strong correlation of $H_{2\beta}$ to $H_{1\alpha}$ and $H_{3\alpha}$ in COSY
- Strong correlation of $H_{2\beta}$ to H_{20} and H_{19} in ROESY

Supplemental Figure 5. Structure elucidation of **2**—**8**, NMR key correlations for the elucidation of ring A

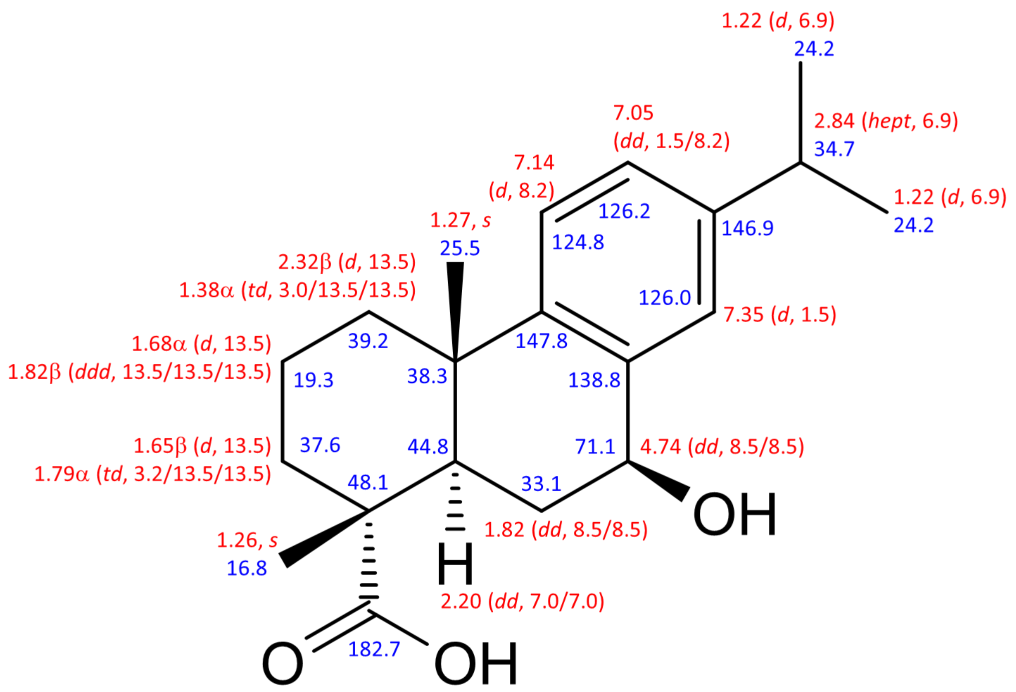

Supplemental Figure 6. Structure elucidation of **2**, overview

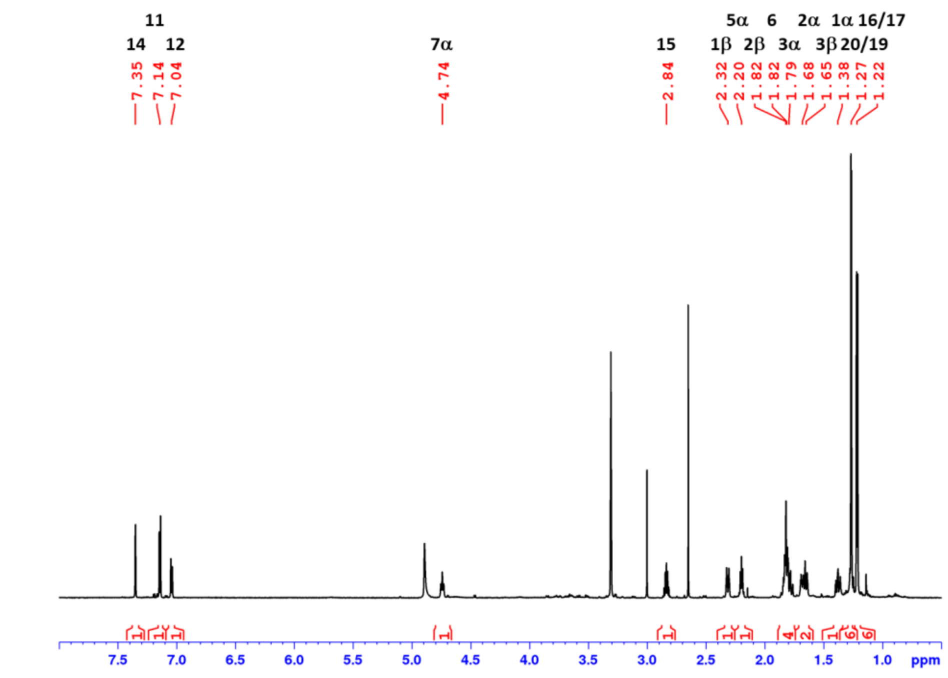

Supplemental Figure 7. Structure elucidation of **2**, ^{1}H-NMR spectrum, full range

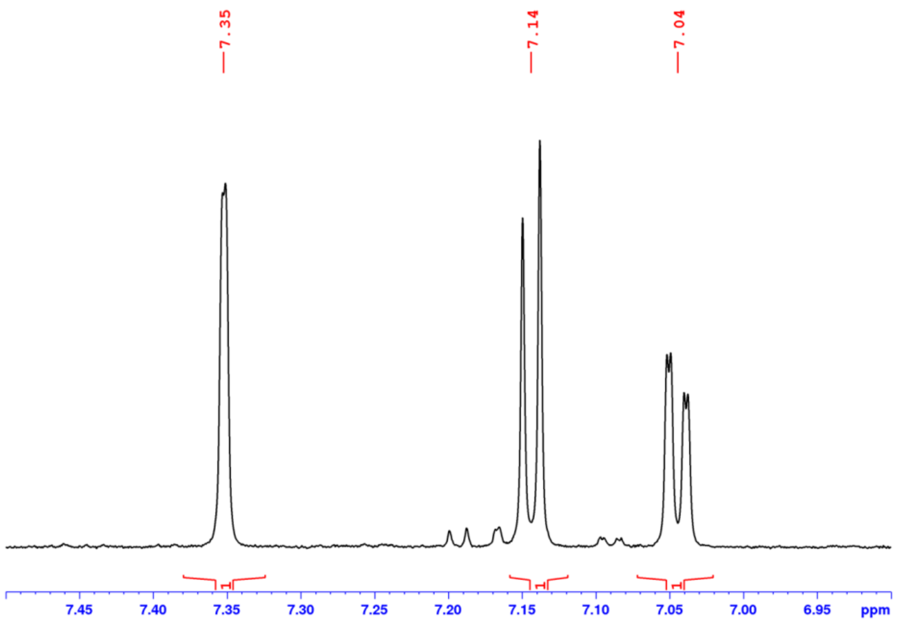

Supplemental Figure 8. Structure elucidation of **2**, ^{1}H-NMR spectrum, detail aromatic range

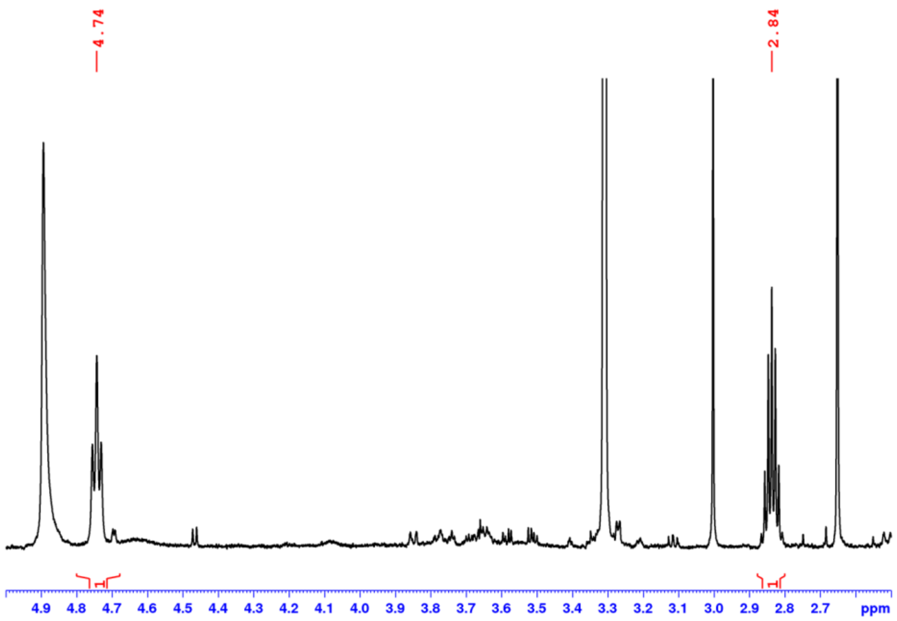

Supplemental Figure 9. Structure elucidation of **2**, ^{1}H-NMR spectrum, detail low field aliphatic range

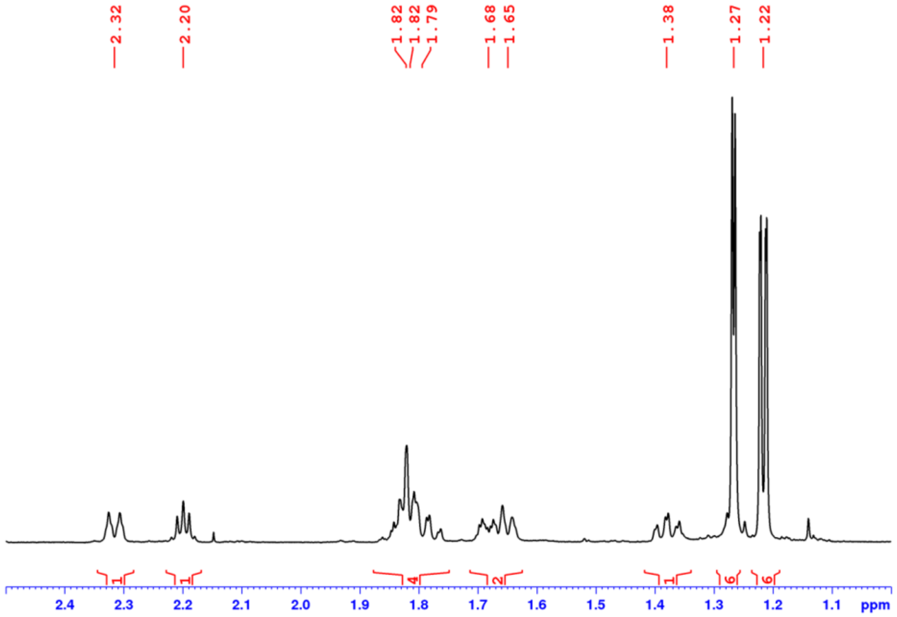

Supplemental Figure 10. Structure elucidation of **2**, ^{1}H-NMR spectrum, detail high field aliphatic range

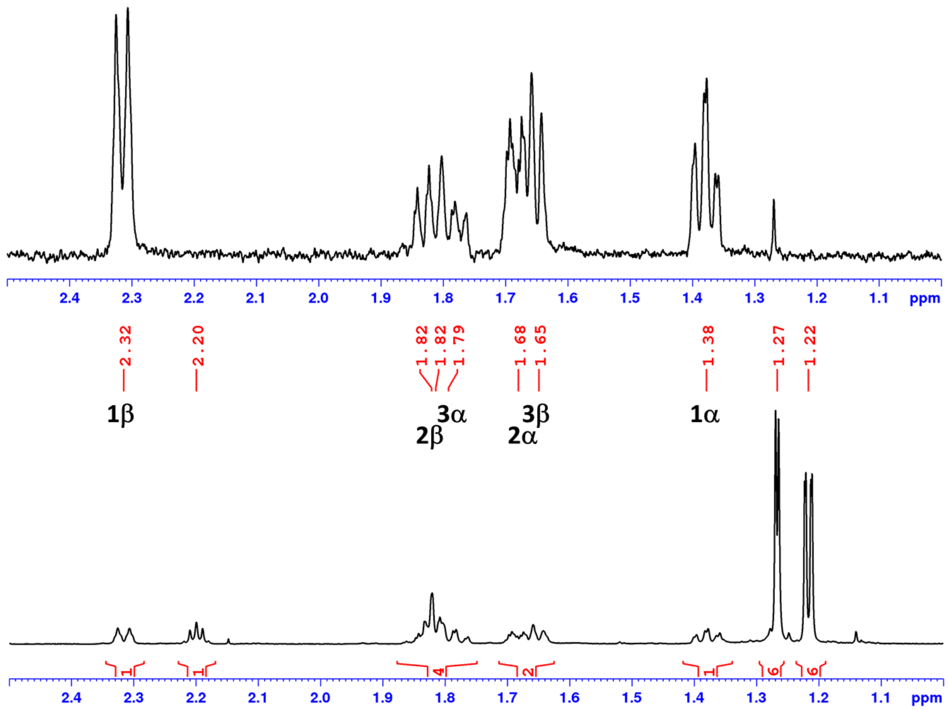

Supplemental Figure 11. Structure elucidation of **2**, comparison ^{1}H-NMR spectrum, positions 1 to 3, vs. SELTOCSY with transmitter frequency on H-1β

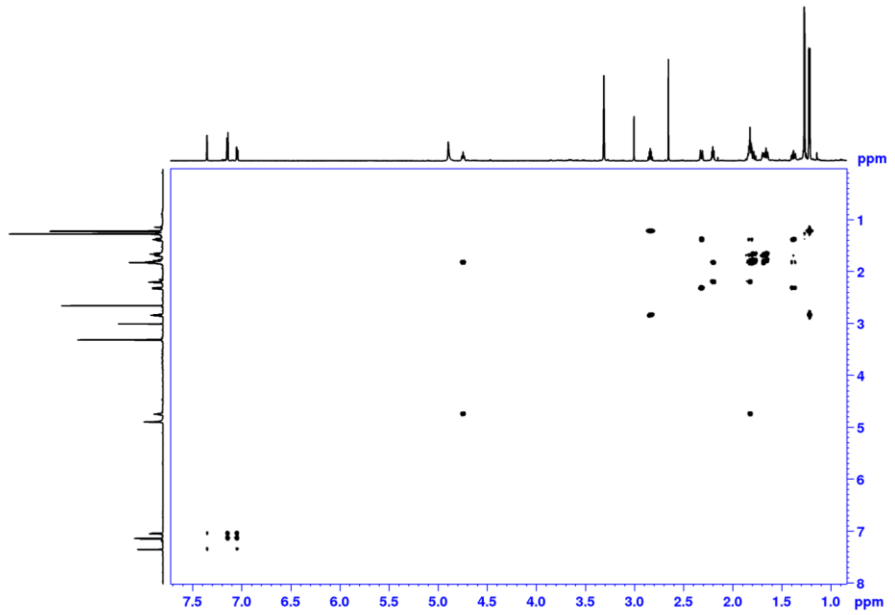

Supplemental Figure12. Structure elucidation of **2**, ^{1}H-^{1}H COSY spectrum, full range

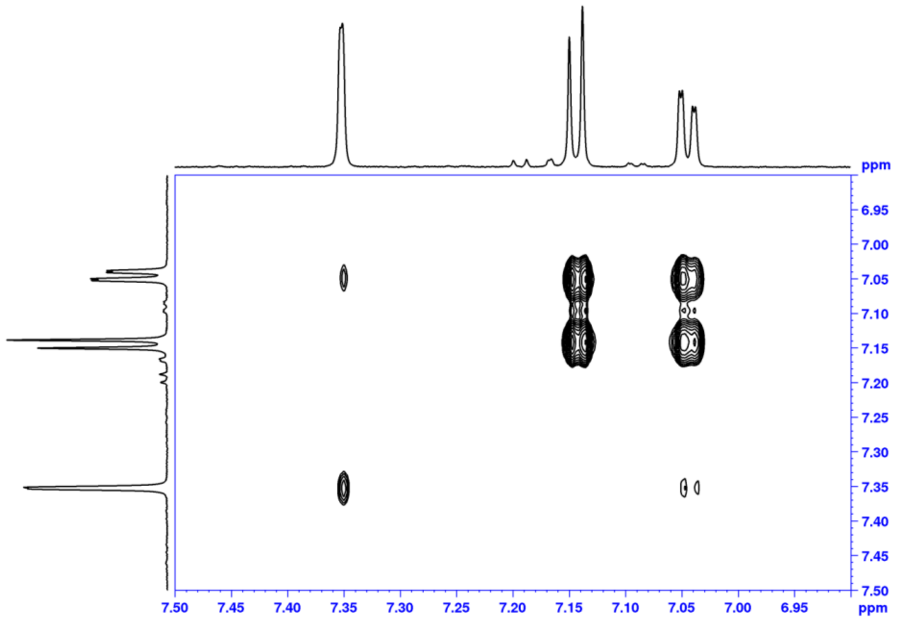

Supplemental Figure 13. Structure elucidation of **2**, ^{1}H-^{1}H COSY spectrum, detail aromatic range

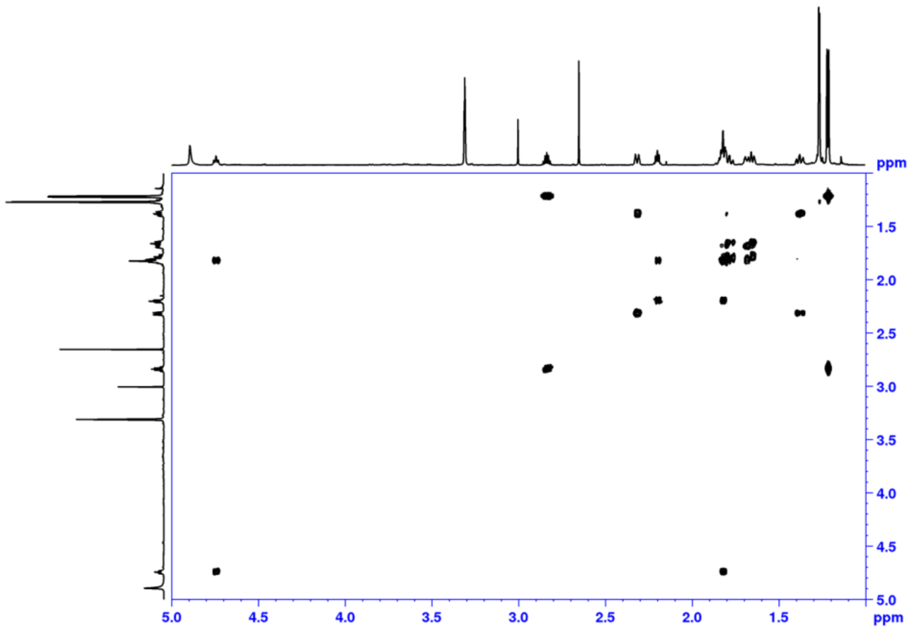

Supplemental Figure 14. Structure elucidation of **2**, ^{1}H-^{1}H COSY spectrum, detail aliphatic range

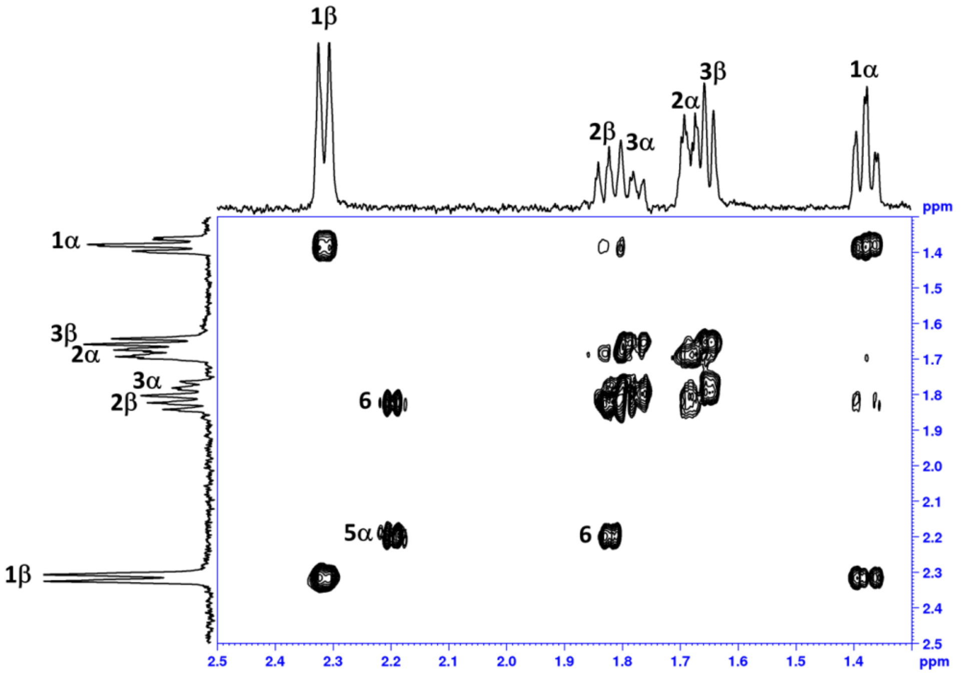

Supplemental Figure 15. Structure elucidation of **2**, ^{1}H-^{1}H COSY spectrum, detail aliphatic range, positions 1 to 3 (SELTOCSY projections)

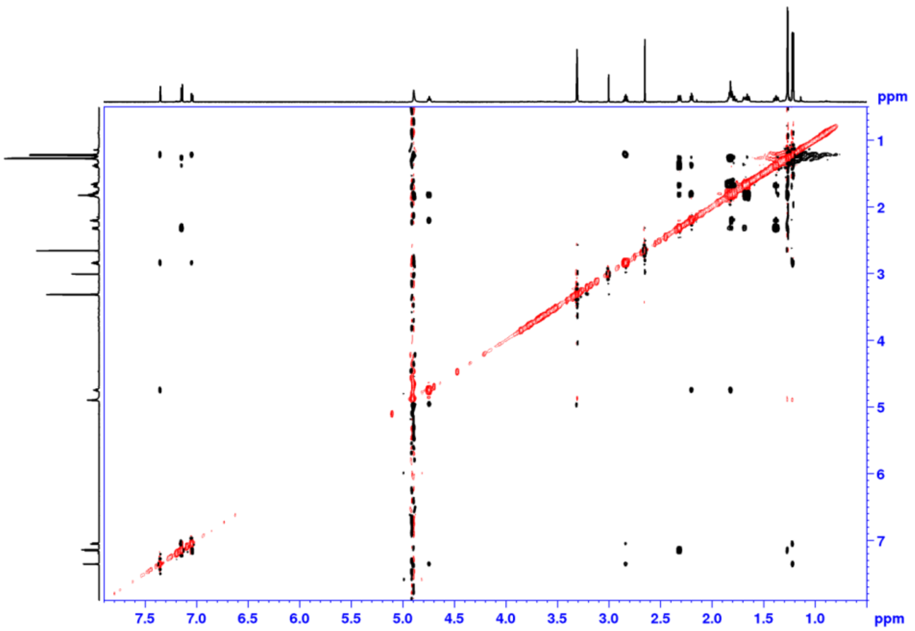

Supplemental Figure 16. Structure elucidation of **2**, ^{1}H-^{1}H ROESY spectrum, full range

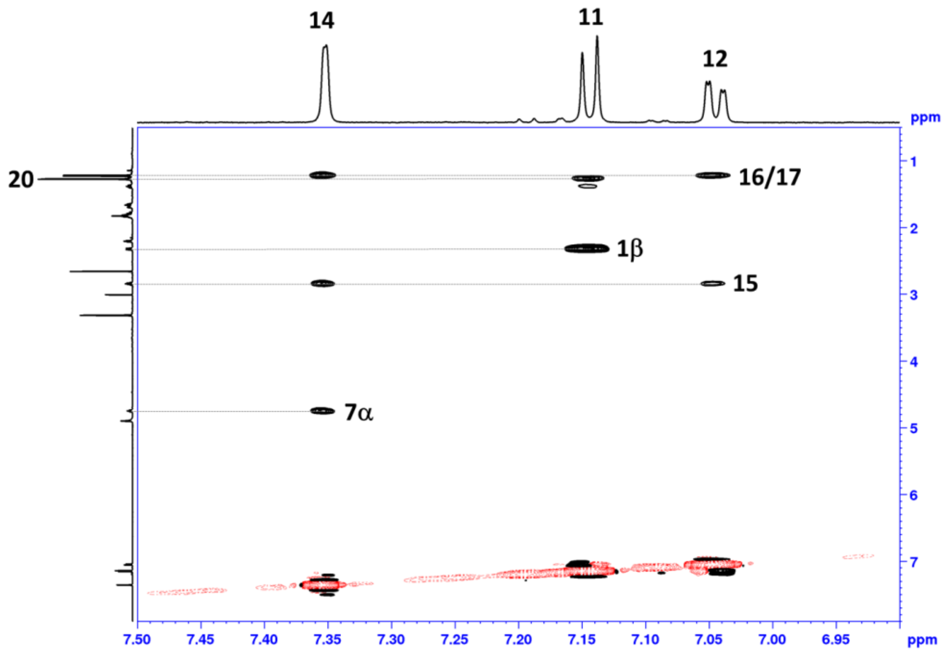

Supplemental Figure 17. Structure elucidation of **2**, ^{1}H-^{1}H ROESY spectrum, detail aromatic range

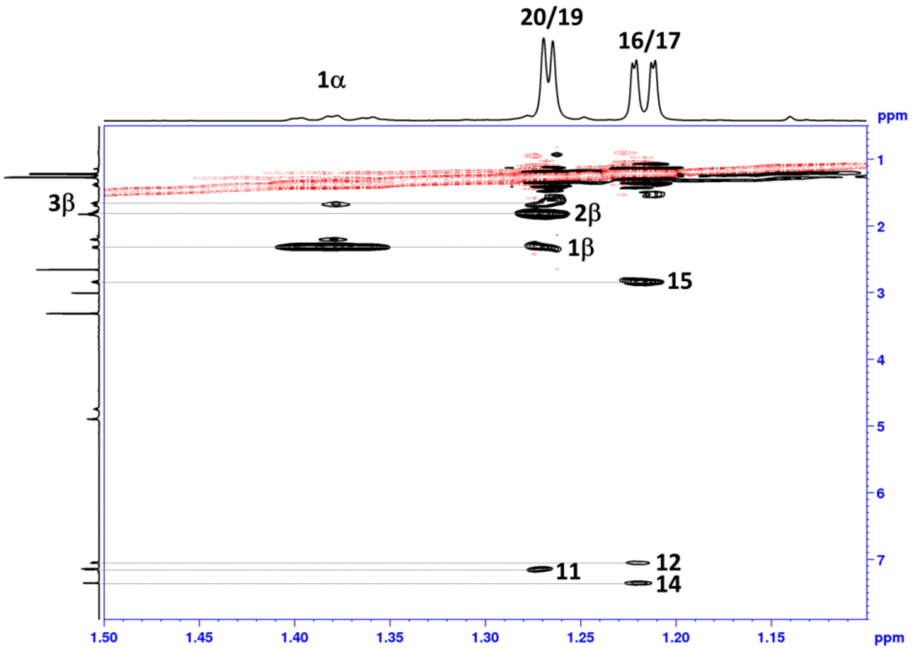

Supplemental Figure 18. Structure elucidation of **2**, ^{1}H-^{1}H ROESY spectrum, detail methyl correlations

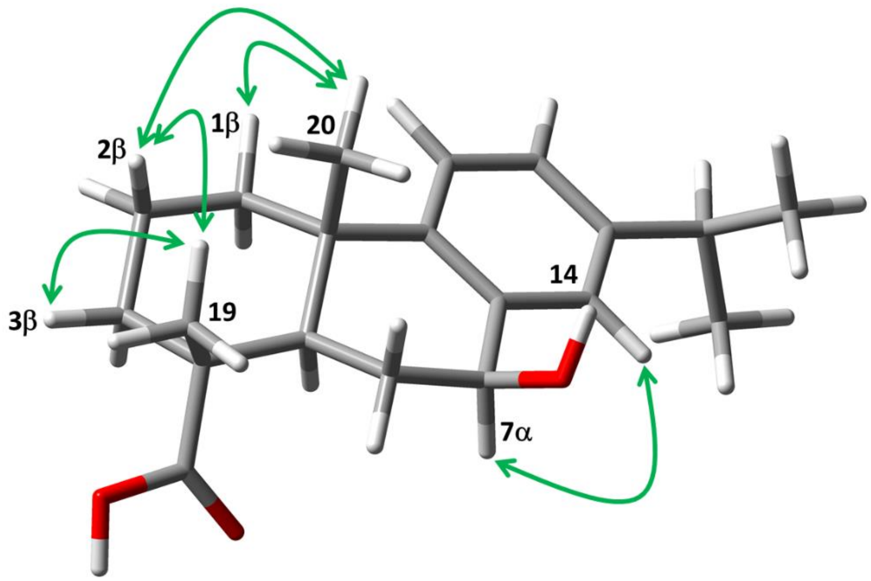

Supplemental Figure 19. Structure elucidation of **2**, structure of **2** with important ROESY correlations of β-oriented substituents

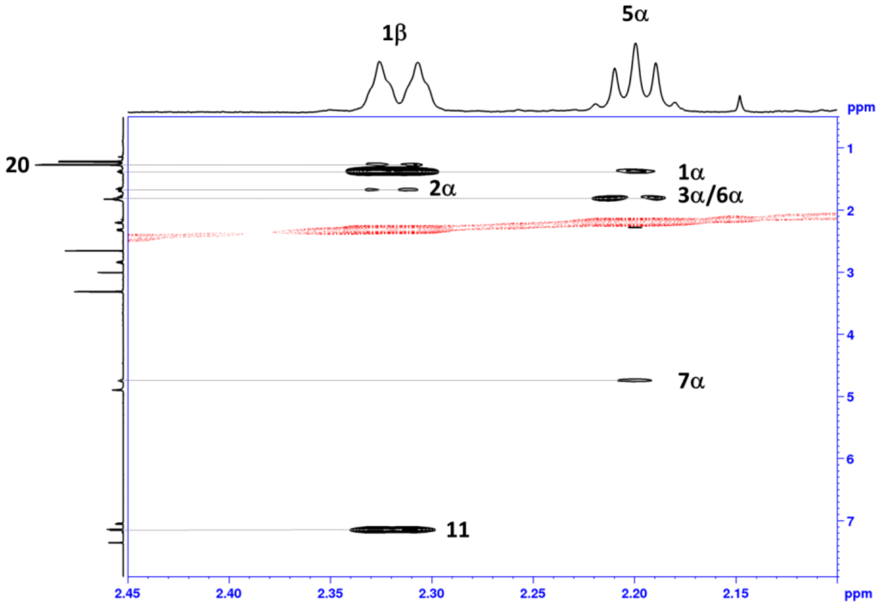

Supplemental Figure 20. Structure elucidation of **2**, ^{1}H-^{1}H ROESY spectrum, detail H-5α correlations

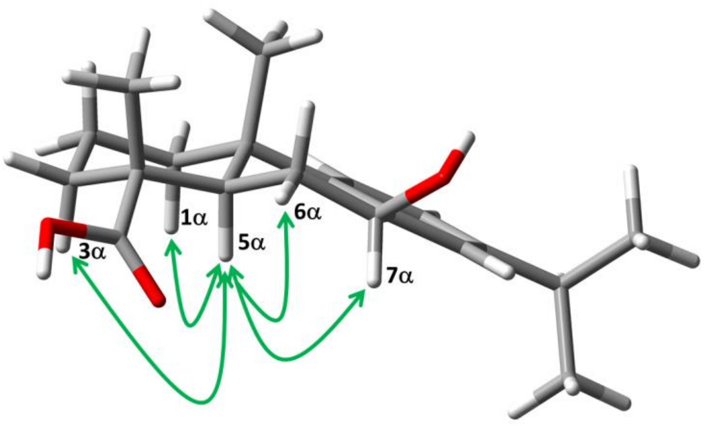

Supplemental Figure 21. Structure elucidation of **2**, structure of **2** with important ROESY correlations of α-oriented substituents

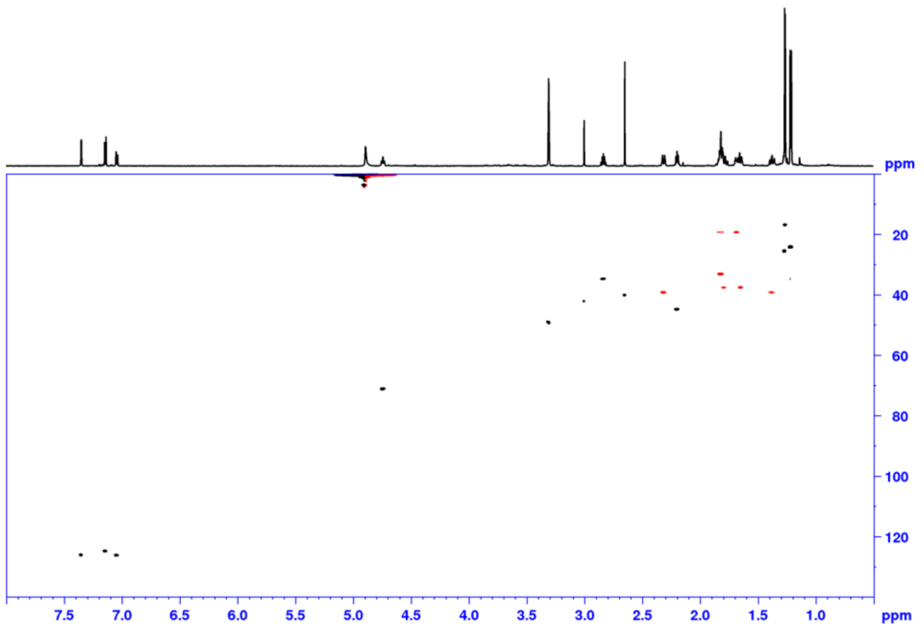

Supplemental Figure 22. Structure elucidation of **2**, ^{1}H-^{13}C HSQC spectrum, full range

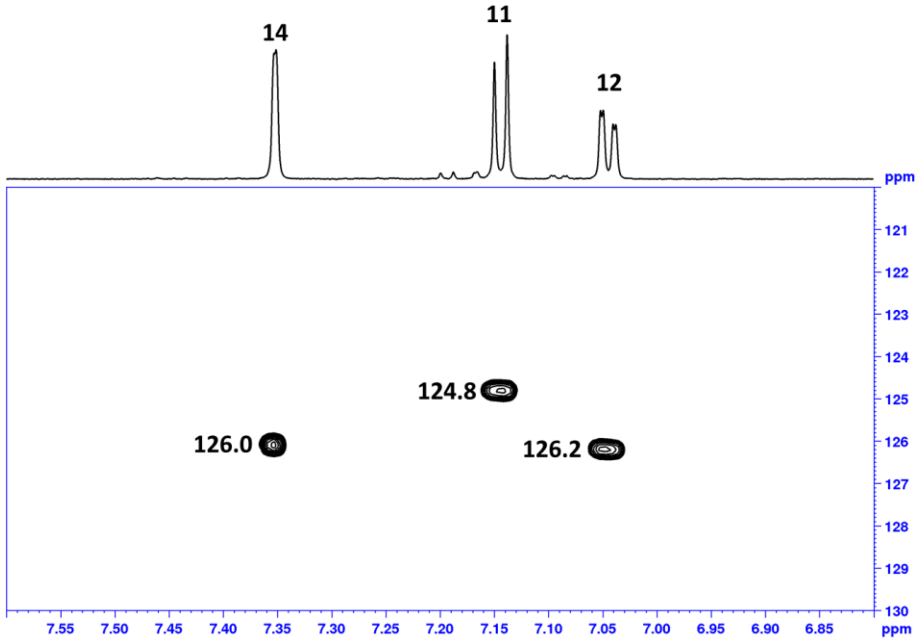

Supplemental Figure 23. Structure elucidation of **2**, ^{1}H-^{13}C HSQC spectrum, detail aromatic range

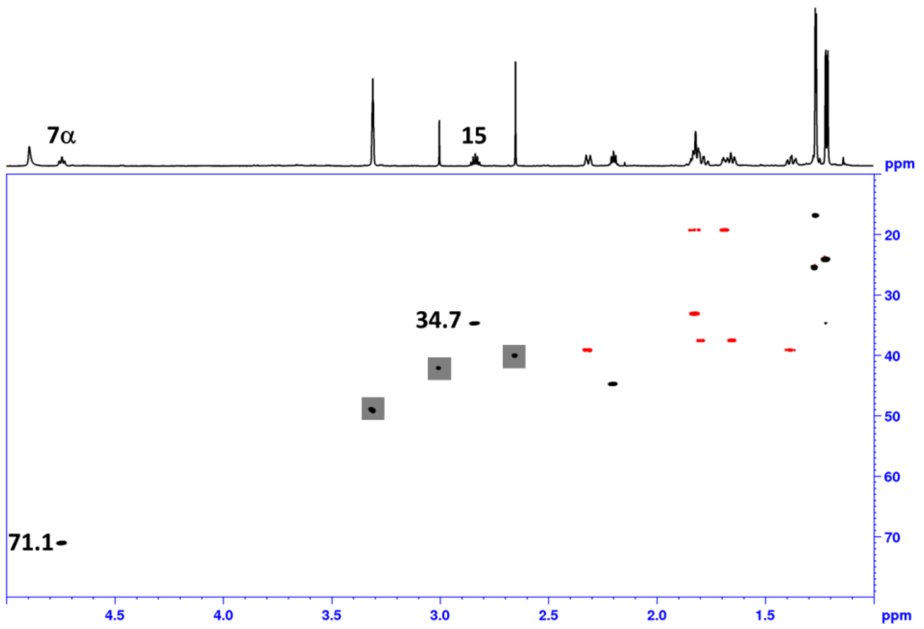

Supplemental Figure 24. Structure elucidation of **2**, ^{1}H-^{13}C HSQC spectrum, detail aliphatic range. Gray rectangles indicate impurities and the solvent signal.

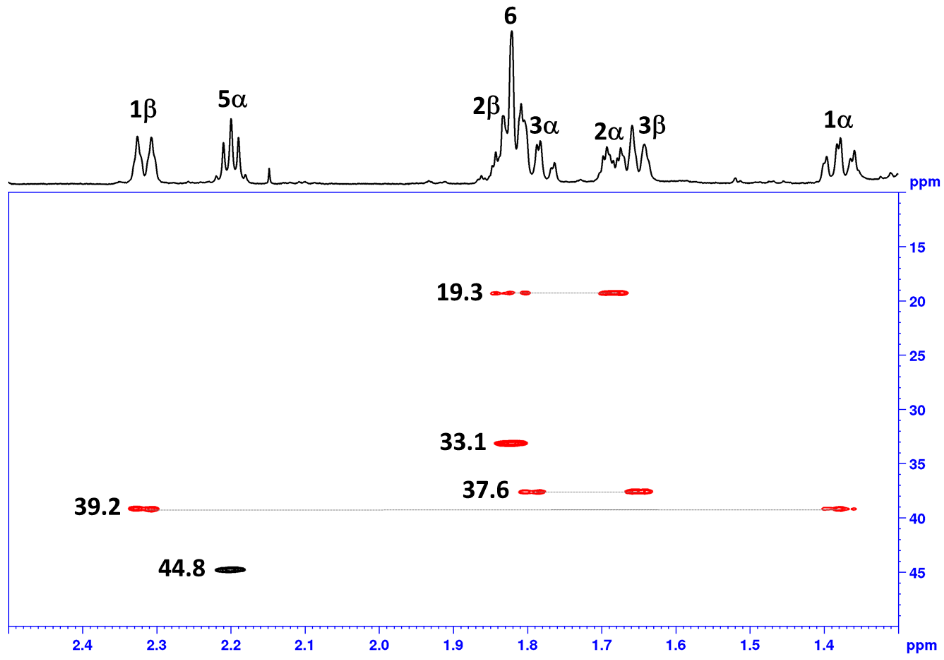

Supplemental Figure 25. Structure elucidation of **2**, ^{1}H-^{13}C HSQC spectrum, detail low field aliphatic range

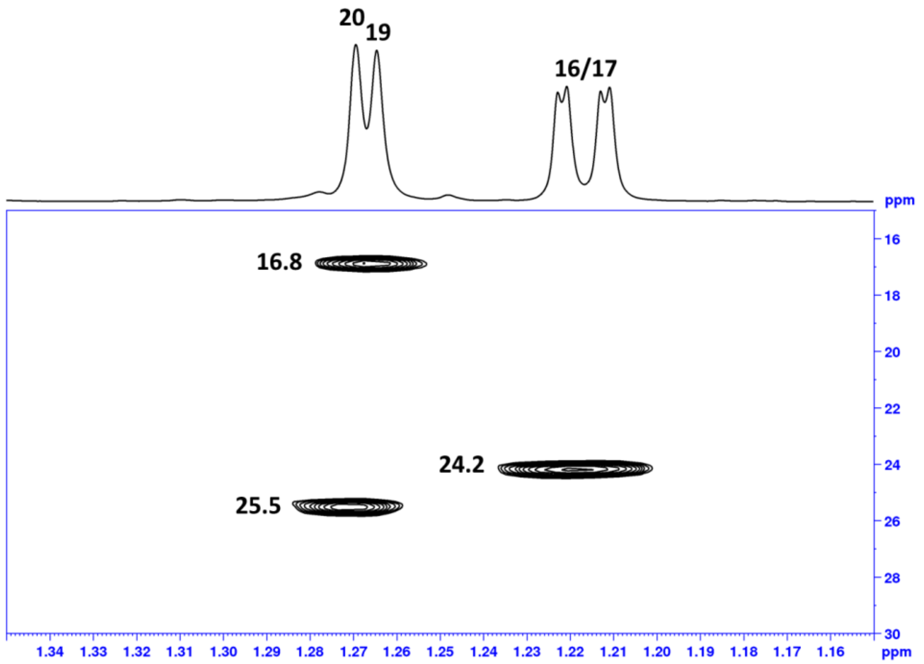

Supplemental Figure 26. Structure elucidation of **2**, ^{1}H-^{13}C HSQC spectrum, detail methyl range

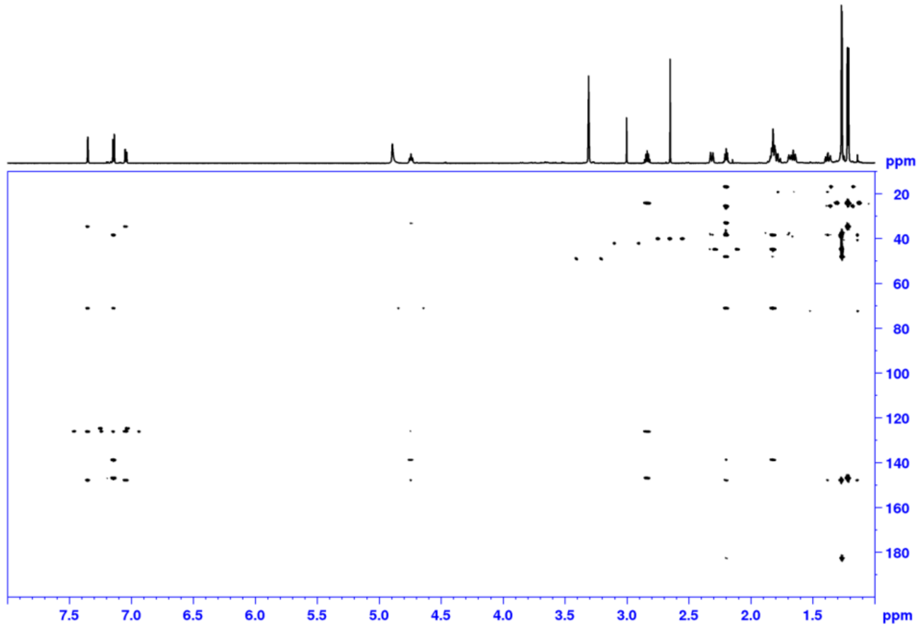

Supplemental Figure 27. Structure elucidation of **2**, ^{1}H-^{13}C HMBC spectrum, full range

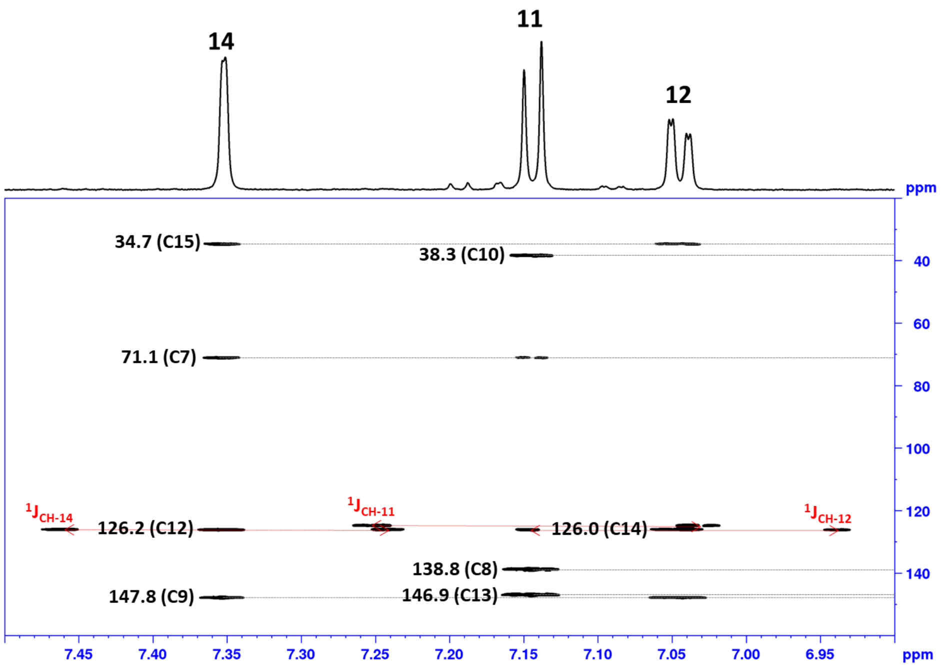

Supplemental Figure 28. Structure elucidation of **2**, ^{1}H-^{13}C HMBC spectrum, detail aromatic range

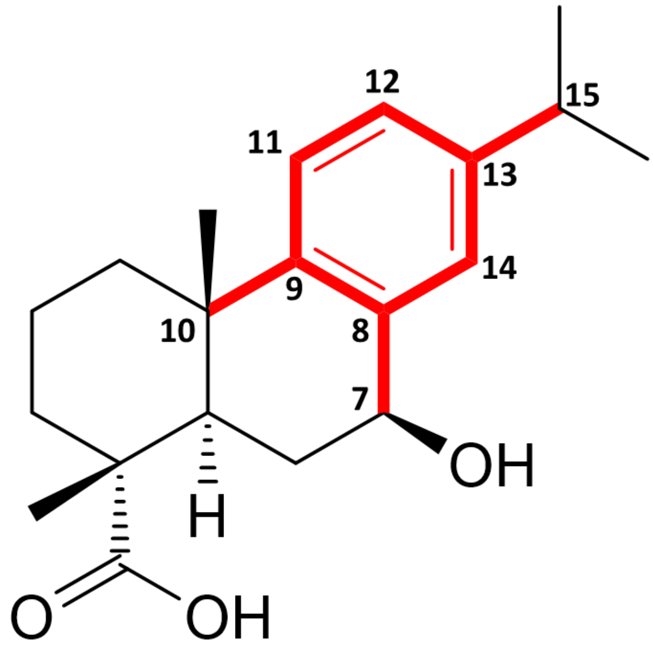

Supplemental Figure 29. Structure elucidation of **2**, structure of **2** with fragments elucidated from correlations in the aromatic range

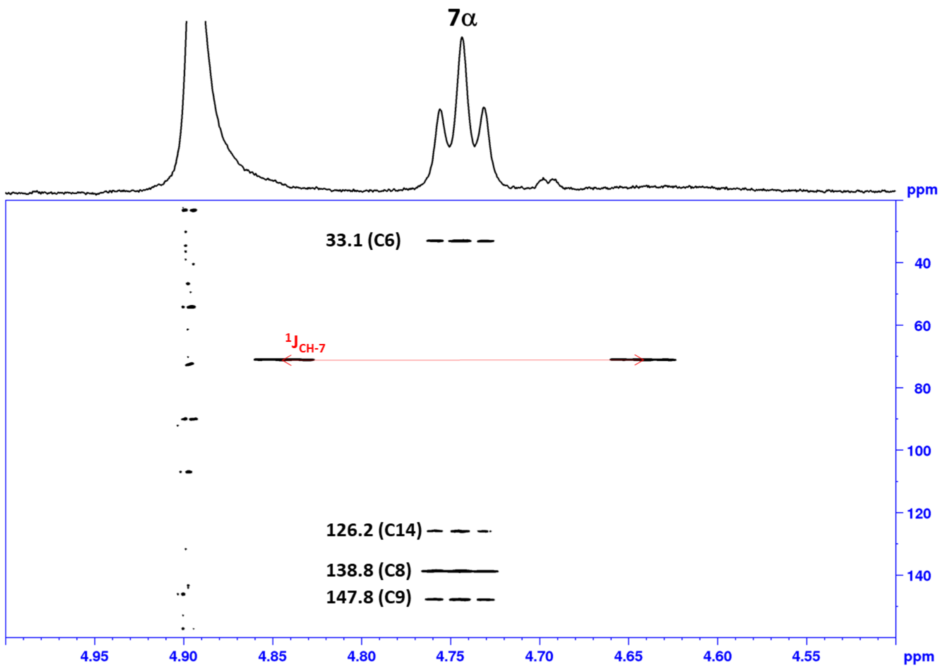

Supplemental Figure 30. Structure elucidation of **2**, ^{1}H-^{13}C HMBC spectrum, detail correlations from H-7α

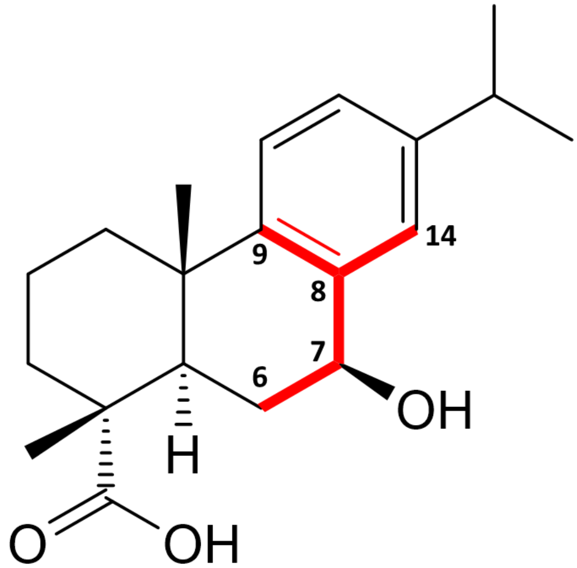

Supplemental Figure 31. Structure elucidation of **2**, structure of **2** with fragments elucidated from correlations of H-7α

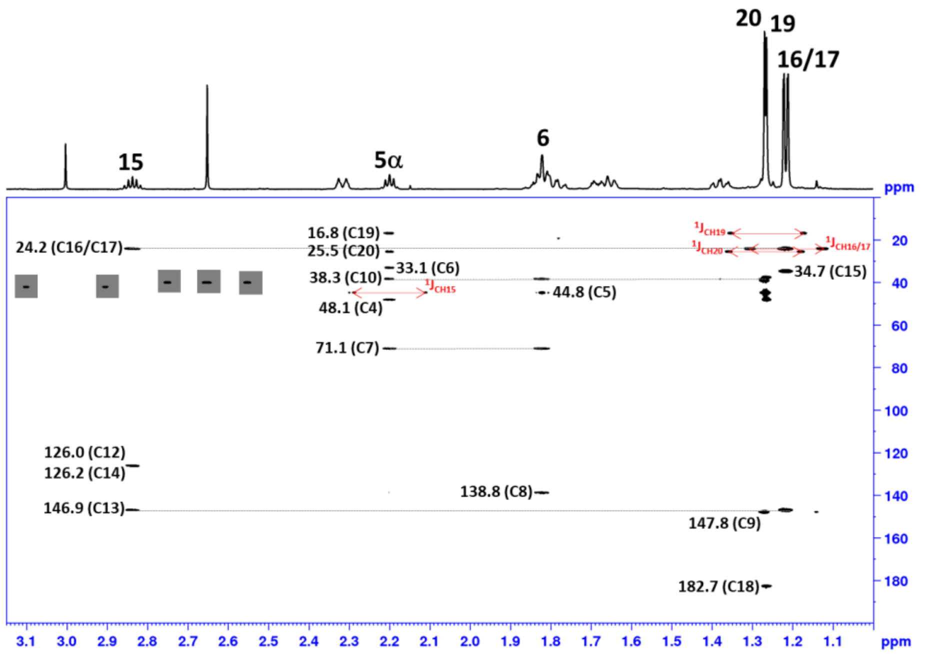

Supplemental Figure 32. Structure elucidation of **2**, ^{1}H-^{13}C HMBC spectrum, detail aliphatic range. Gray rectangles indicate impurities.

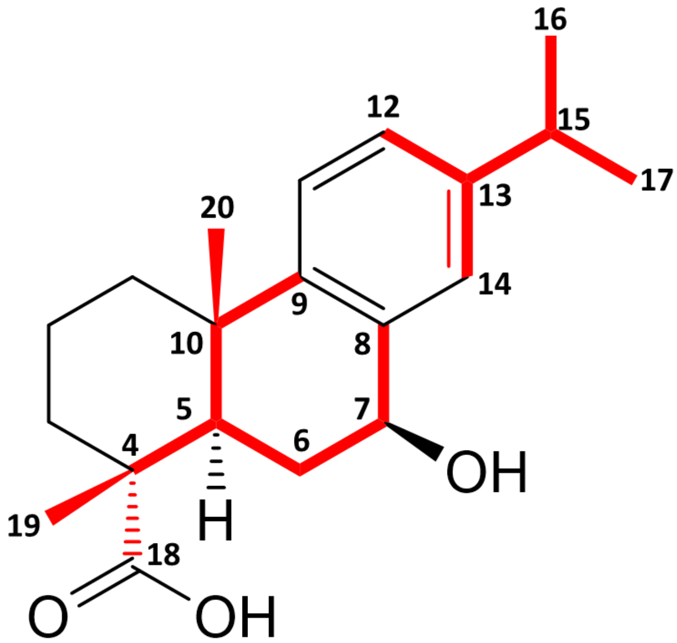

Supplemental Figure 33. Structure elucidation of **2**, structure of **2** with fragments elucidated from correlations in the aliphatic range

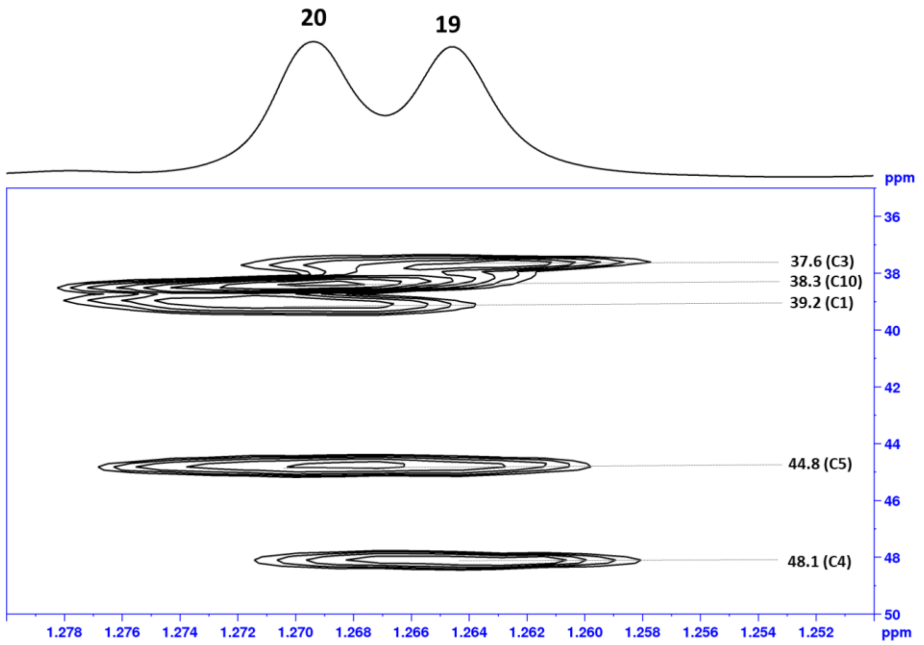

Supplemental Figure 34. Structure elucidation of **2**, ^{1}H-^{13}C HMBC spectrum, detail methyl range

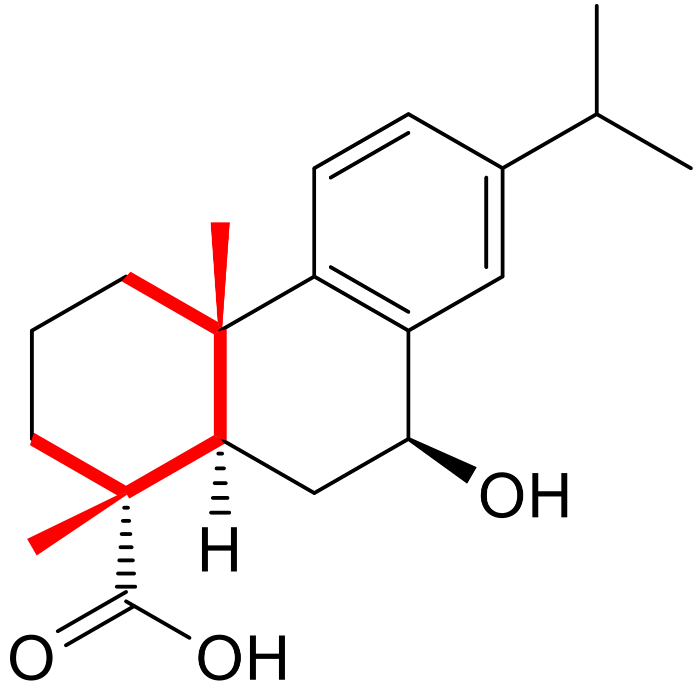

Supplemental Figure 35. Structure elucidation of **2**, structure of **2** with fragments elucidated from correlations in the methyl range

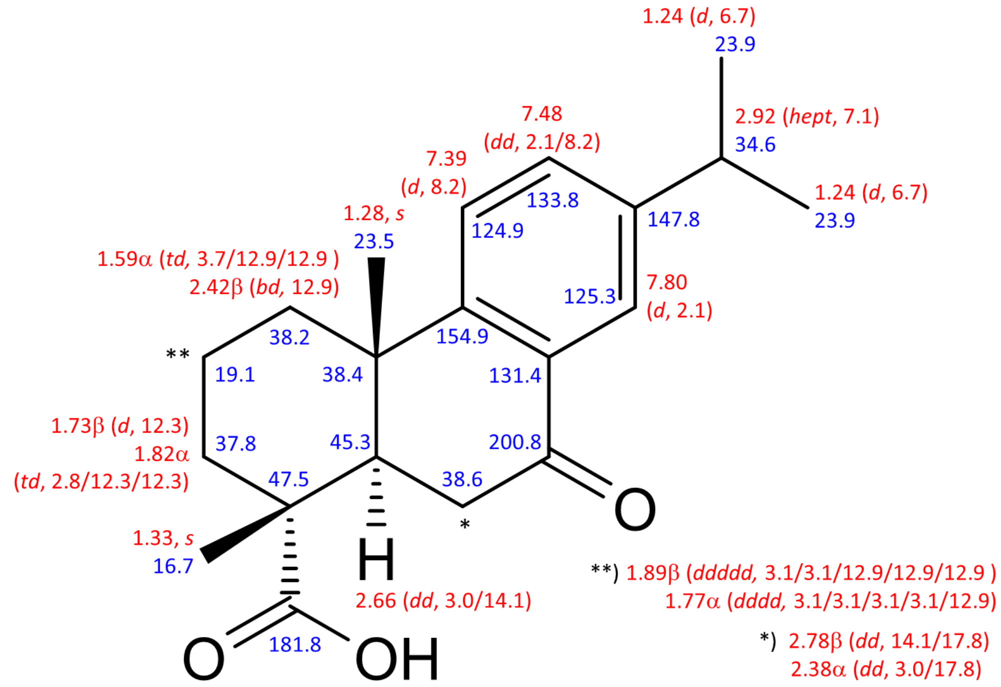

Supplemental Figure 36. Structure elucidation of **3**, overview

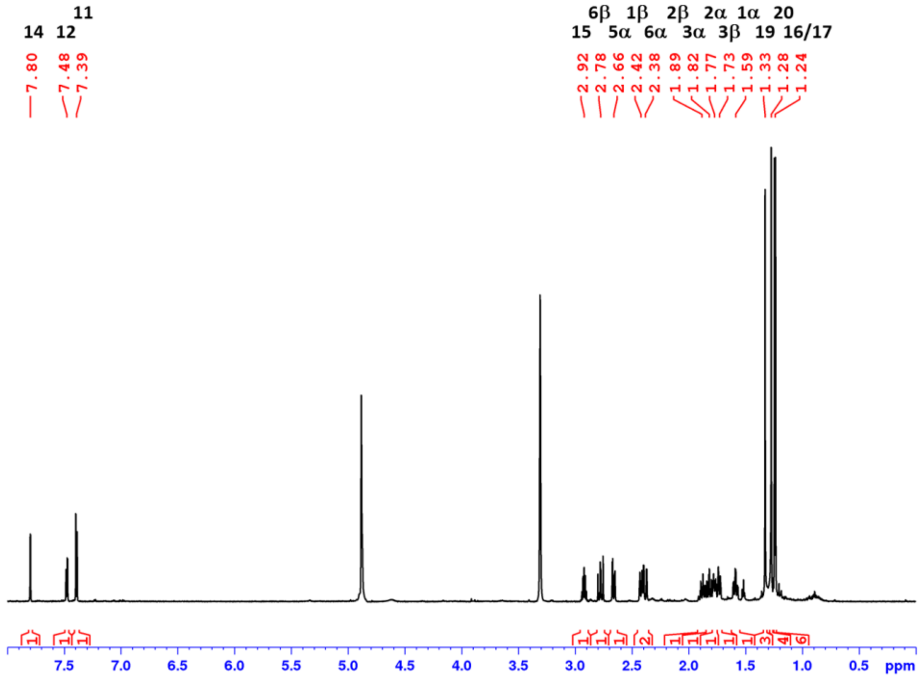

Supplemental Figure 37. Structure elucidation of **3**, ^{1}H-NMR spectrum, full range

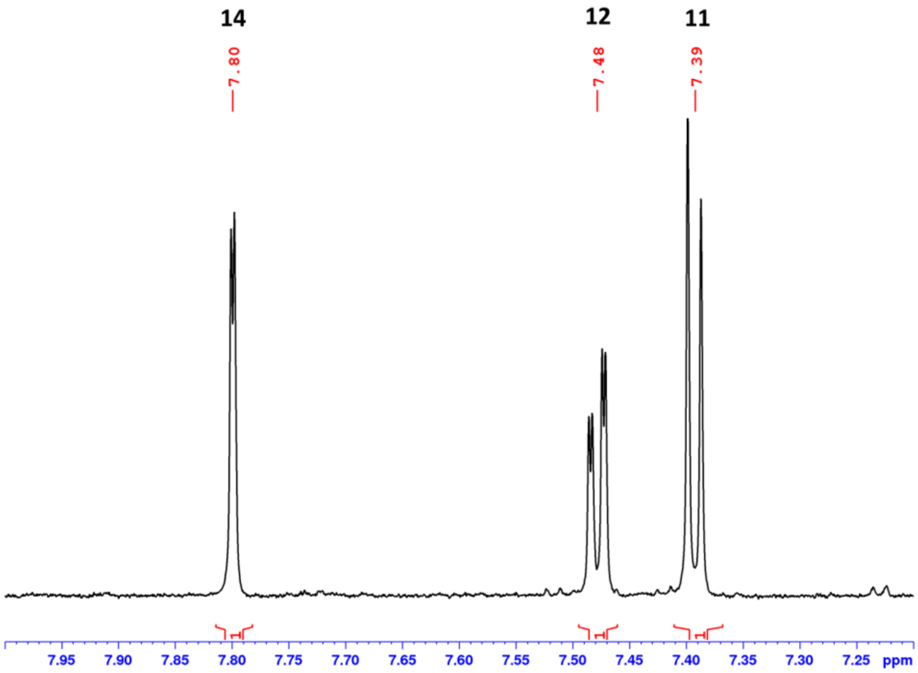

Supplemental Figure 38. Structure elucidation of **3**, ^{1}H-NMR spectrum, detail aromatic range

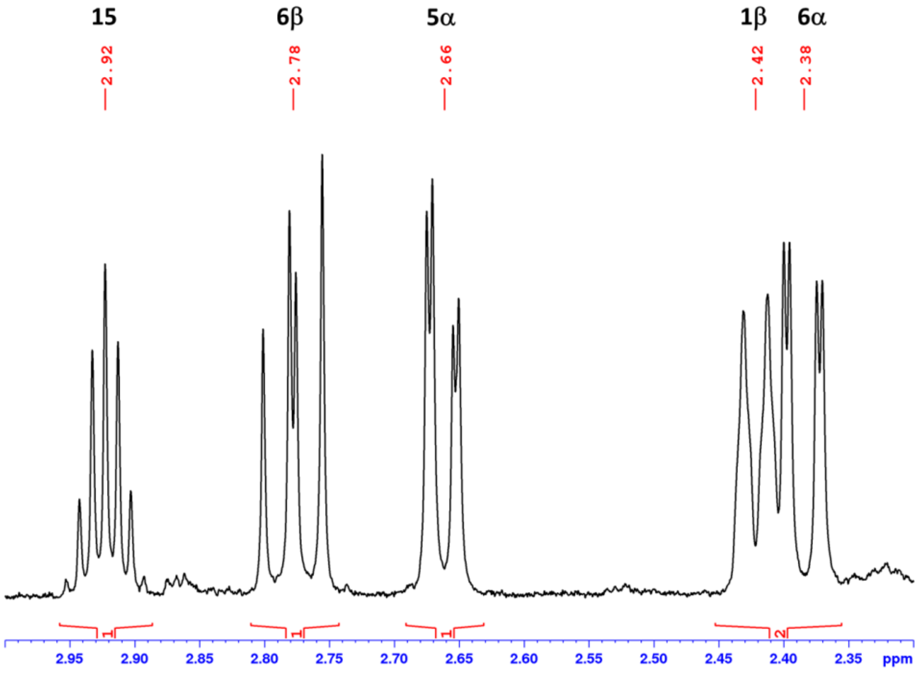

Supplemental Figure 39. Structure elucidation of **3**, ^{1}H-NMR spectrum, detail low field aliphatic range

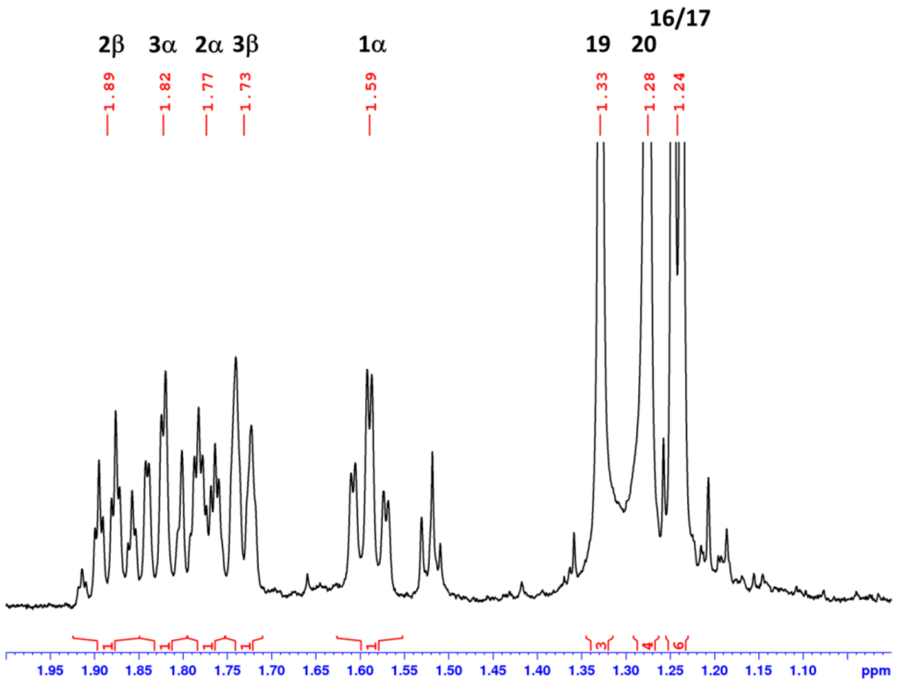

Supplemental Figure 40. Structure elucidation of **3**, ^{1}H-NMR spectrum, detail high field aliphatic range

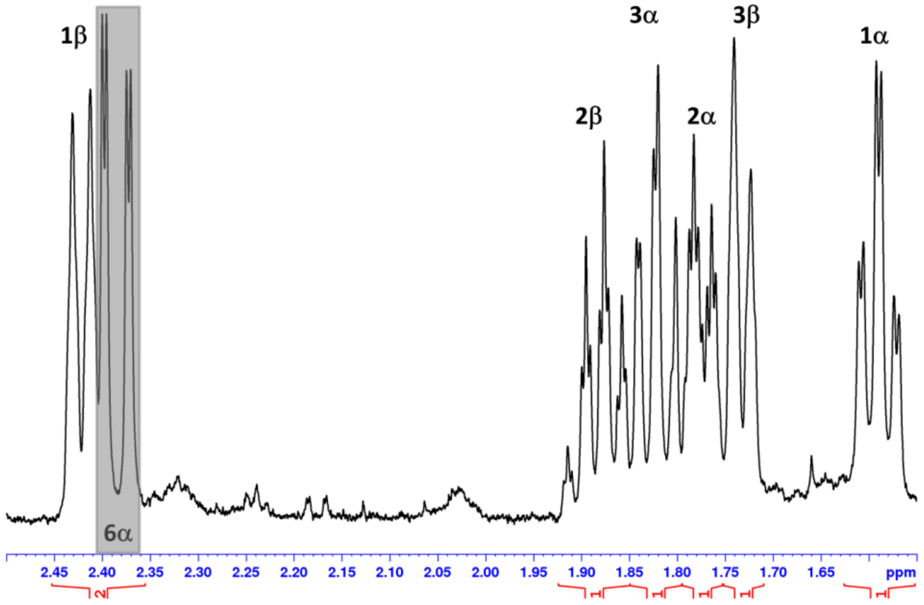

Supplemental Figure 41. Structure elucidation of **3**, comparison ^{1}H-NMR spectrum, detail positions 1 to 3

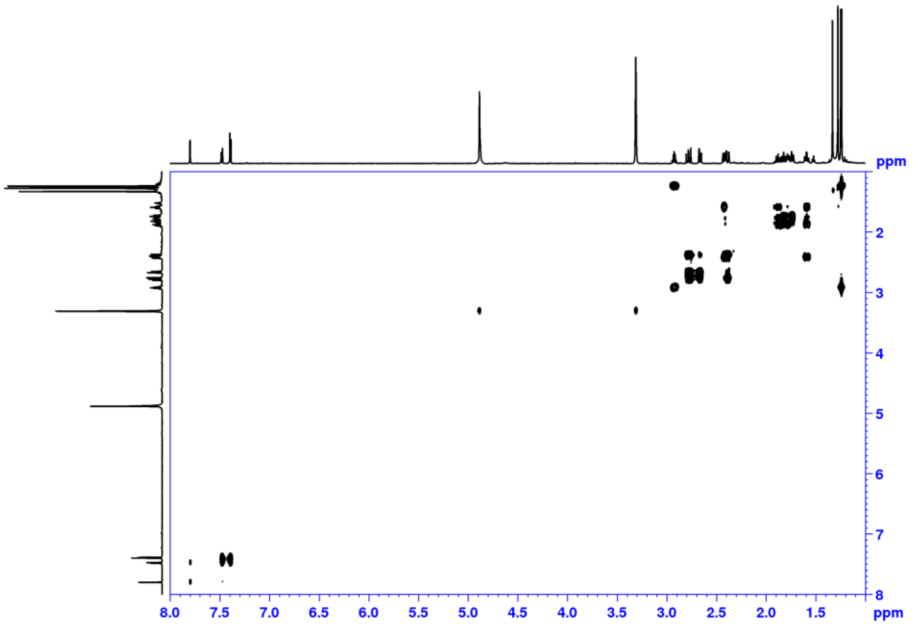

Supplemental Figure 42. Structure elucidation of **3**, ^{1}H-^{1}H COSY spectrum, full range

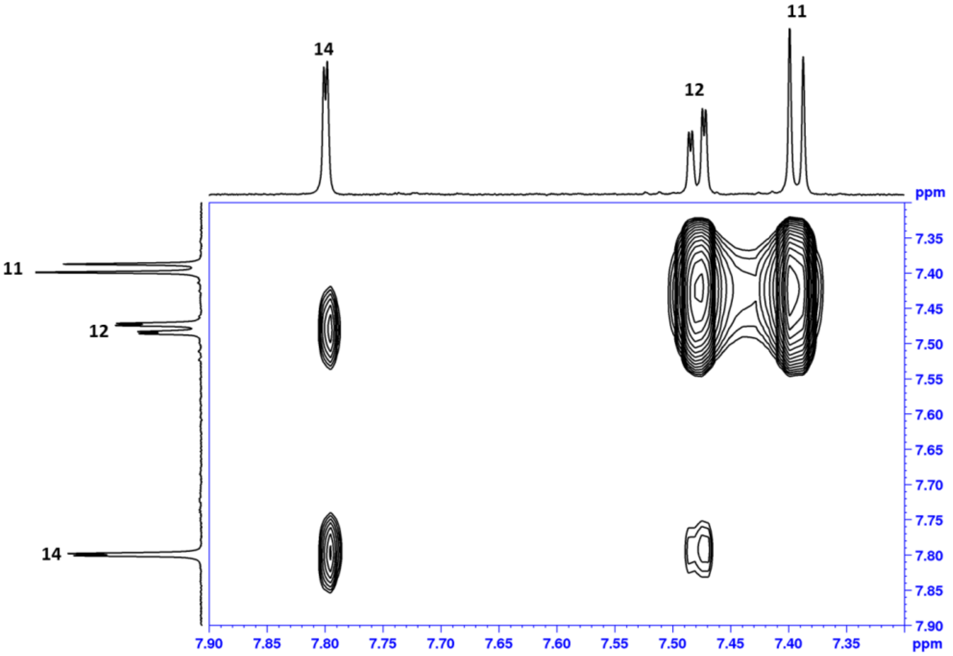

Supplemental Figure 43. Structure elucidation of **3**, ^{1}H-^{1}H COSY spectrum, detail aromatic range

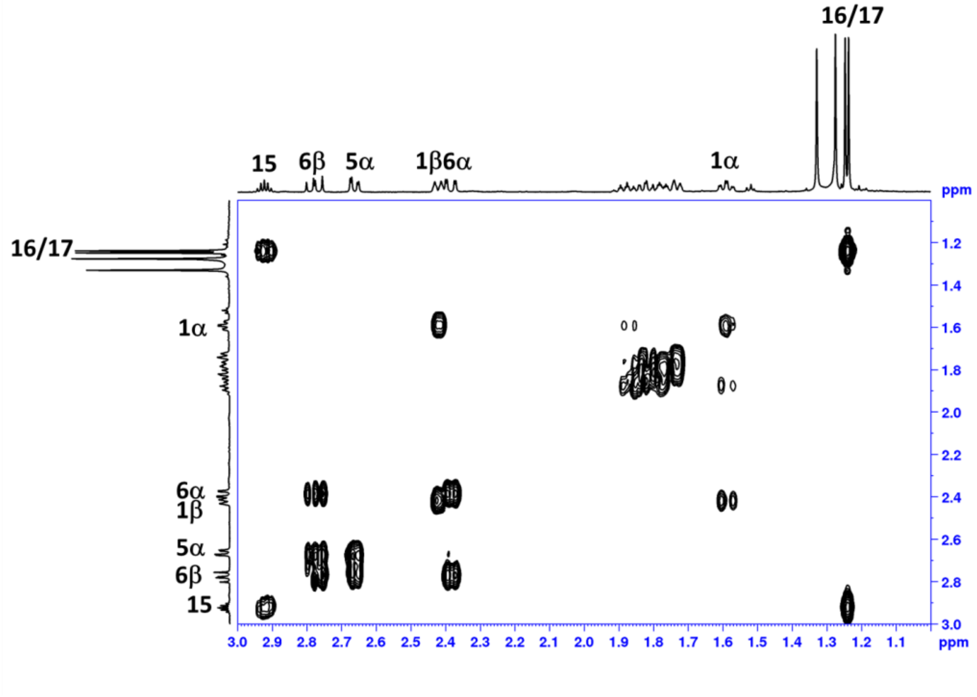

Supplemental Figure 44. Structure elucidation of **3**, ^{1}H-^{1}H COSY spectrum, detail aliphatic range

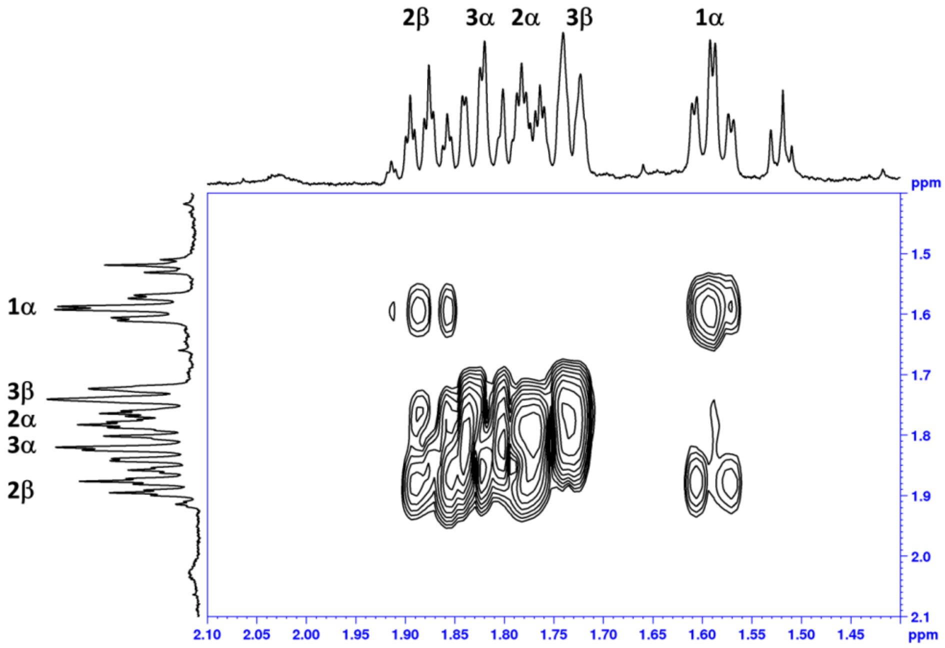

Supplemental Figure 45. Structure elucidation of **3**, ^{1}H-^{1}H COSY spectrum, detail aliphatic range, positions 1 to 3

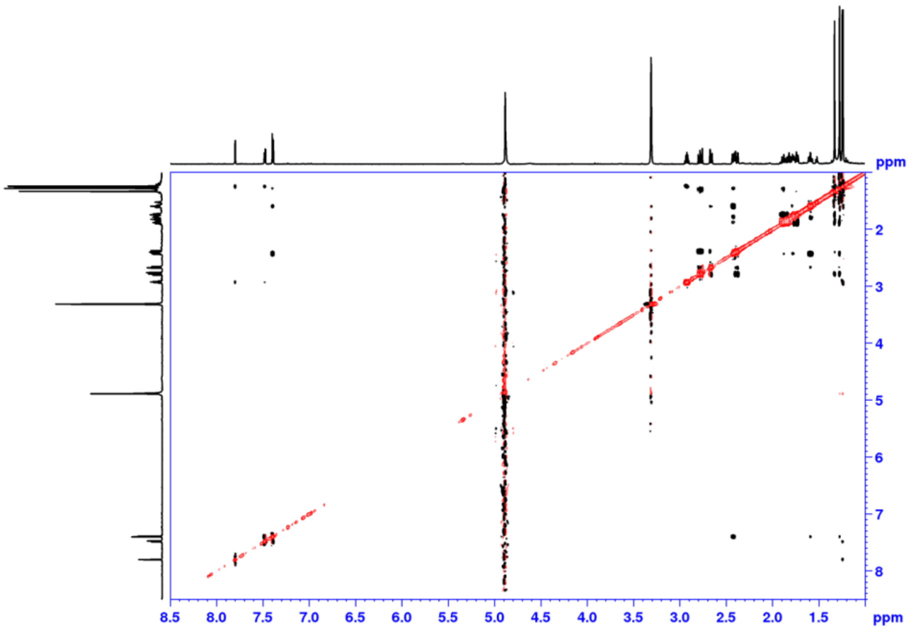

Supplemental Figure 46. Structure elucidation of **3**, ^{1}H-^{1}H ROESY spectrum, full range

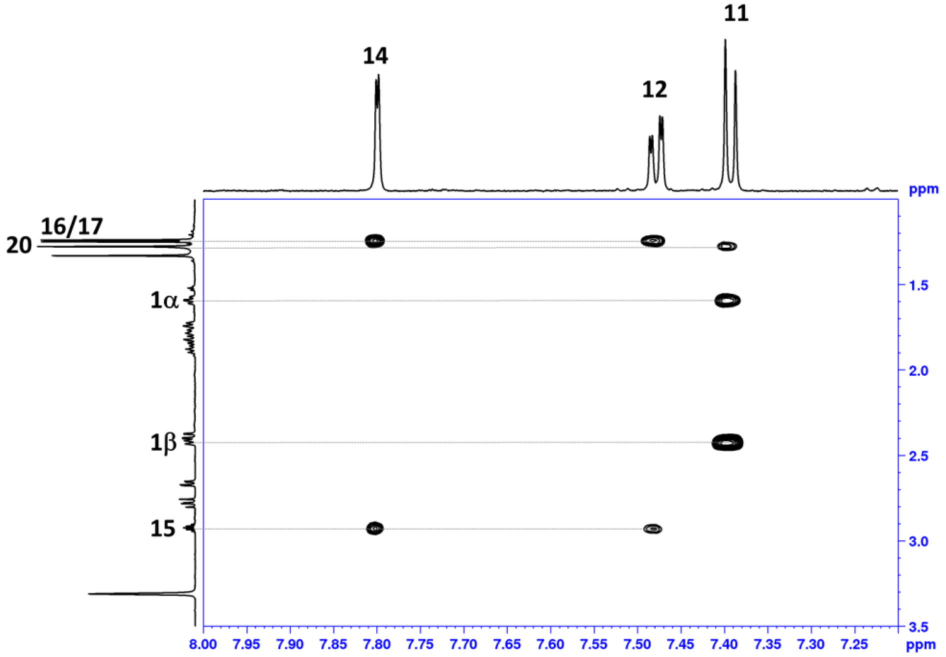

Supplemental Figure 47. Structure elucidation of **3**, ^{1}H-^{1}H ROESY spectrum, detail aromatic range

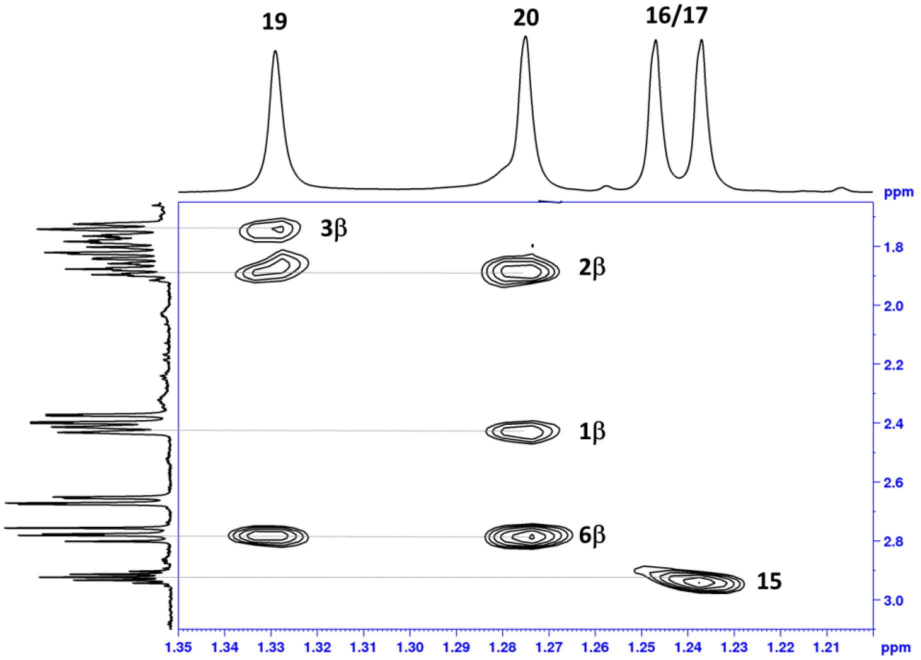

Supplemental Figure 48. Structure elucidation of **3**, ^{1}H-^{1}H ROESY spectrum, detail methyl correlations

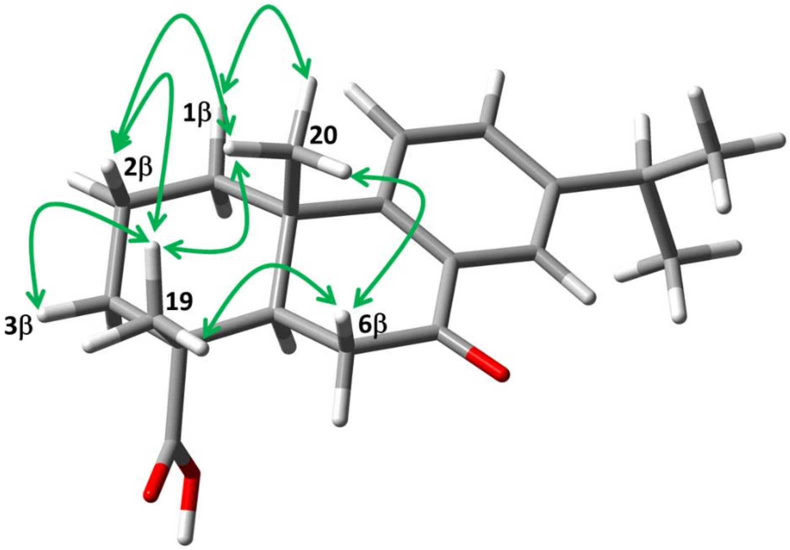

Supplemental Figure 49. Structure elucidation of **3**, structure of **3** with important ROESY correlations of β-oriented substituents

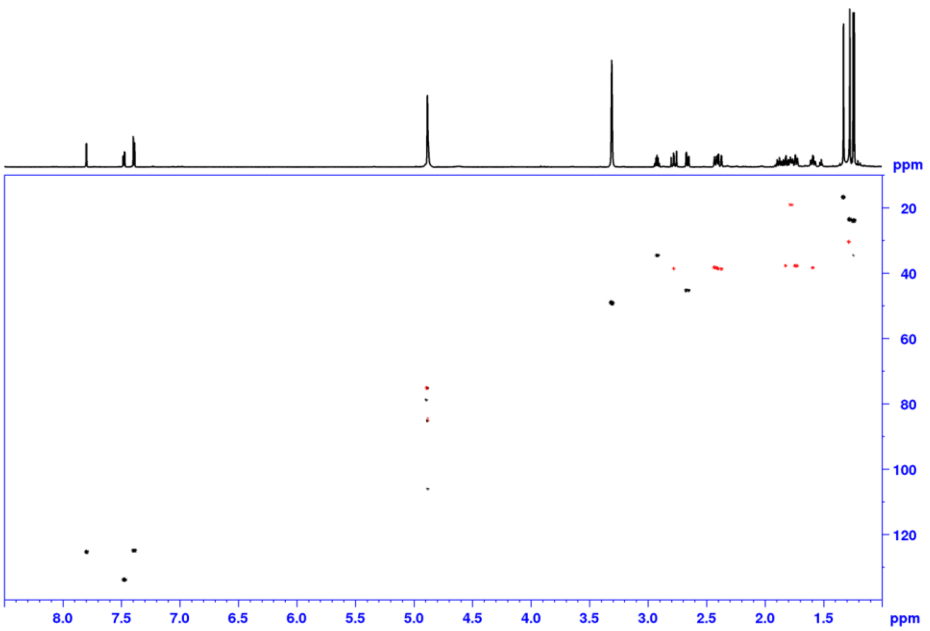

Supplemental Figure 50. Structure elucidation of **3**, ^{1}H-^{13}C HSQC spectrum, full range

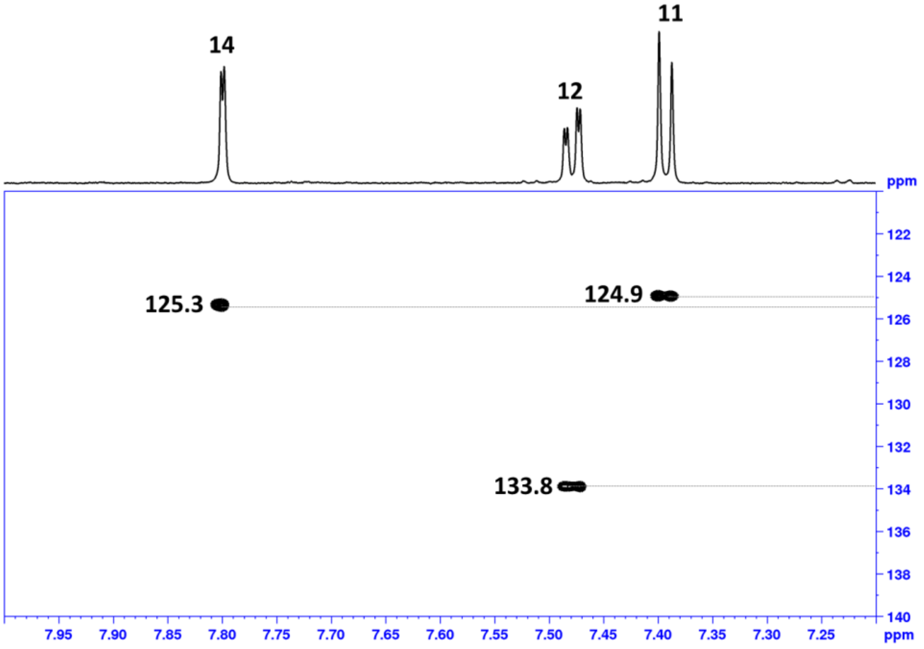

Supplemental Figure 51. Structure elucidation of **3**, ^{1}H-^{13}C HSQC spectrum, detail aromatic range

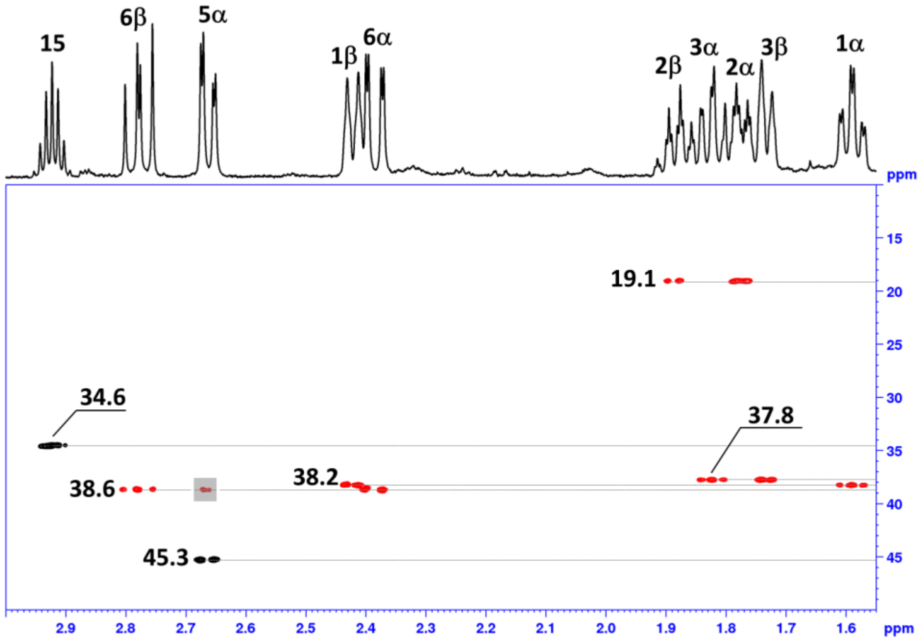

Supplemental Figure 52. Structure elucidation of **3**, ^{1}H-^{13}C HSQC spectrum, detail aliphatic range

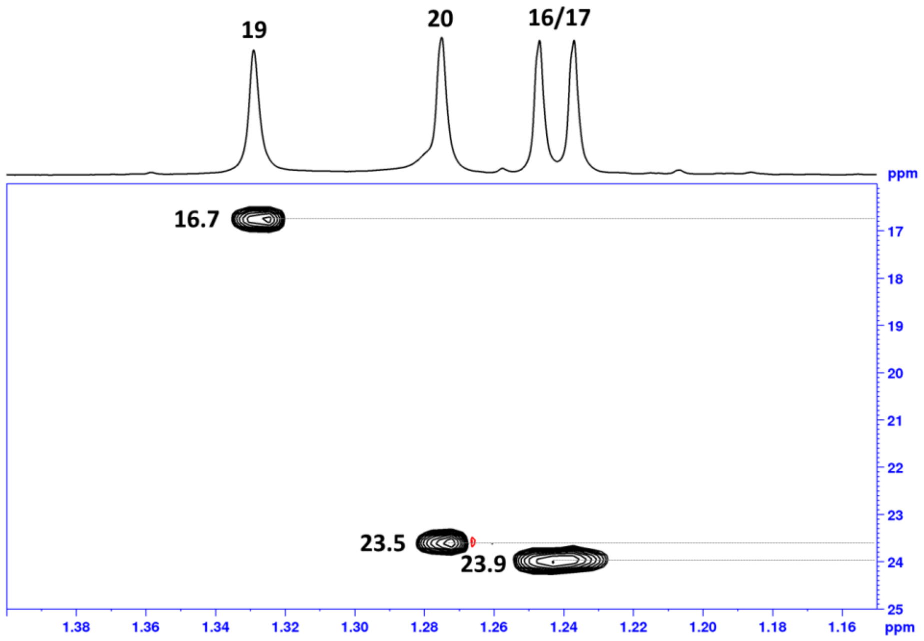

Supplemental Figure 53. Structure elucidation of **3**, ^{1}H-^{13}C HSQC spectrum, detail methyl range

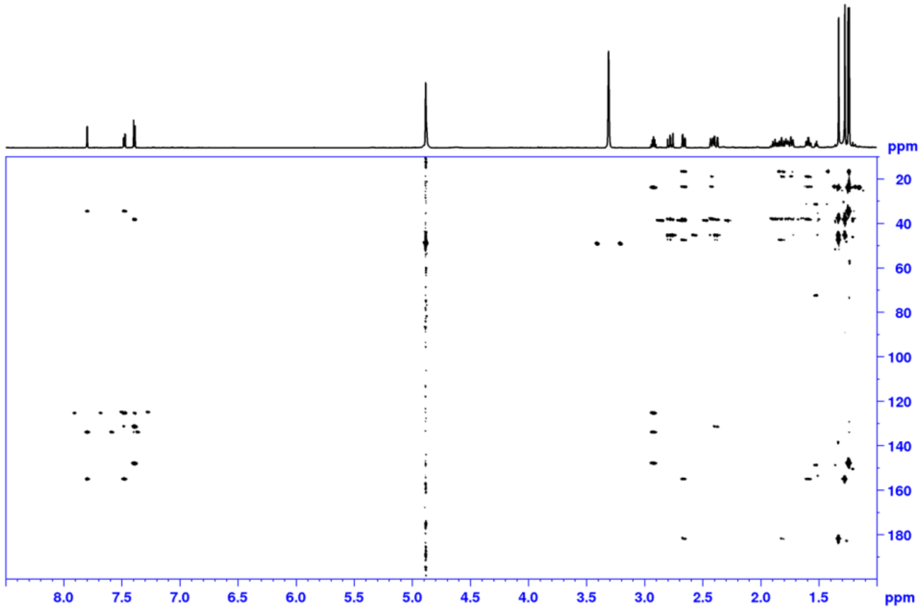

Supplemental Figure 54. Structure elucidation of **3**, ^{1}H-^{13}C HMBC spectrum, full range

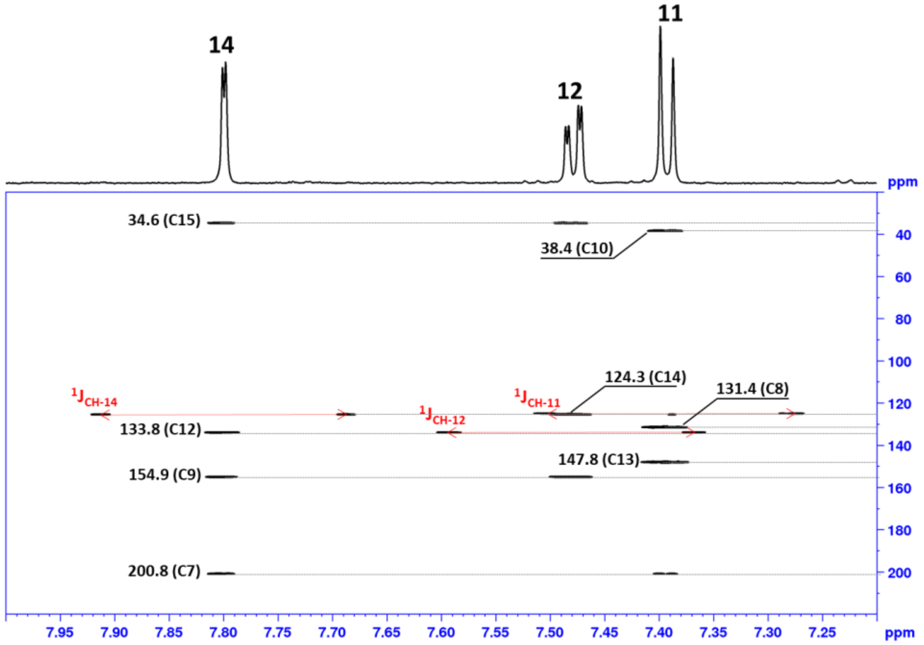

Supplemental Figure 55. Structure elucidation of **3**, ^{1}H-^{13}C HMBC spectrum, detail aromatic range

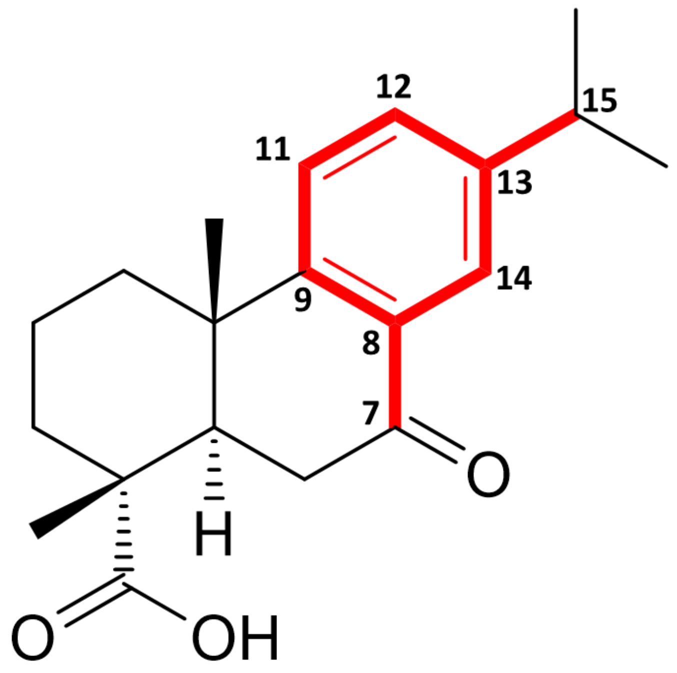

Supplemental Figure 56. Structure elucidation of **3**, structure of **3** with fragments elucidated from ^{1}H-^{13}C HMBC correlations in the aromatic range

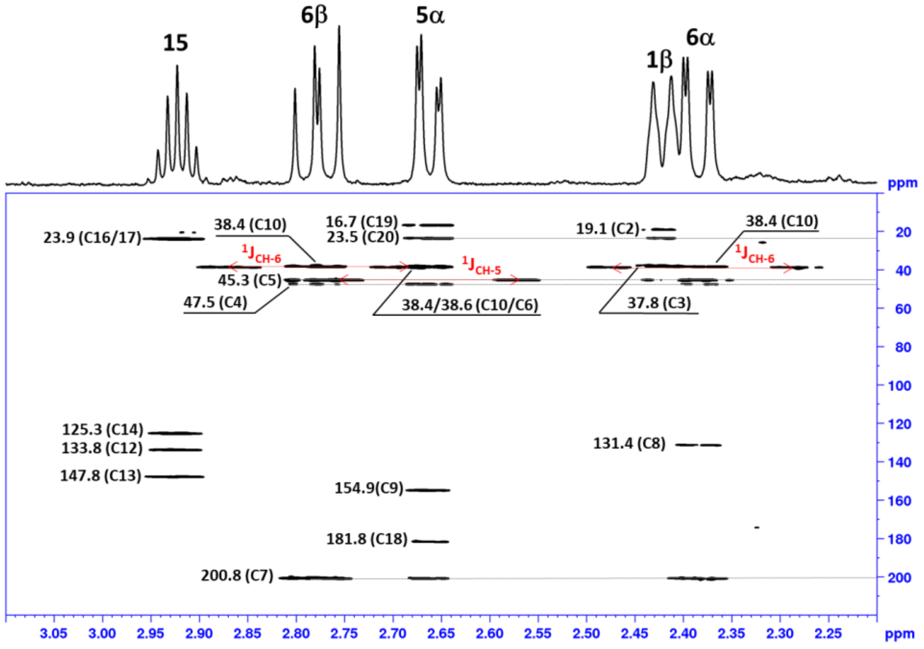

Supplemental Figure 57. Structure elucidation of **3**, ^{1}H-^{13}C HMBC spectrum, detail low field aliphatic range

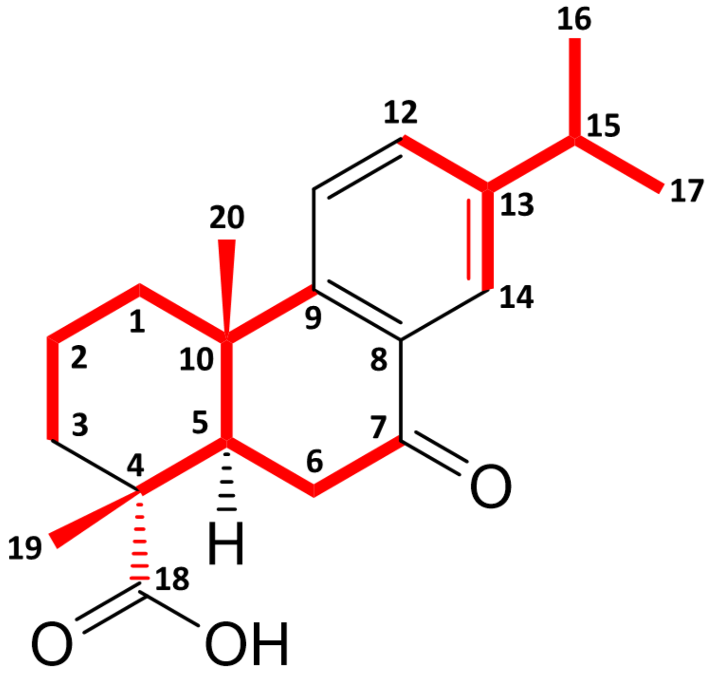

Supplemental Figure 58. Structure elucidation of **3**, structure of **3** with fragments elucidated from ^{1}H-^{13}C HMBC correlations in the low field aliphatic range

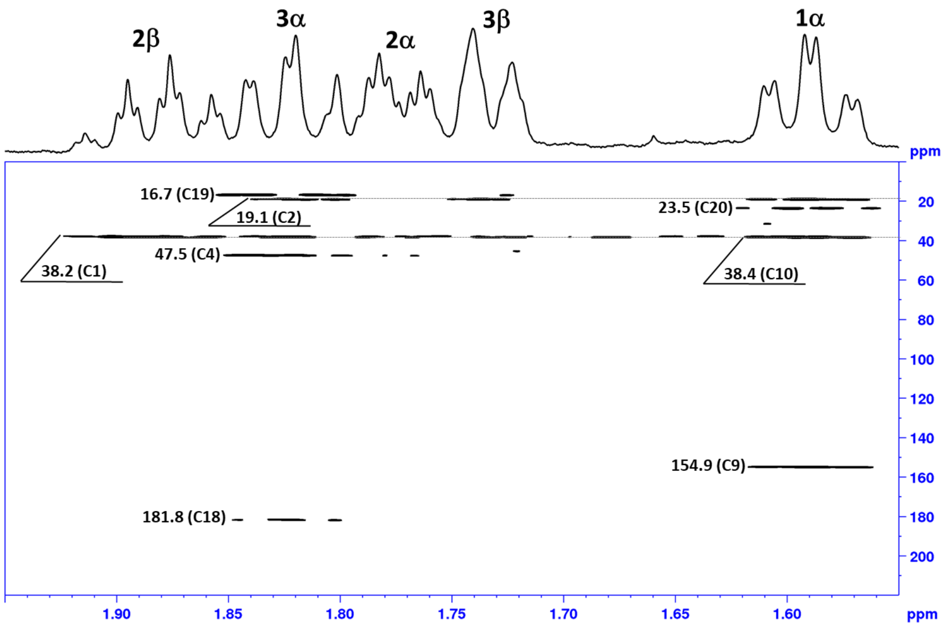

Supplemental Figure 59. Structure elucidation of **3**, ^{1}H-^{13}C HMBC spectrum, detail resonances ring A

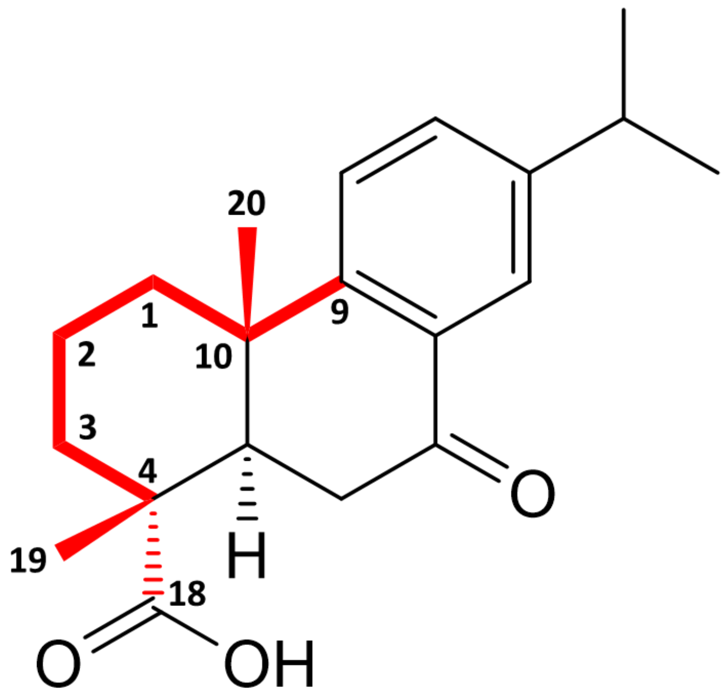

Supplemental Figure 60. Structure elucidation of **3**, structure of **3** with fragments elucidated from ^{1}H-^{13}C HMBC correlations of resonances in ring A

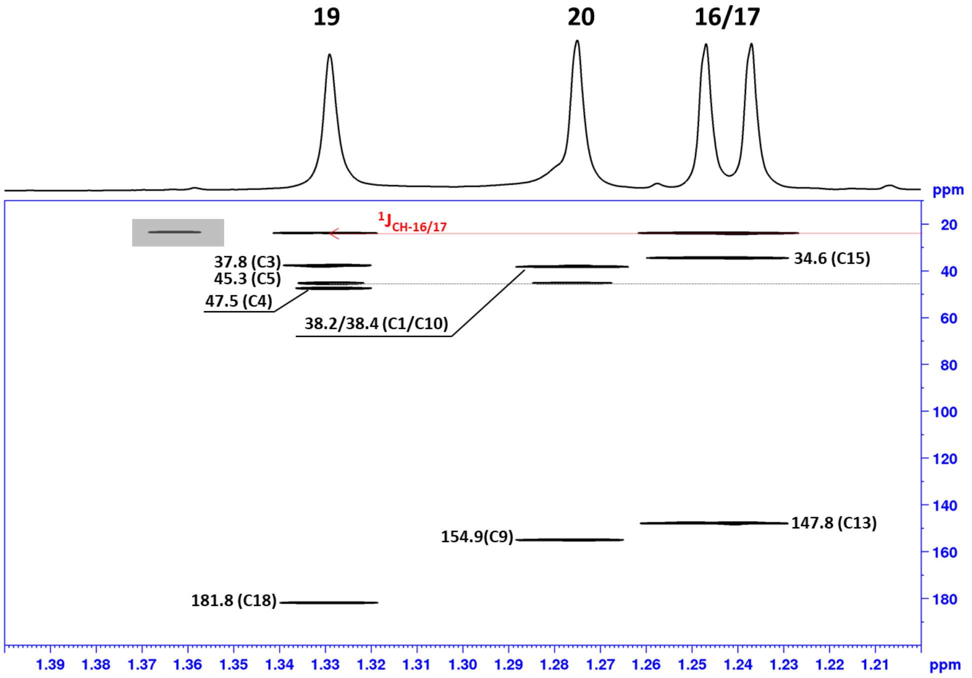

Supplemental Figure 61. Structure elucidation of **3**, ^{1}H-^{13}C HMBC spectrum, detail methyl range

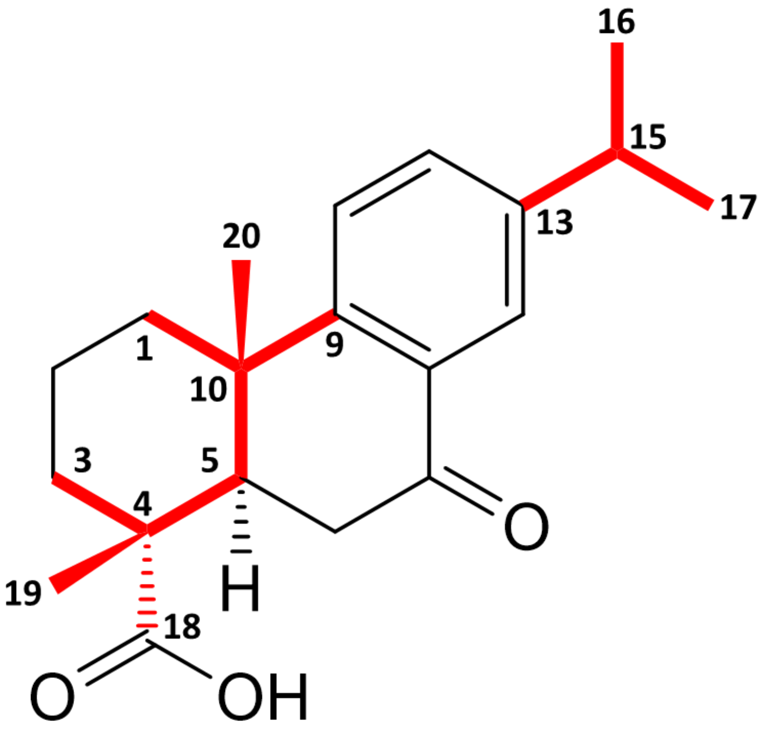

Supplemental Figure 62. Structure elucidation of **3**, structure of **3** with fragments elucidated from HMBC correlations in the methyl range

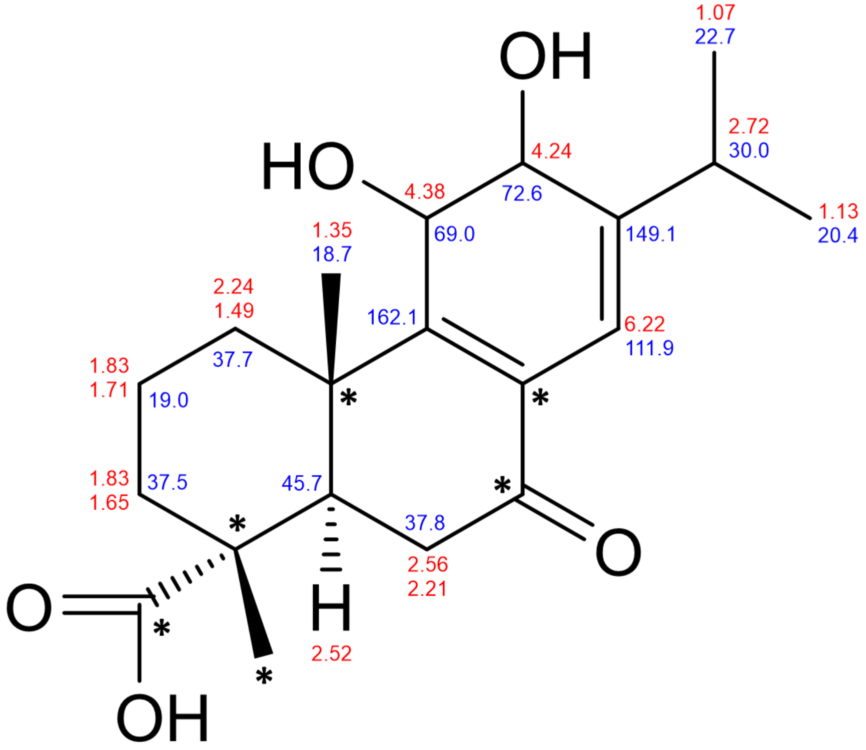

Supplemental Figure 63. Structure elucidation of **4**, overview

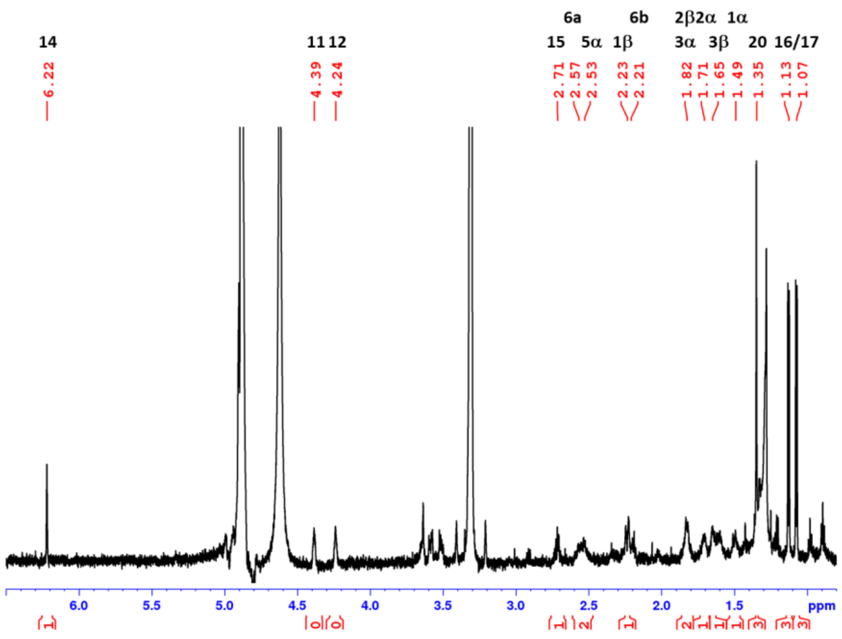

Supplemental Figure 64. Structure elucidation of **4**, ^{1}H-NMR spectrum, full range. The intensity of the signals for position 11 and 12 was decreased because of the applied water suppression.

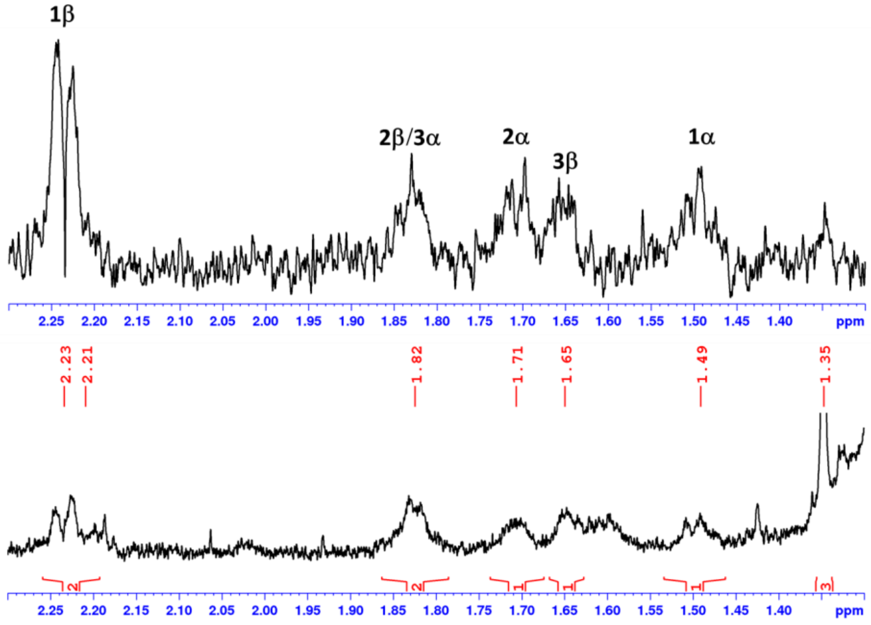

Supplemental Figure 65. Structure elucidation of **4**, ^{1}H-NMR spectrum, detail resonances ring A. The upper spectrum is a SELTOCY with the transmitter set on resonance with H-1β. The positions have been assigned based on signal shapes and chemical shifts determined for the other compounds described in this publication (Supplemental Table 1).

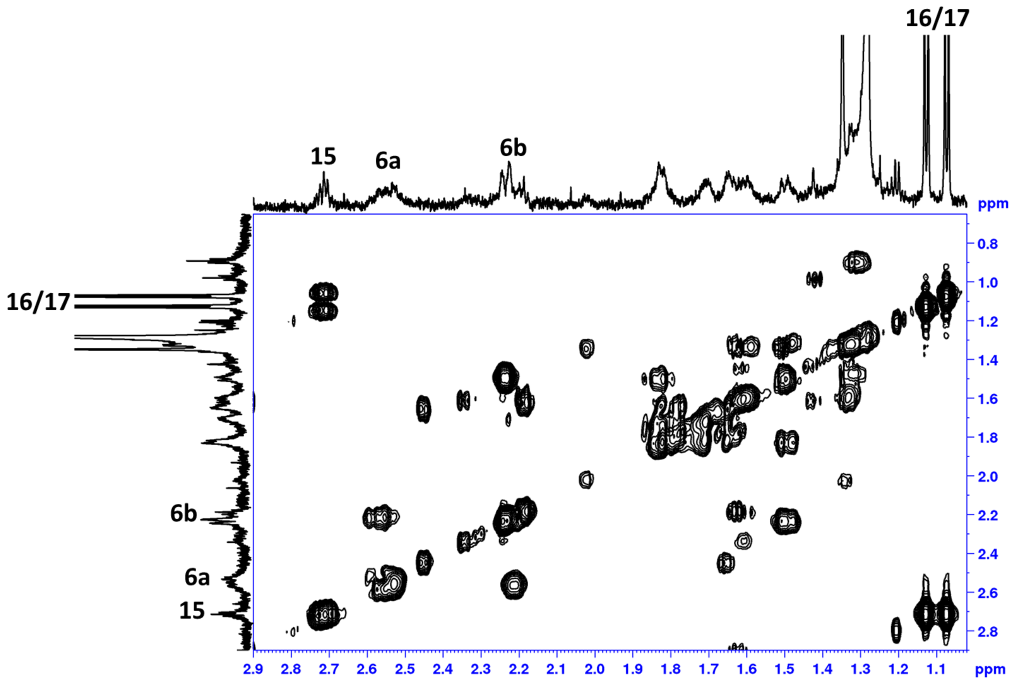

Supplemental Figure 66. Structure elucidation of **4**, ^{1}H-^{1}H COSY spectrum, detail aliphatic range

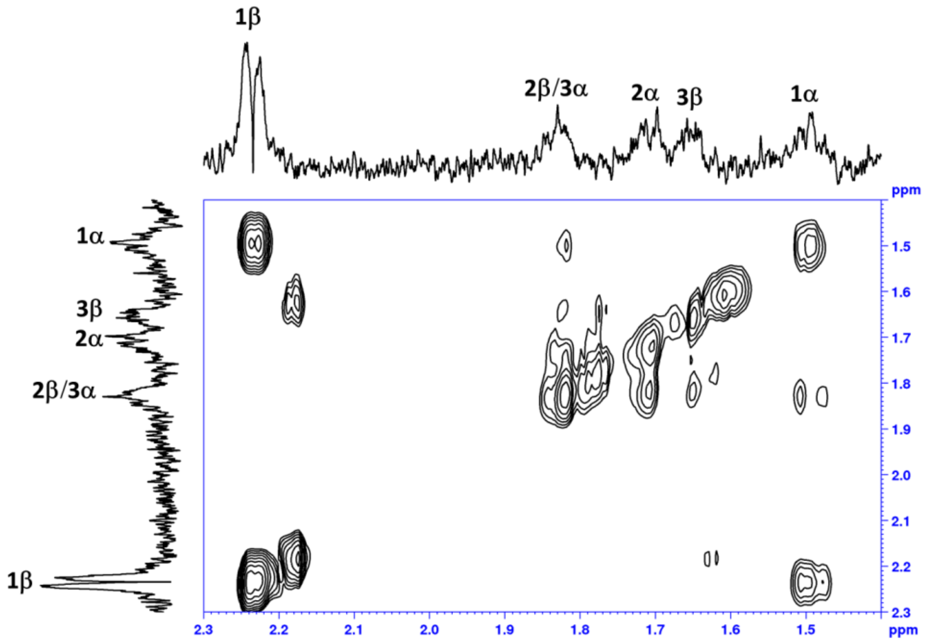

Supplemental Figure 67. Structure elucidation of **4**, ^{1}H-^{1}H COSY spectrum, detail aliphatic range, positions 1 to 3. A SELTOCY with the transmitter set on resonance with H-1β is used for the projections in F1 and F2.

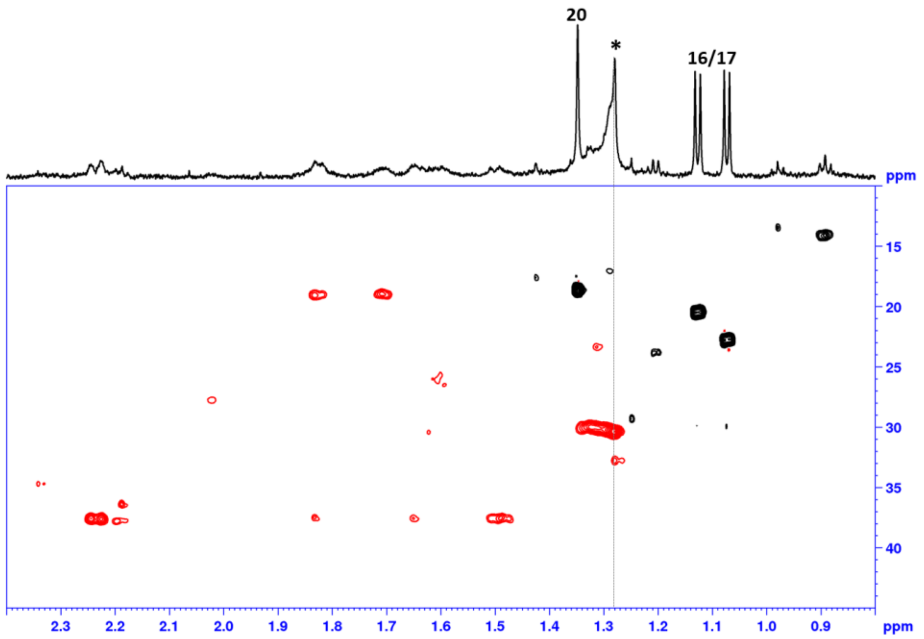

Supplemental Figure 68. Structure elucidation of **4**, ^{1}H-^{13}C HSQC spectrum, detail aliphatic range. An aliphatic impurity (*) appears exactly on the position for the methyl resonance CH_3-19, which leads to cancellation of the signal.

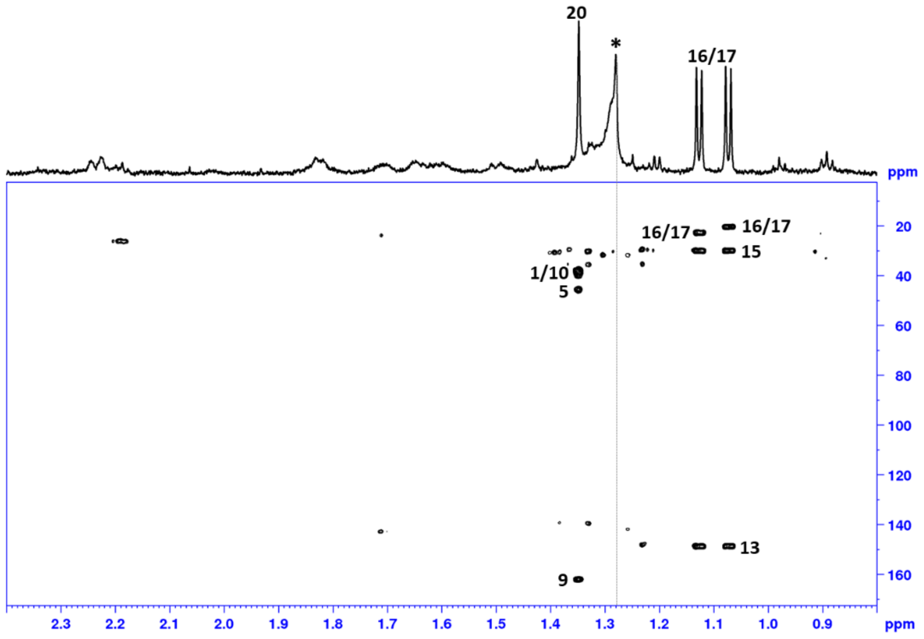

Supplemental Figure 69. Structure elucidation of **4**, ^{1}H-^{13}C HMBC spectrum, detail aliphatic range. An aliphatic impurity (*) appears exactly on the position for the methyl resonance CH_3-19, which leads to cancellation of the signal and its correlations.

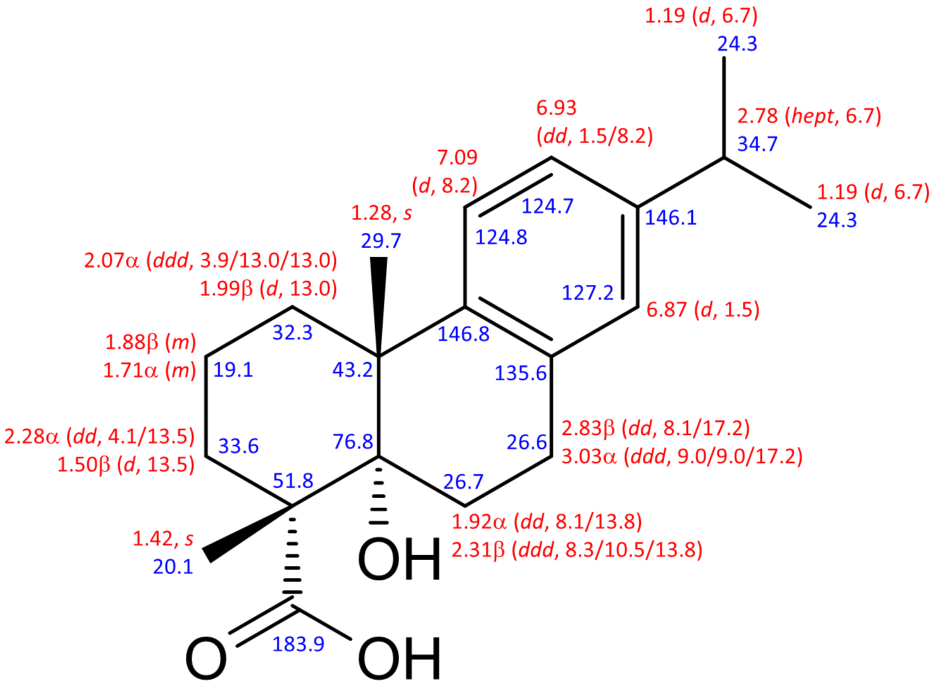

Supplemental Figure 70. Structure elucidation of **5**, overview

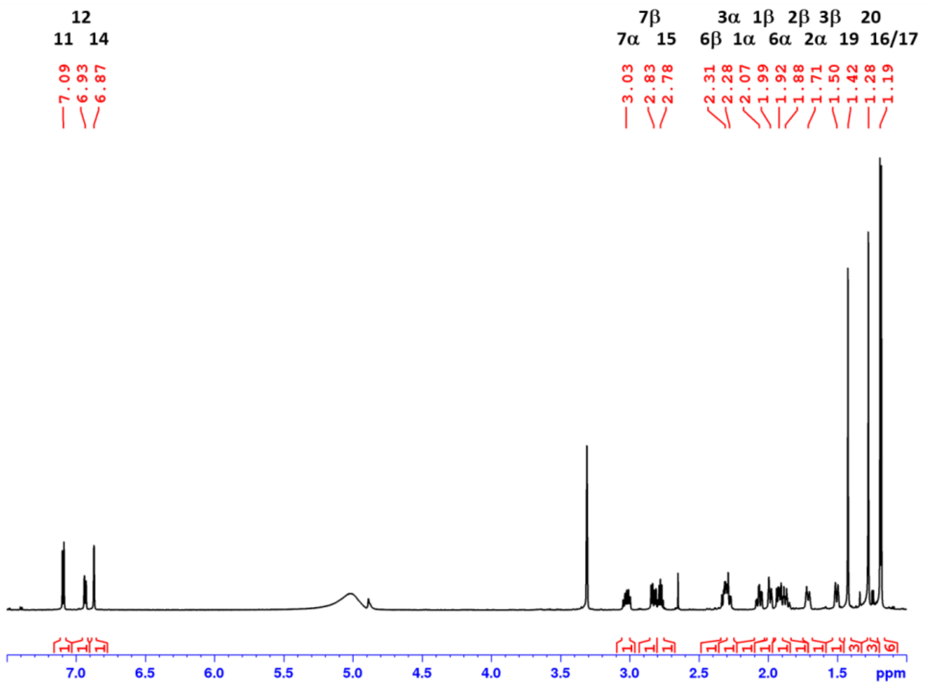

Supplemental Figure 71. Structure elucidation of **5**, ^{1}H-NMR spectrum, full range

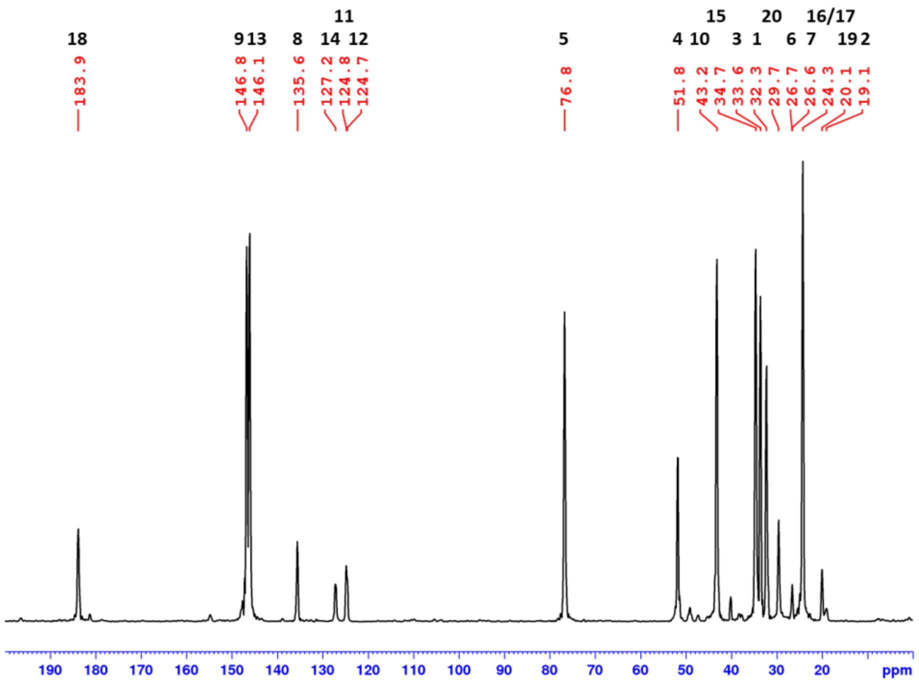

Supplemental Figure 72. Structure elucidation of **5**, ^{13}C-NMR data. The spectrum represents the combined projections from ^{1}H-^{13}C HSQC and ^{1}H-^{13}C HMBC correlations.

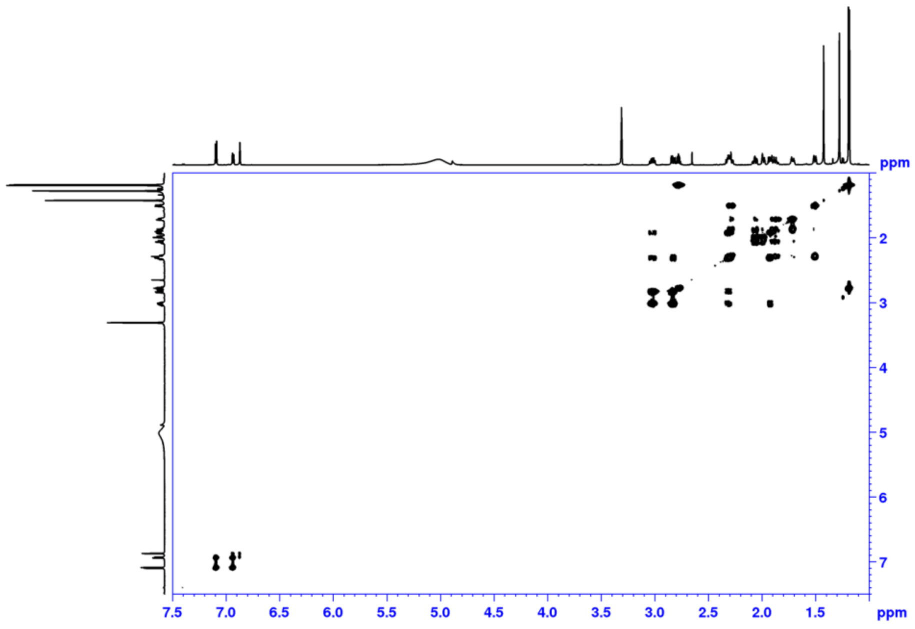

Supplemental Figure 73. Structure elucidation of **5**, ^{1}H-^{1}H COSY spectrum, full range

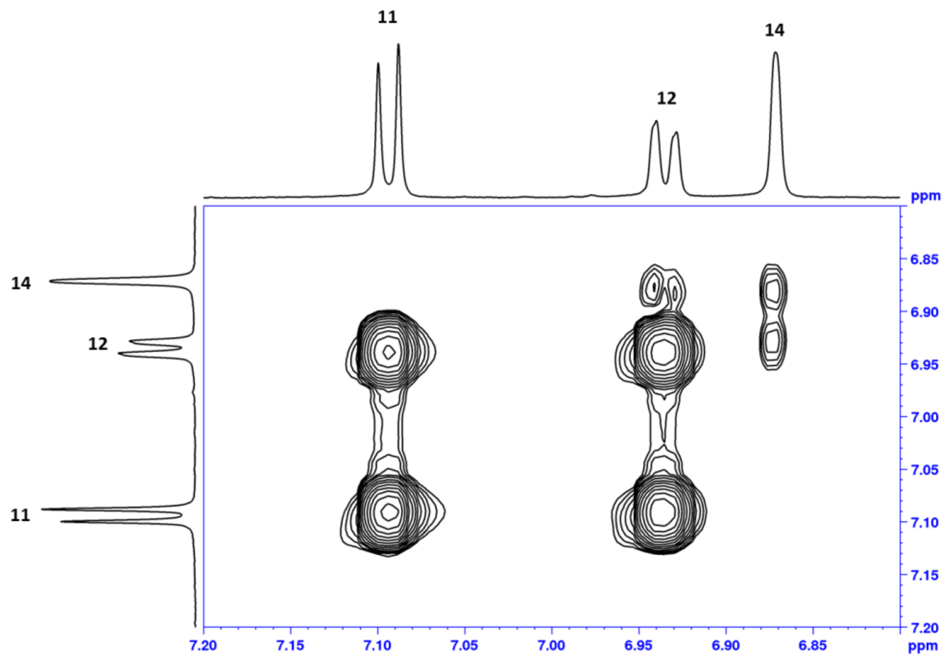

Supplemental Figure 74. Structure elucidation of **5**, ^1H-^1H COSY spectrum, detail aromatic range

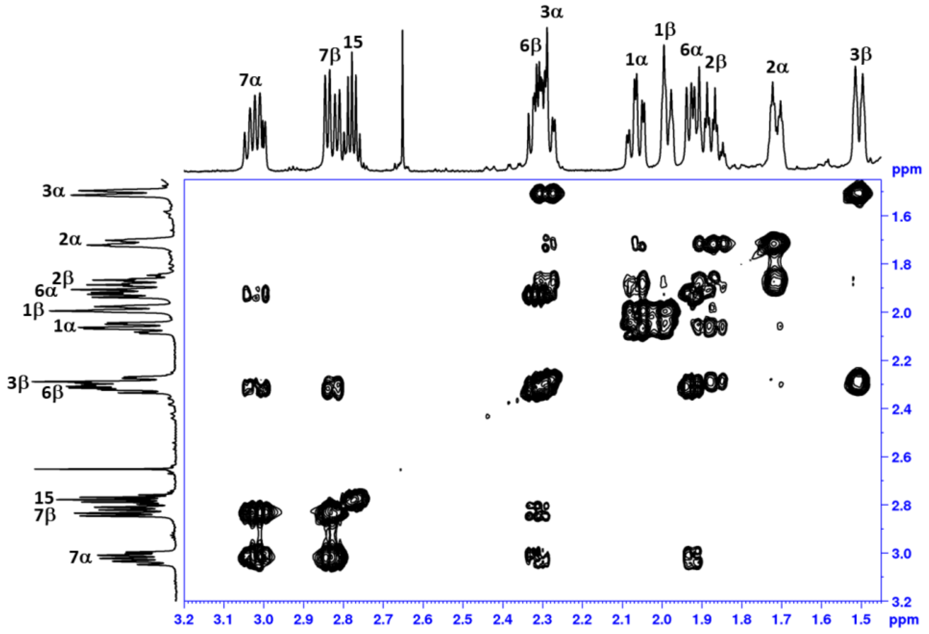

Supplemental Figure 75. Structure elucidation of **5**, ^1H-^1H COSY spectrum, detail aliphatic range

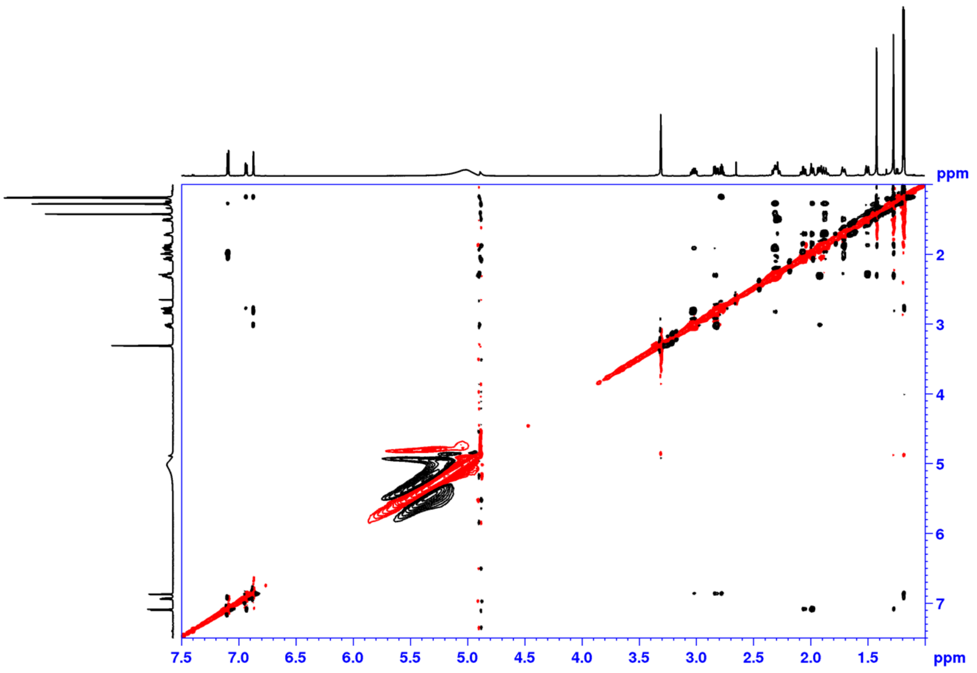

Supplemental Figure 76. Structure elucidation of **5**, ^{1}H-^{1}H ROESY spectrum, full range

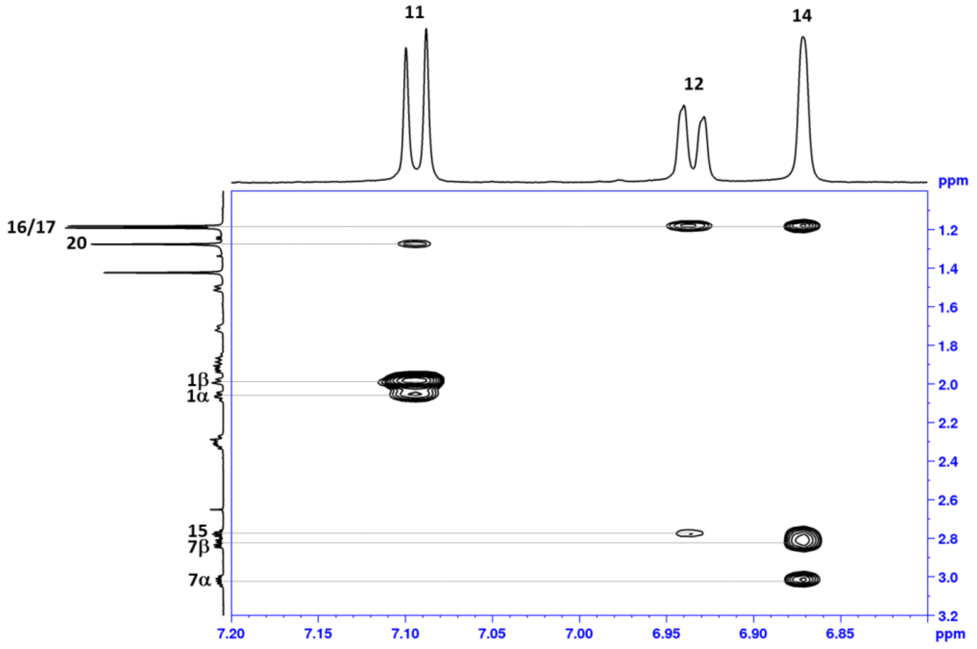

Supplemental Figure 77. Structure elucidation of **5**, ^{1}H-^{1}H ROESY spectrum, detail aromatic range

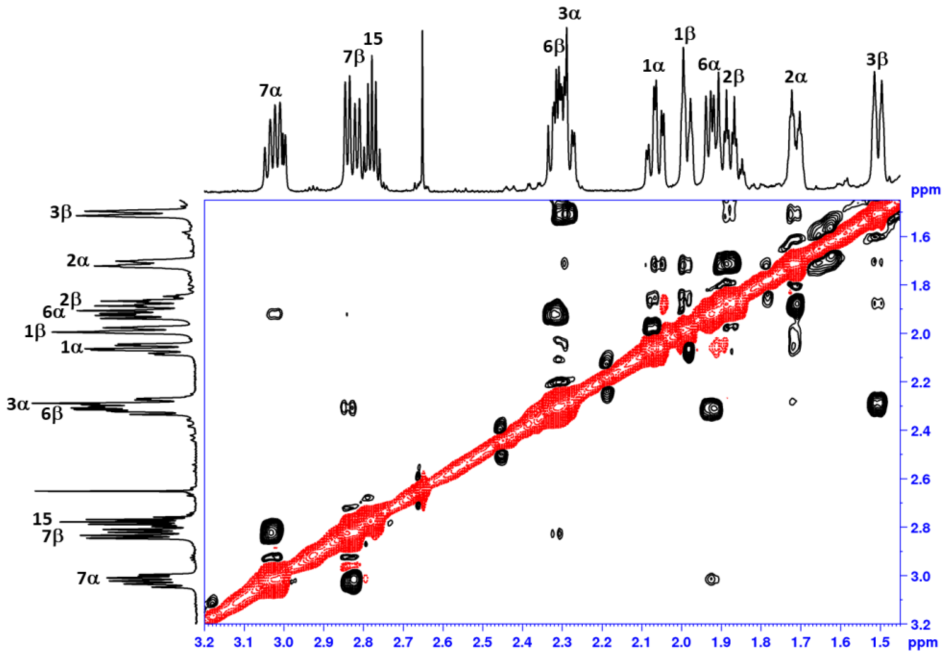

Supplemental Figure 78. Structure elucidation of **5**, ^{1}H-^{1}H ROESY spectrum, detail aliphatic range

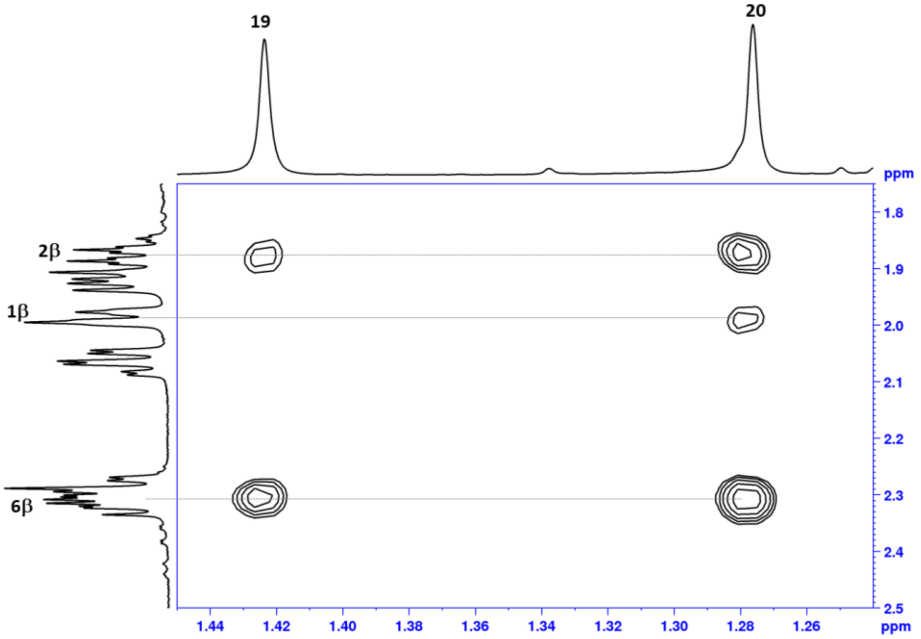

Supplemental Figure 79. Structure elucidation of **5**, ^{1}H-^{1}H ROESY spectrum, detail methyl correlations

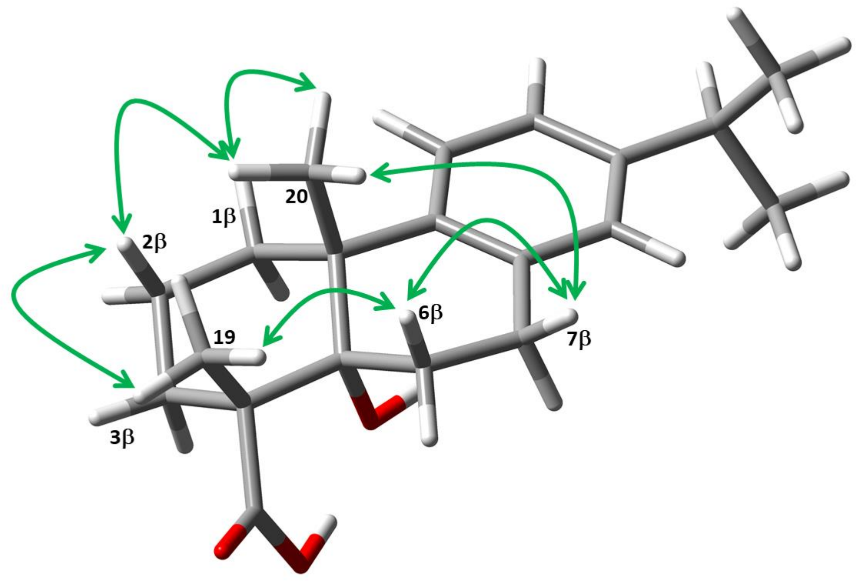

Supplemental Figure 80. Structure elucidation of **5**, structure of **5** with important ROESY correlations of β-oriented substituents

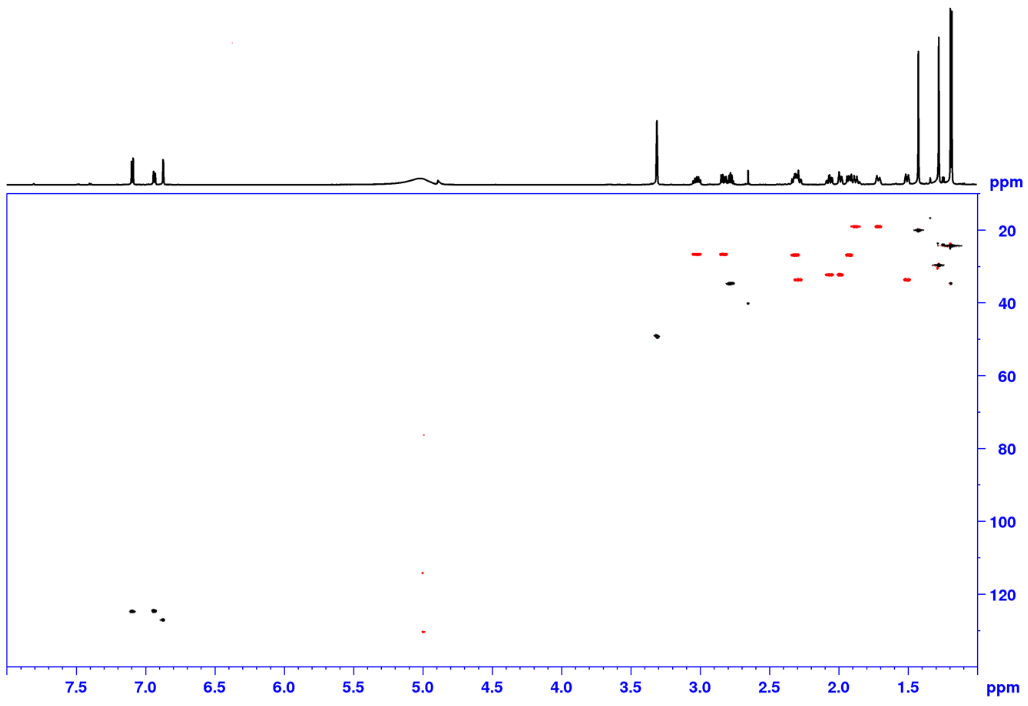

Supplemental Figure 81. Structure elucidation of **5**, ^{1}H-^{13}C HSQC spectrum, full range

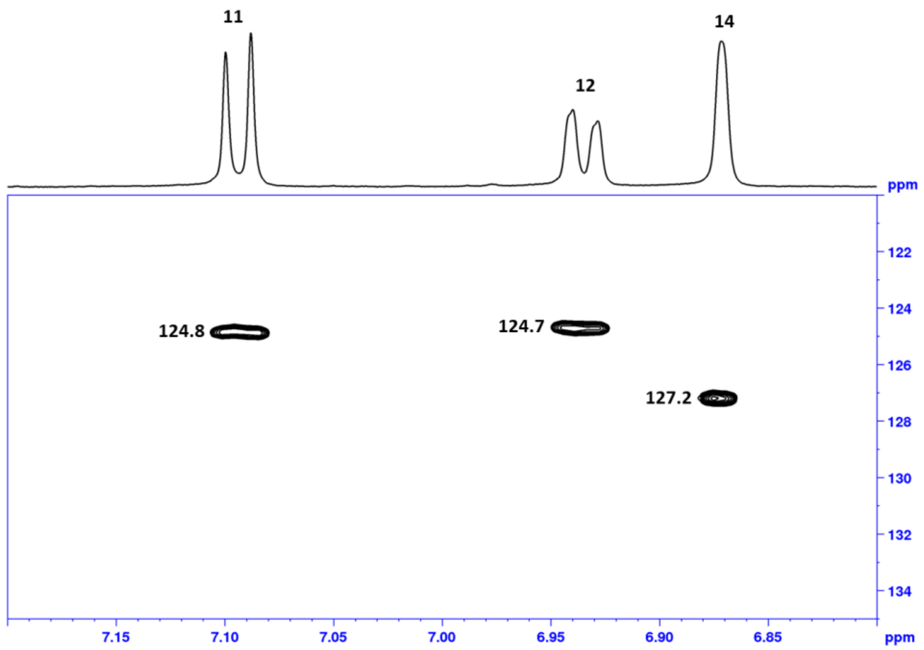

Supplemental Figure 82. Structure elucidation of **5**, ^{1}H-^{13}C HSQC spectrum, detail aromatic range

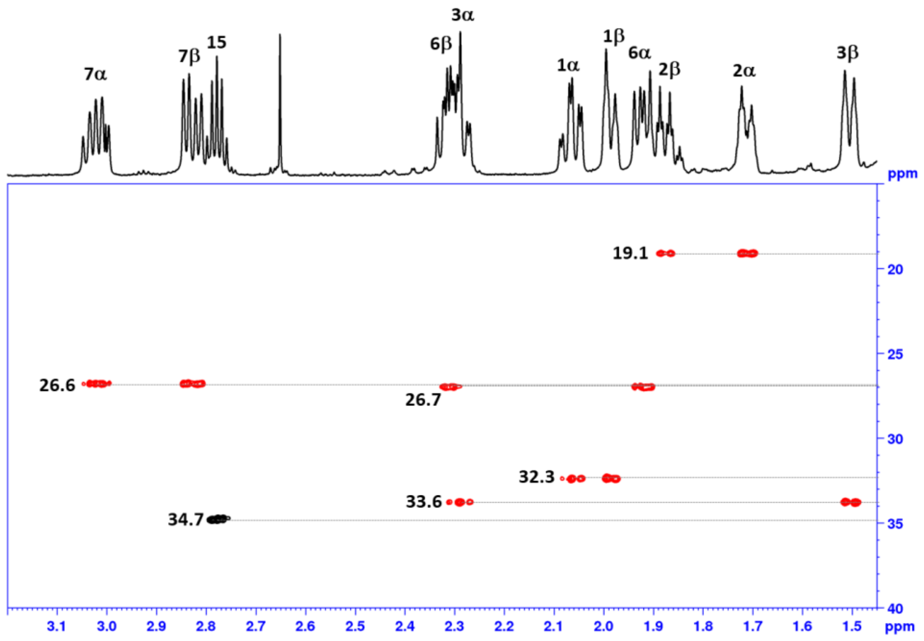

Supplemental Figure 83. Structure elucidation of **5**, ^{1}H-^{13}C HSQC spectrum, detail aliphatic range

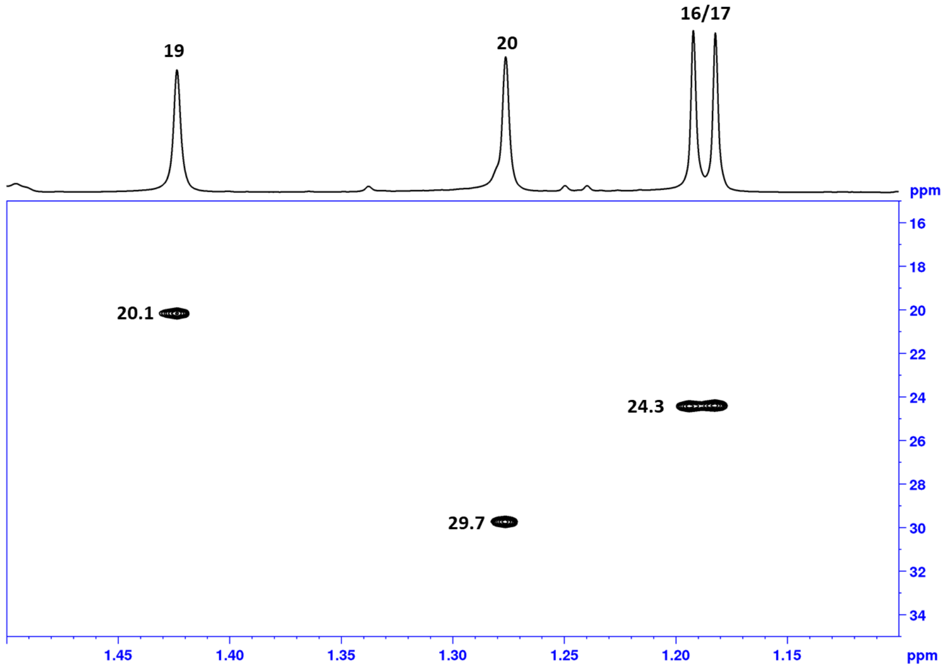

Supplemental Figure 84. Structure elucidation of **5**, ^{1}H-^{13}C HSQC spectrum, detail methyl range

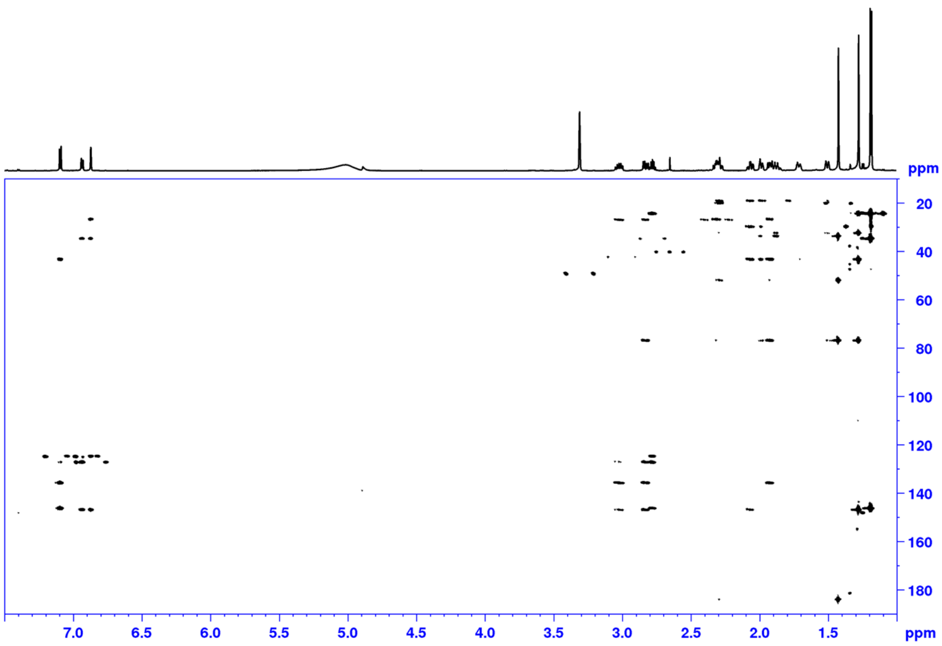

Supplemental Figure 85. Structure elucidation of **5**, ^{1}H-^{13}C HMBC spectrum, full range

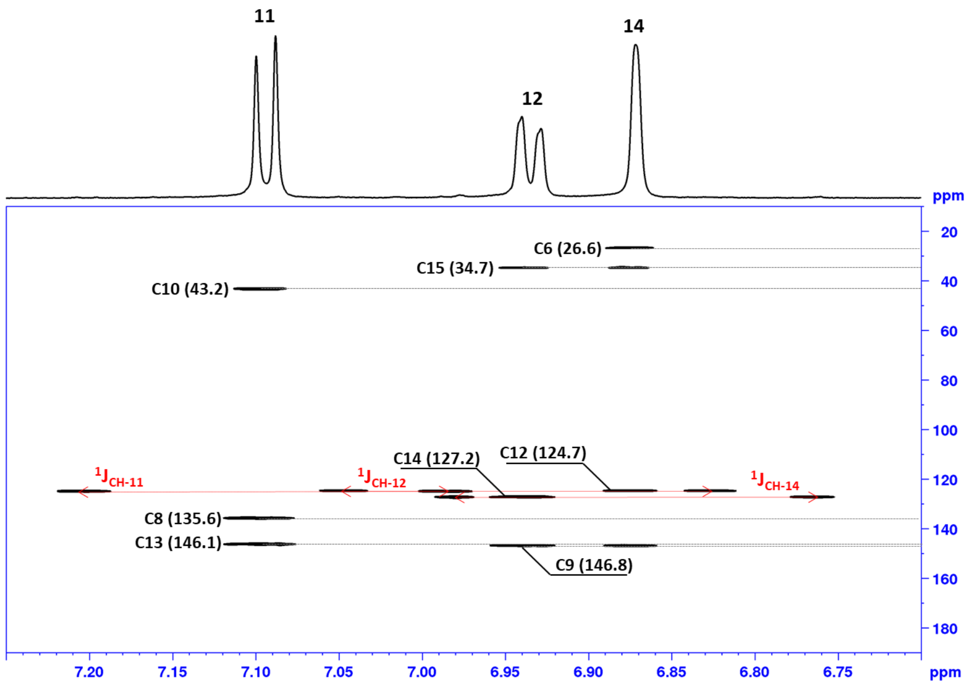

Supplemental Figure 86. Structure elucidation of **5**, ^{1}H-^{13}C HMBC spectrum, detail aromatic range

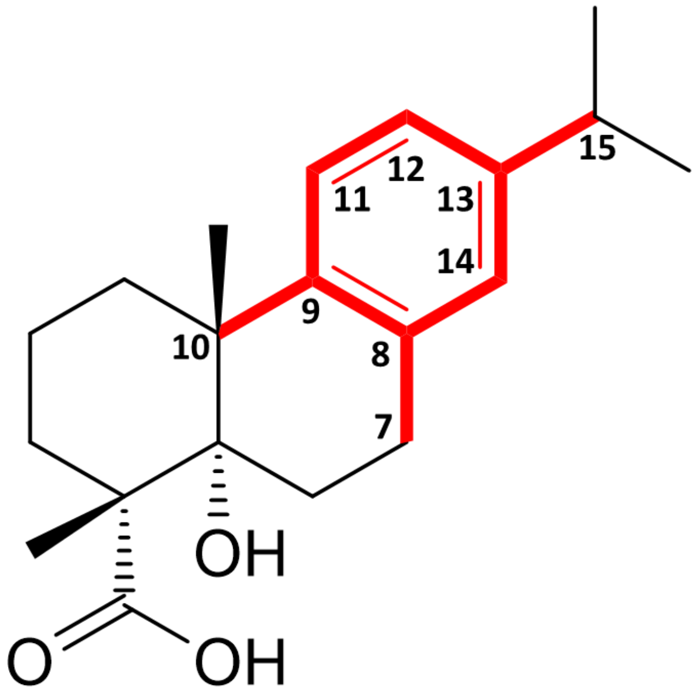

Supplemental Figure 87. Structure elucidation of **5**, structure of **5** with fragments elucidated from ^{1}H-^{13}C HMBC correlations in the aromatic range

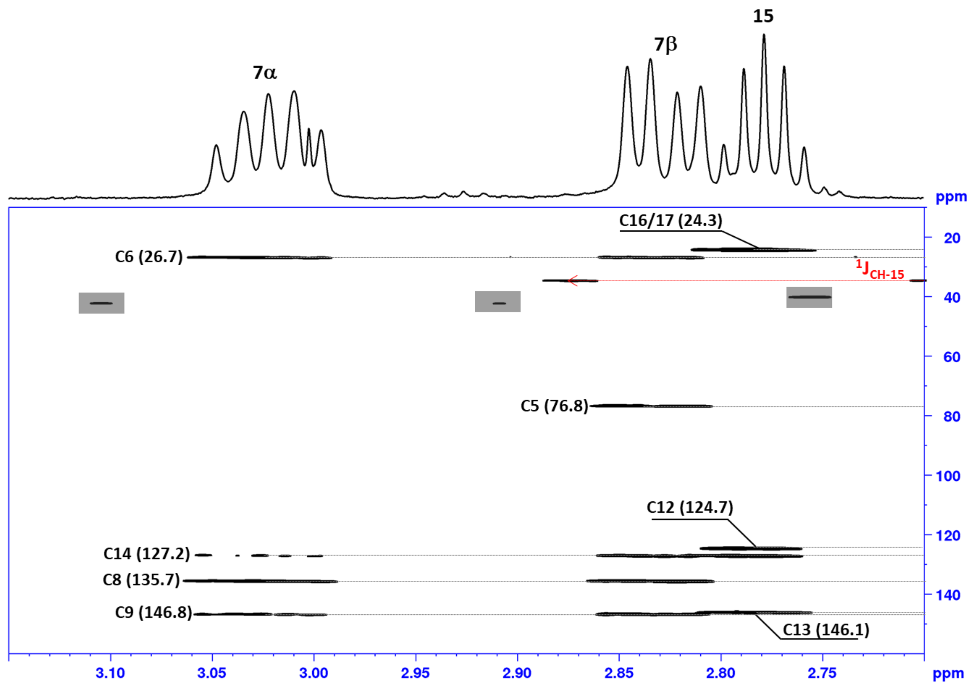

Supplemental Figure 88. Structure elucidation of **5**, ^{1}H-^{13}C HMBC spectrum, detail low field aliphatic range

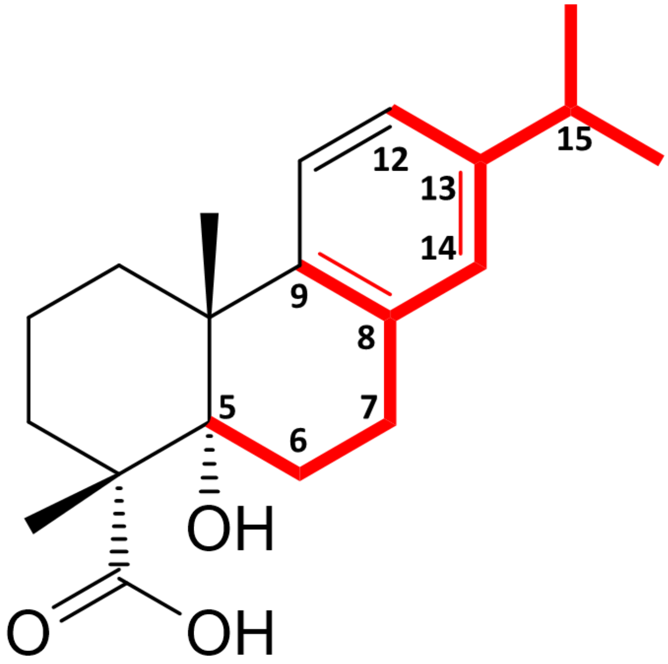

Supplemental Figure 89. Structure elucidation of **5**, structure of **5** with fragments elucidated from ^{1}H-^{13}C HMBC correlations in the low field aliphatic range

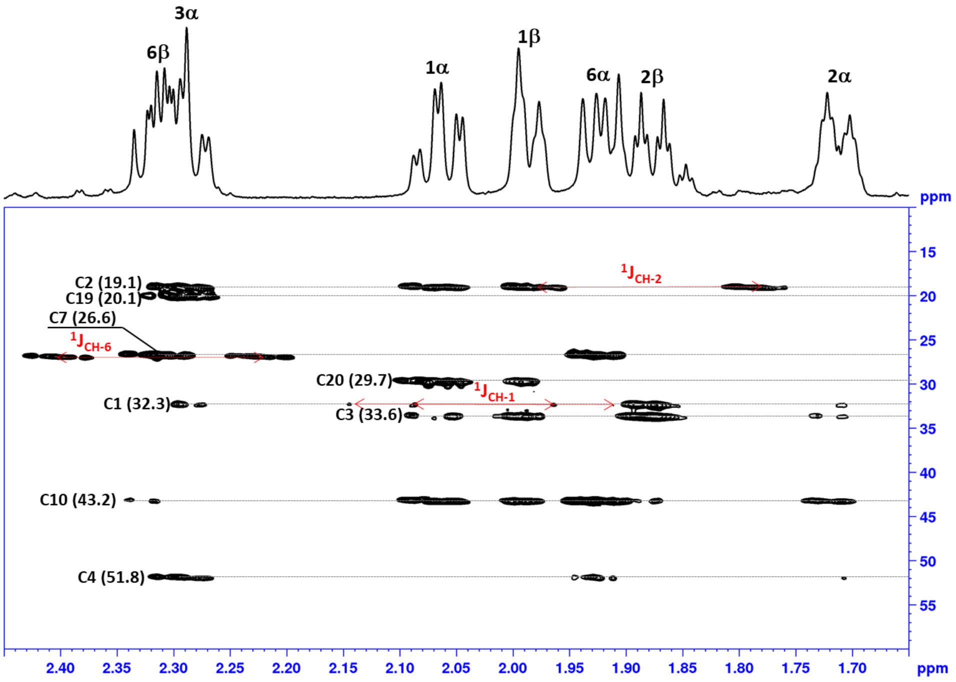

Supplemental Figure 90. Structure elucidation of **5**, ^{1}H-^{13}C HMBC spectrum, detail resonances ring A, high field ^{13}C range

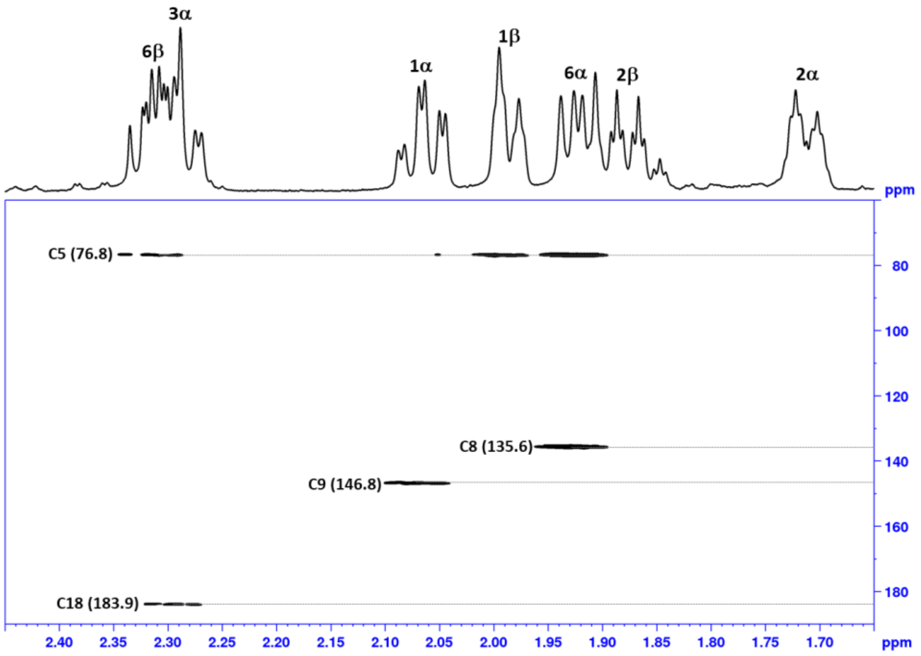

Supplemental Figure 91. Structure elucidation of **5**, ^{1}H-^{13}C HMBC spectrum, detail resonances ring A, low field ^{13}C range

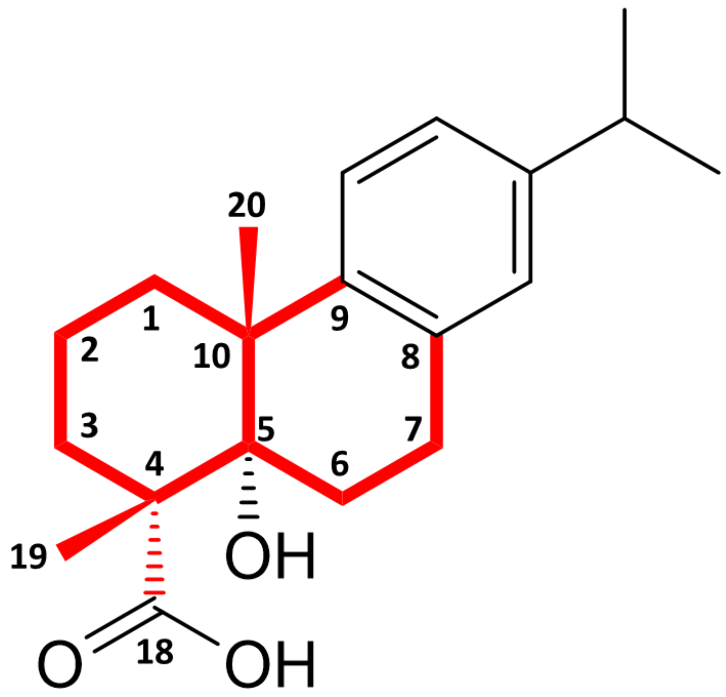

Supplemental Figure 92. Structure elucidation of **5**, structure of **5** with fragments elucidated from HMBC correlations of resonances in ring A

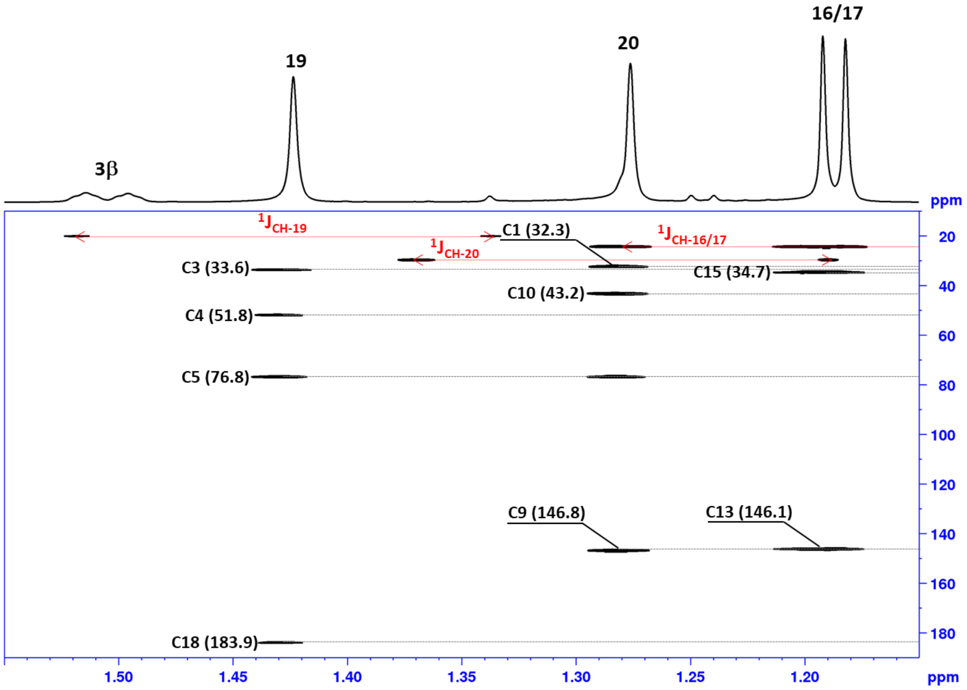

Supplemental Figure 93. Structure elucidation of **5**, ^{1}H-^{13}C HMBC spectrum, detail methyl range

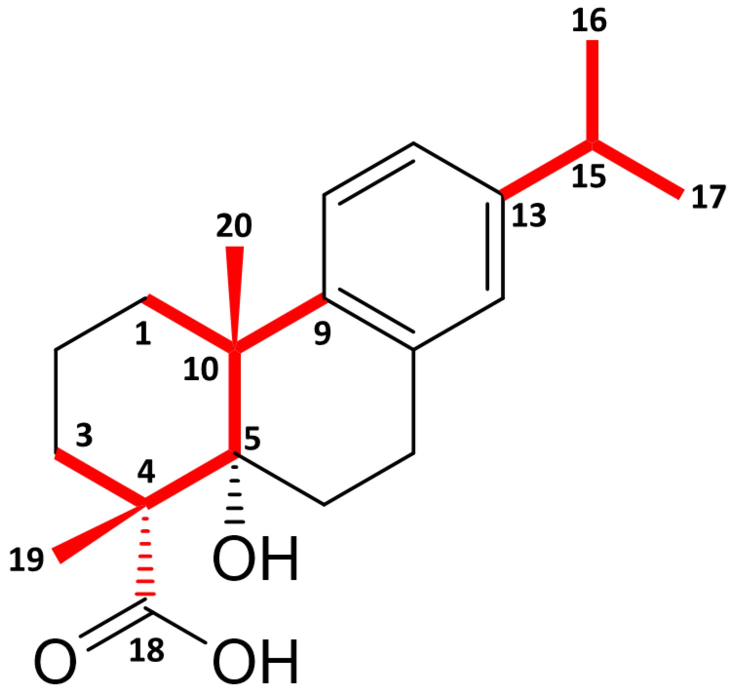

Supplemental Figure 94. Structure elucidation of **5**, structure of **5** with fragments elucidated from ^{1}H-^{13}C HMBC correlations in the methyl range

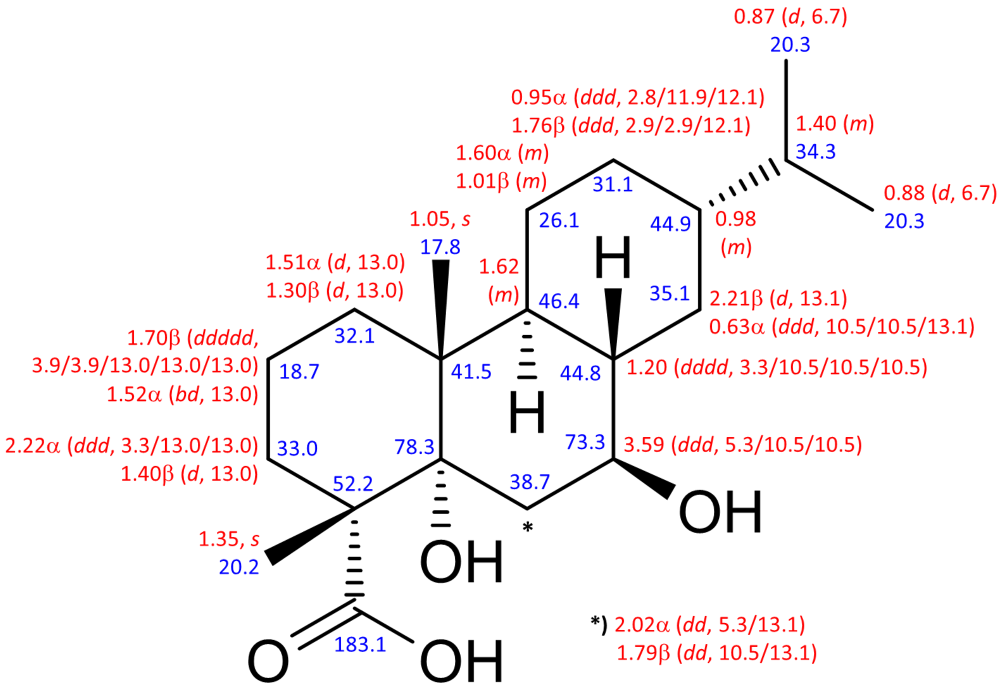

Supplemental Figure 95. Structure elucidation of **6**, overview

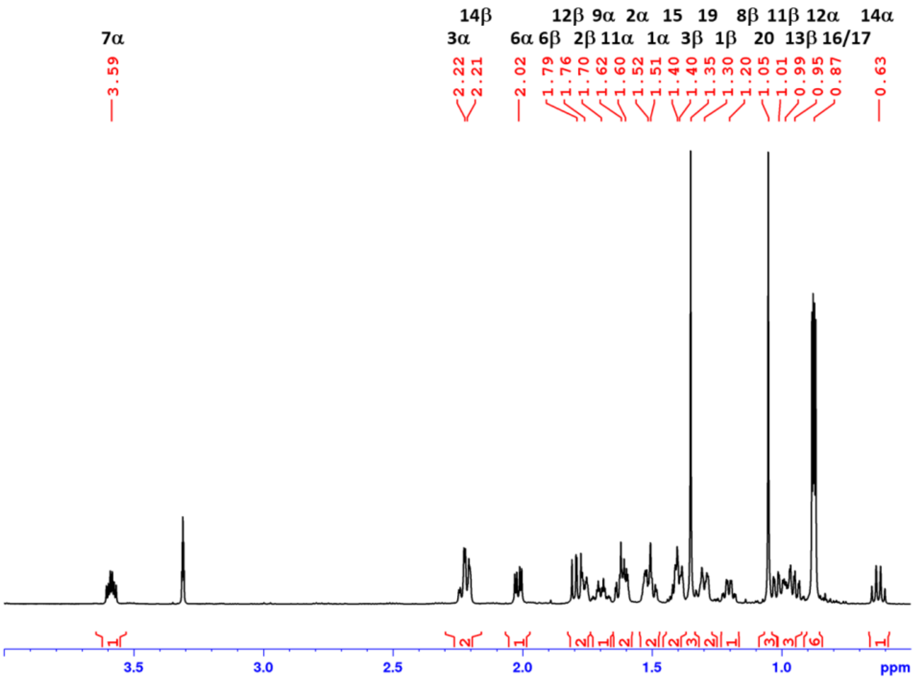

Supplemental Figure 96. Structure elucidation of **6**, ^{1}H-NMR spectrum, full range

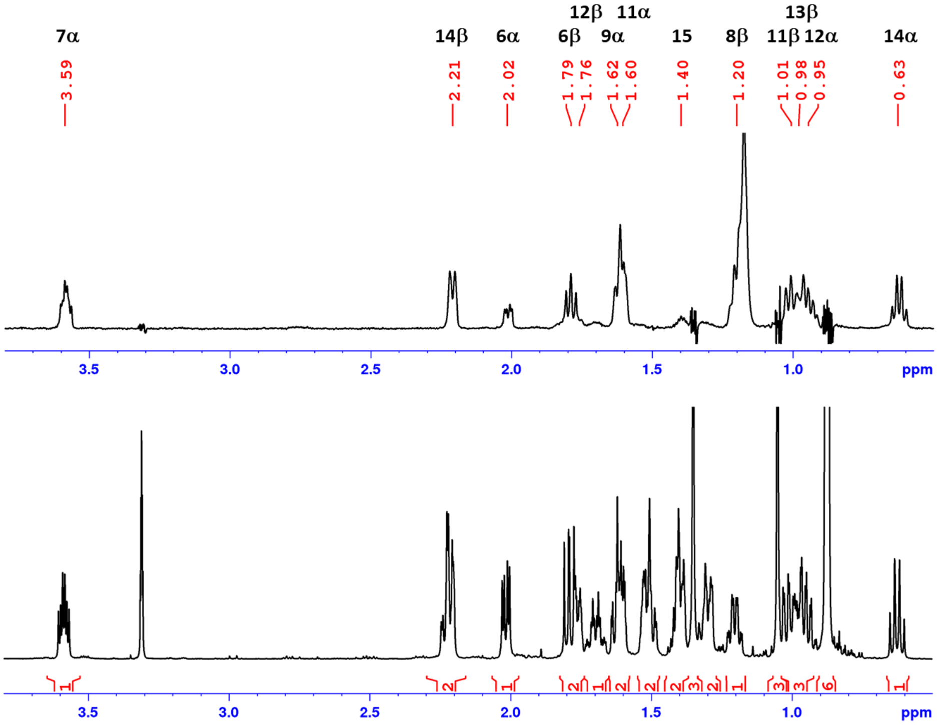

Supplemental Figure 97. Structure elucidation of **6**, ^{1}H-NMR spectrum, detail resonances of rings B and C. On top the SELTOCSY spectrum with transmitter frequency of H-7α

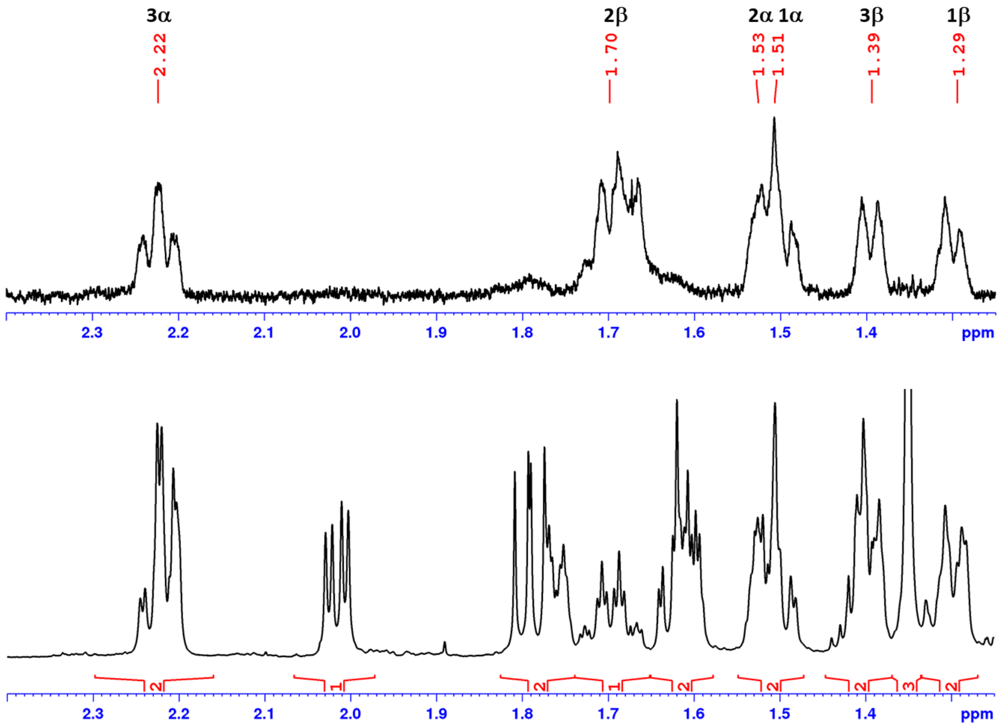

Supplemental Figure 98. Structure elucidation of **6**, ^{1}H-NMR spectrum, detail ring A. On top the SELTOCSY spectrum with transmitter frequency set on H-2β

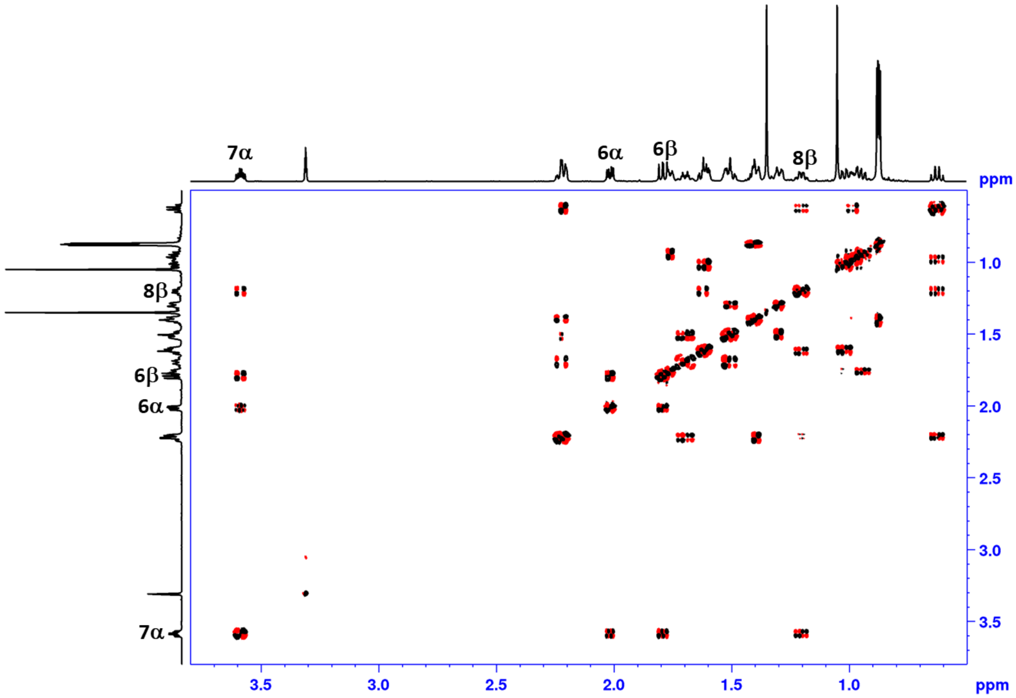

Supplemental Figure 99. Structure elucidation of **6**, ^{1}H-^{1}H COSY spectrum, full range

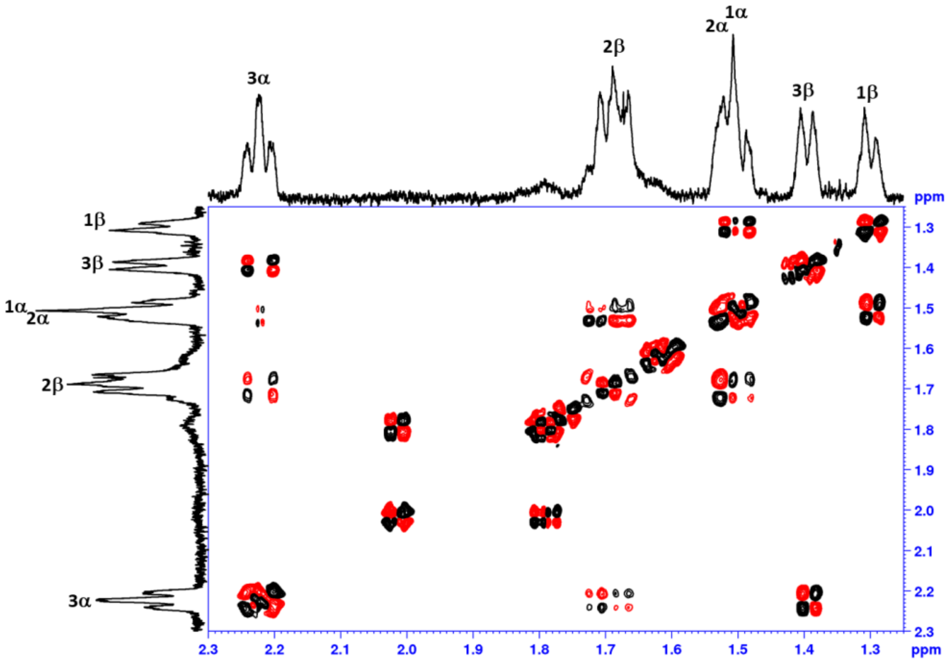

Supplemental Figure 100. Structure elucidation of **6**, ^{1}H-^{1}H COSY spectrum, detail positions 1 to 3. A SELTOCSY with the transmitter set on H-2β is used for projection in F1 and F2

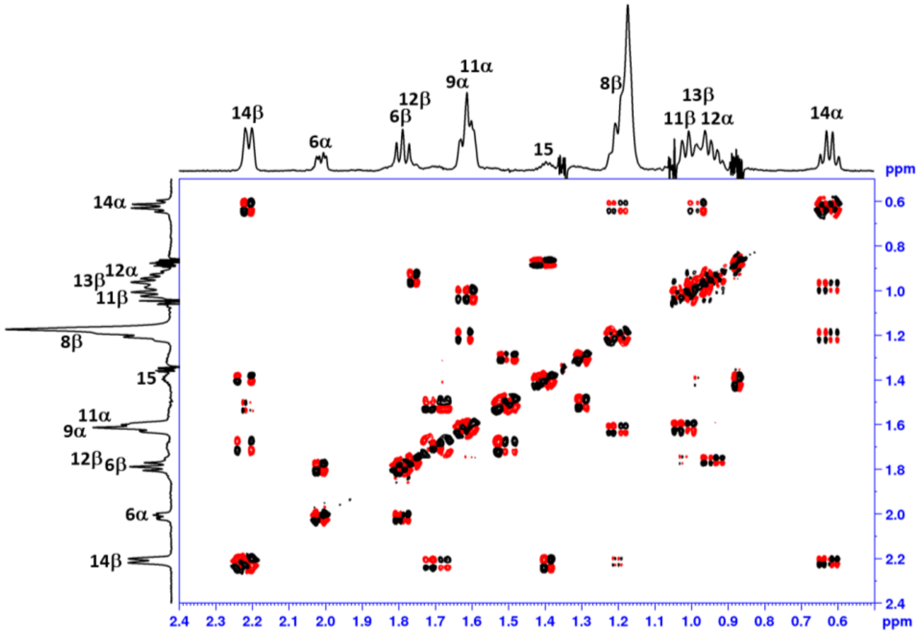

Supplemental Figure 101. Structure elucidation of **6**, ^{1}H-^{1}H COSY spectrum, detail resonances of rings B and C. A SELTOCSY with the transmitter set on H-7α is used for projection in F1 and F2

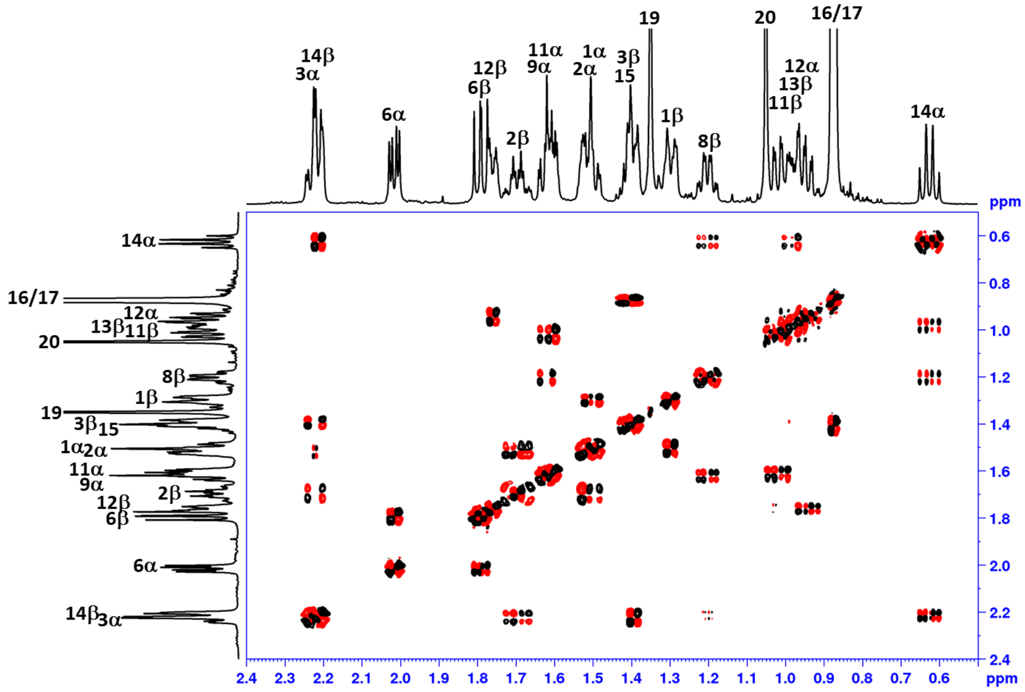

Supplemental Figure 102. Structure elucidation of **6**, ^{1}H-^{1}H COSY spectrum, detail aliphatic range

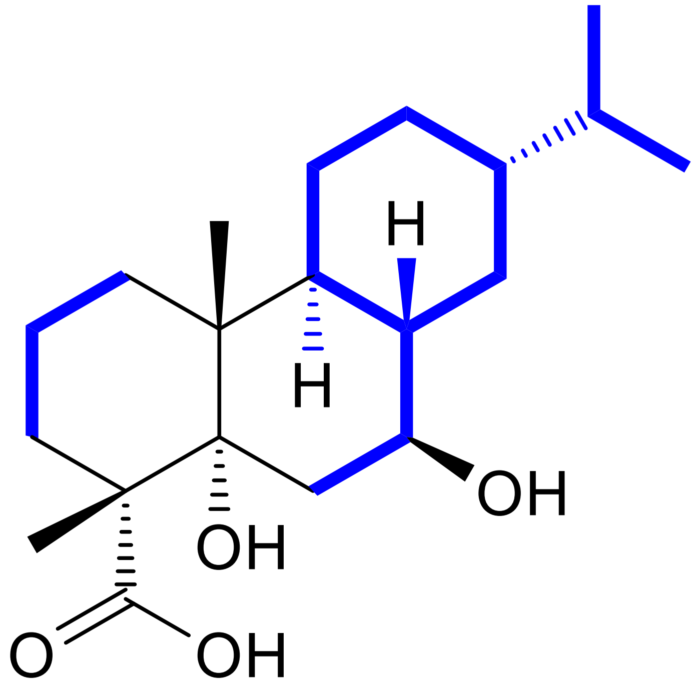

Supplemental Figure 103. Structure elucidation of **6**, structure of **6** with fragments elucidated from ^{1}H-^{1}H COSY correlations

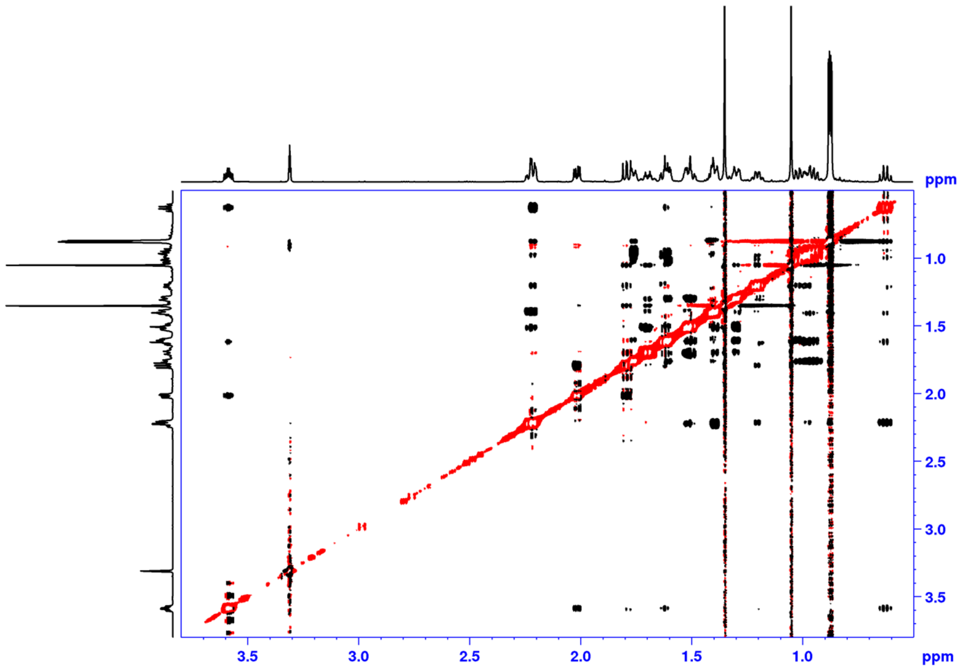

Supplemental Figure 104. Structure elucidation of **6**, ^{1}H-^{1}H ROESY spectrum, full range

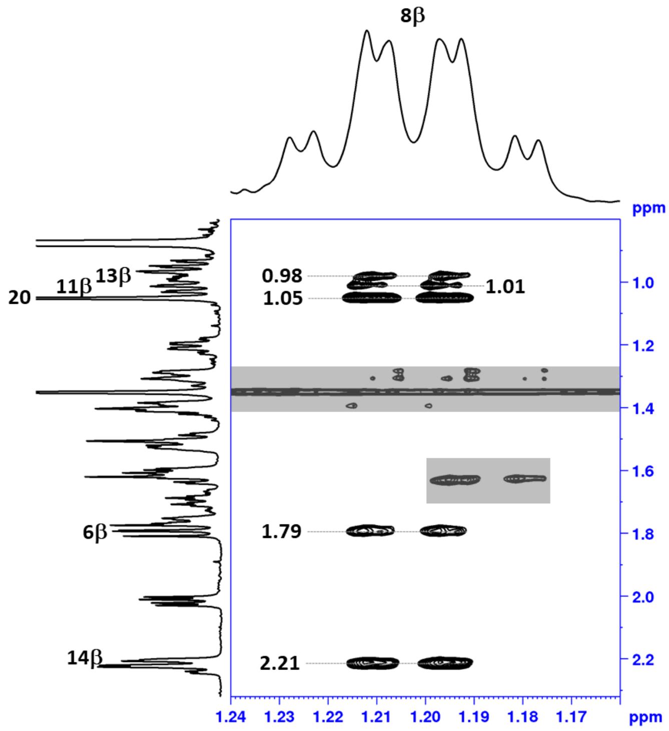

Supplemental Figure 105. Structure elucidation of **6**, ^{1}H-^{1}H ROESY spectrum, detail correlations for H-8β. Signals from impurities are covered (grey)

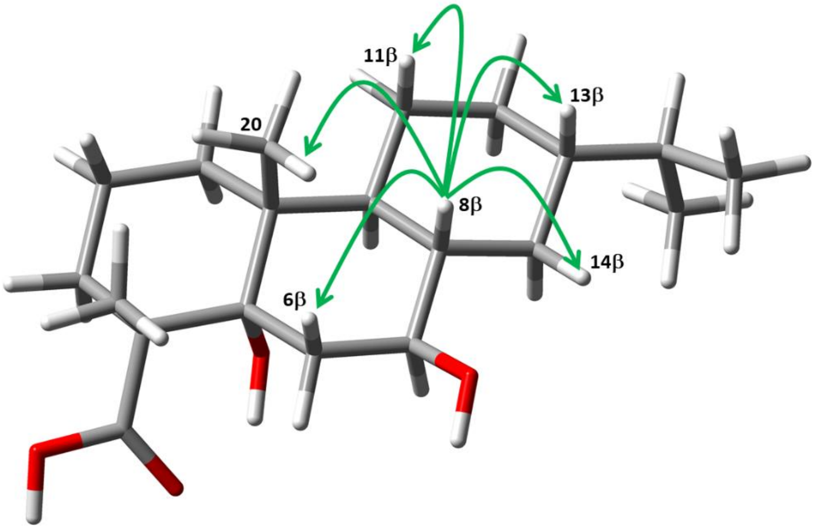

Supplemental Figure 106. Structure elucidation of **6**, structure of **6** with important ROESY correlations of β-oriented substituents of rings B and C

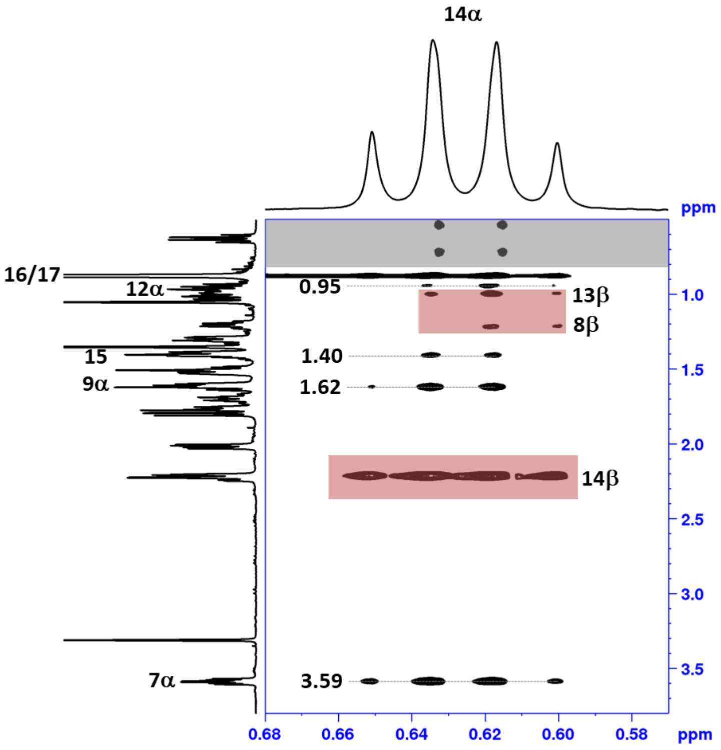

Supplemental Figure 107. Structure elucidation of **6**, ^{1}H-^{1}H ROESY spectrum, detail correlations for H-14α. The covered signals (red) represent COSY-like correlations

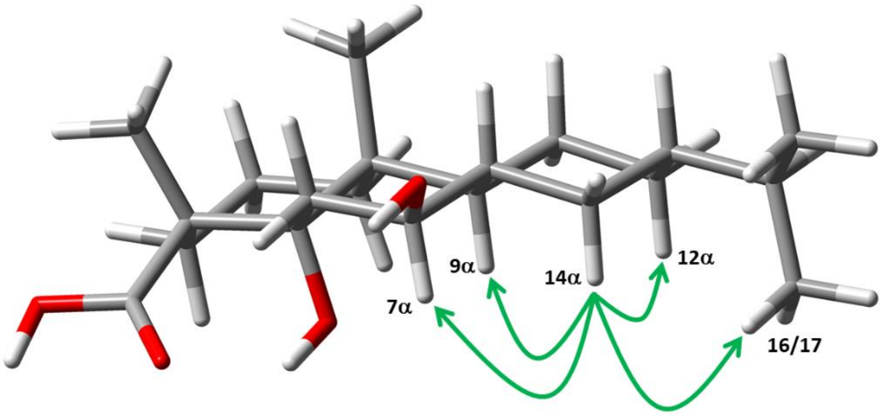

Supplemental Figure 108. Structure elucidation of **6**, structure of **6** with important ROESY correlations of α-oriented substituents of rings B and C

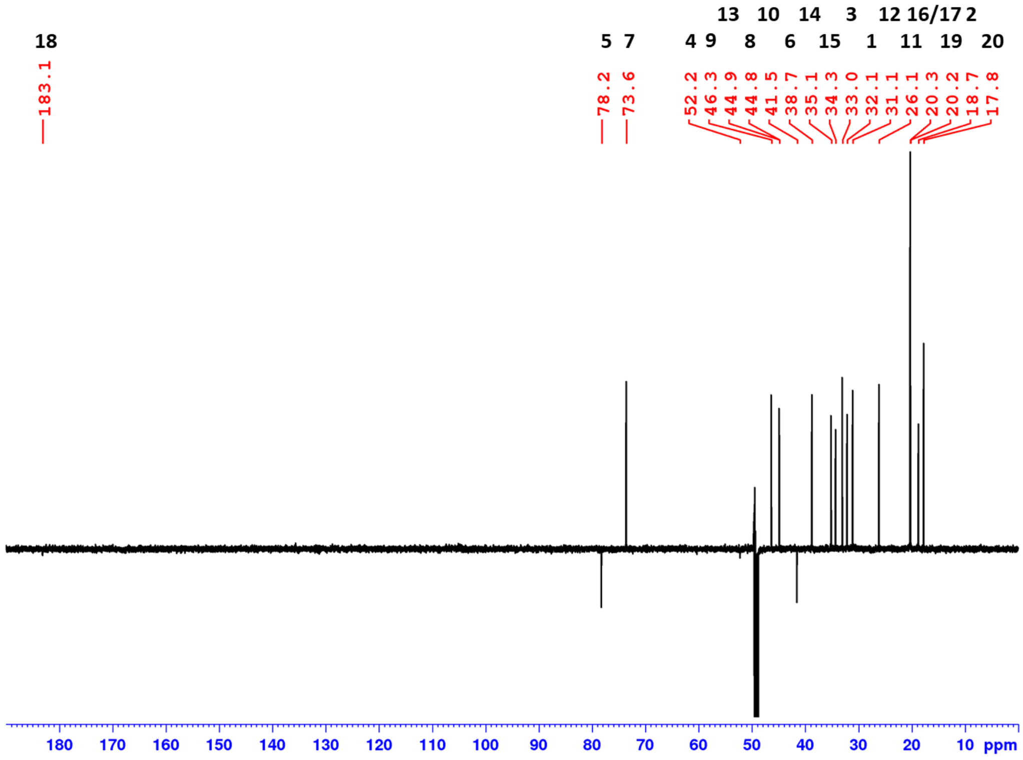

Supplemental Figure 109. Structure elucidation of **6**, ^{13}C-DEPTQ spectrum

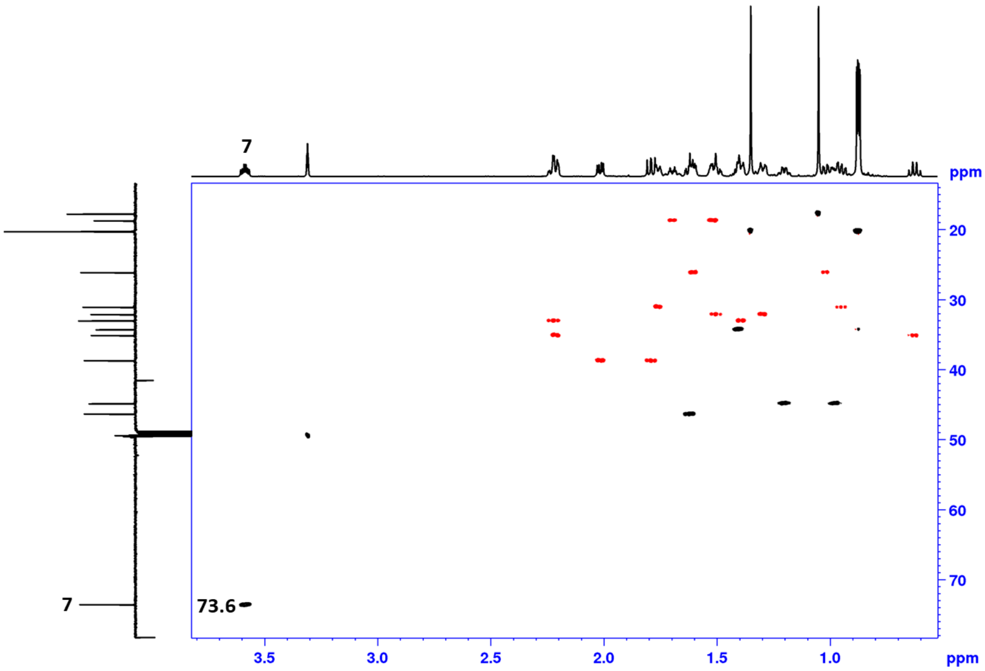

Supplemental Figure 110. Structure elucidation of **6**, ^{1}H-^{13}C HSQC spectrum, full range

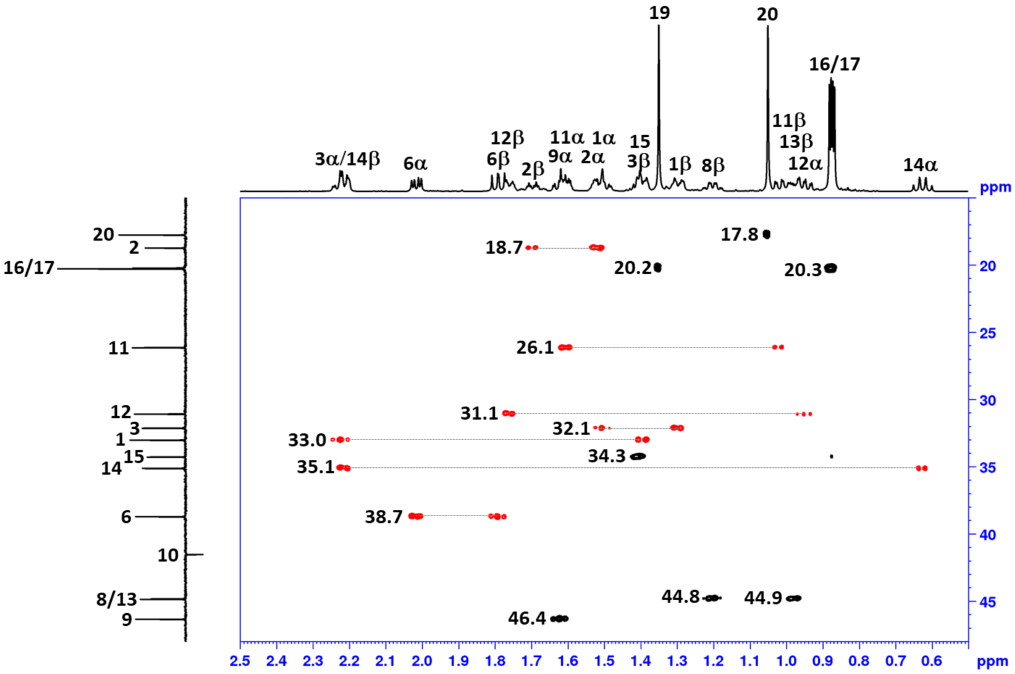

Supplemental Figure 111. Structure elucidation of **6**, ^{1}H-^{13}C HSQC spectrum, detail

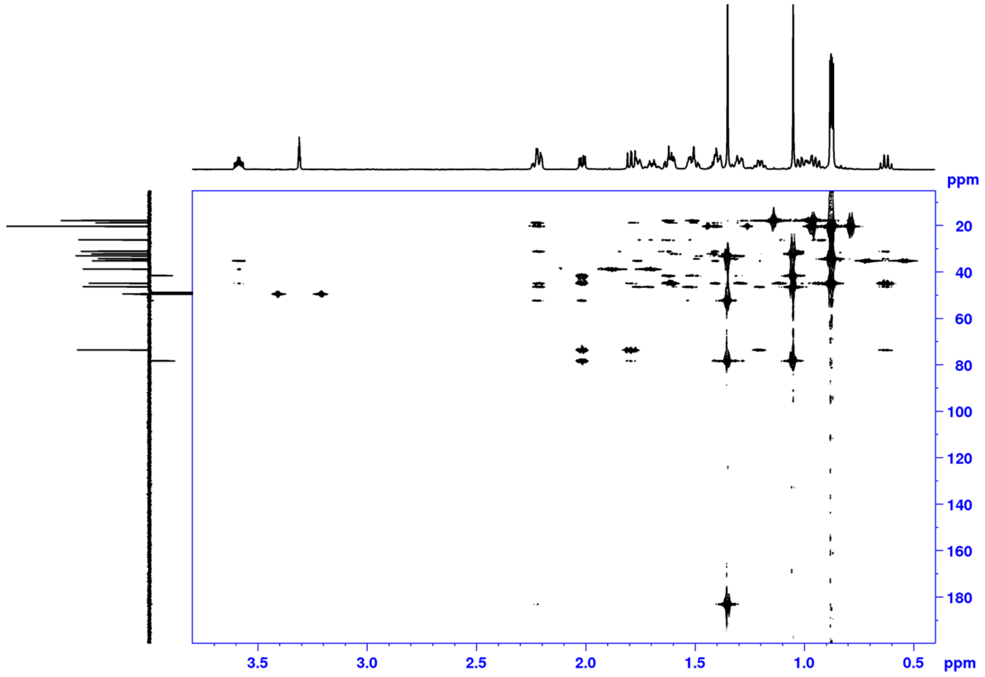

Supplemental Figure 112. Structure elucidation of **6**, ^{1}H-^{13}C HMBC spectrum, full range

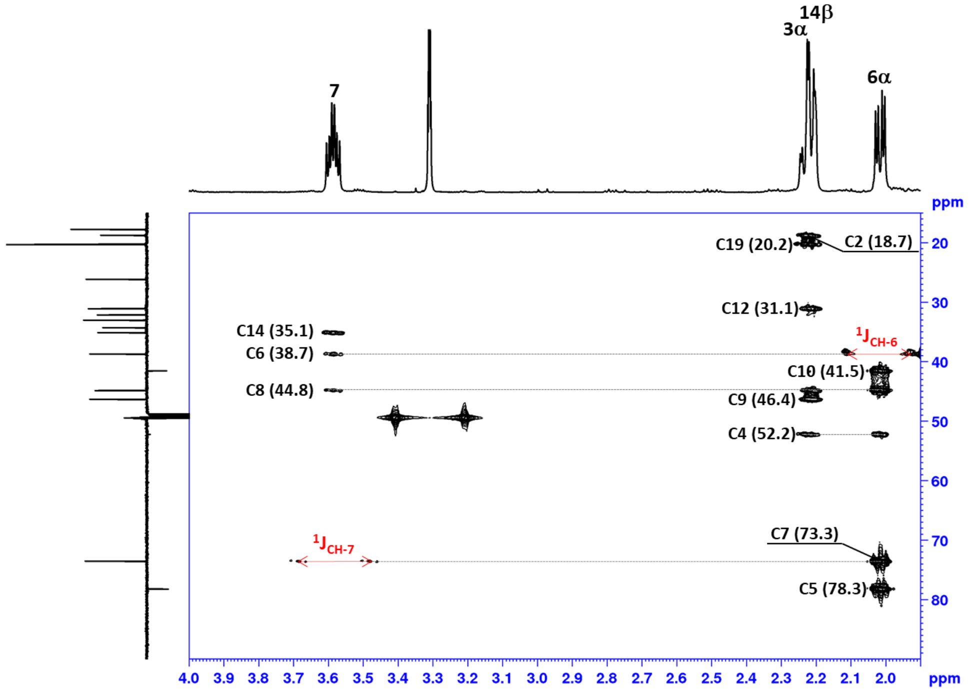

Supplemental Figure 113. Structure elucidation of **6**, ^{1}H-^{13}C HMBC spectrum, detail aliphatic range (δ_H 4—2)

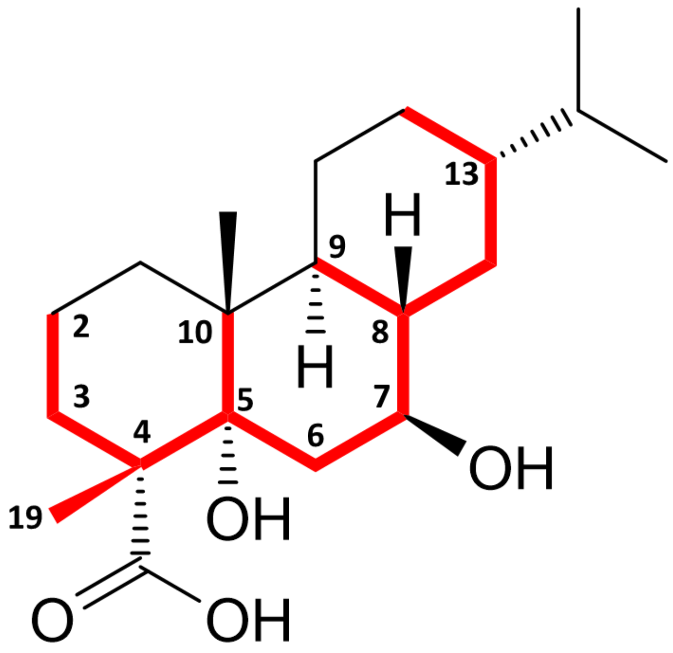

Supplemental Figure 114. Structure elucidation of **6**, structure of **6** with fragments elucidated from HMBC correlations in the aliphatic range (δ_H 4—2)

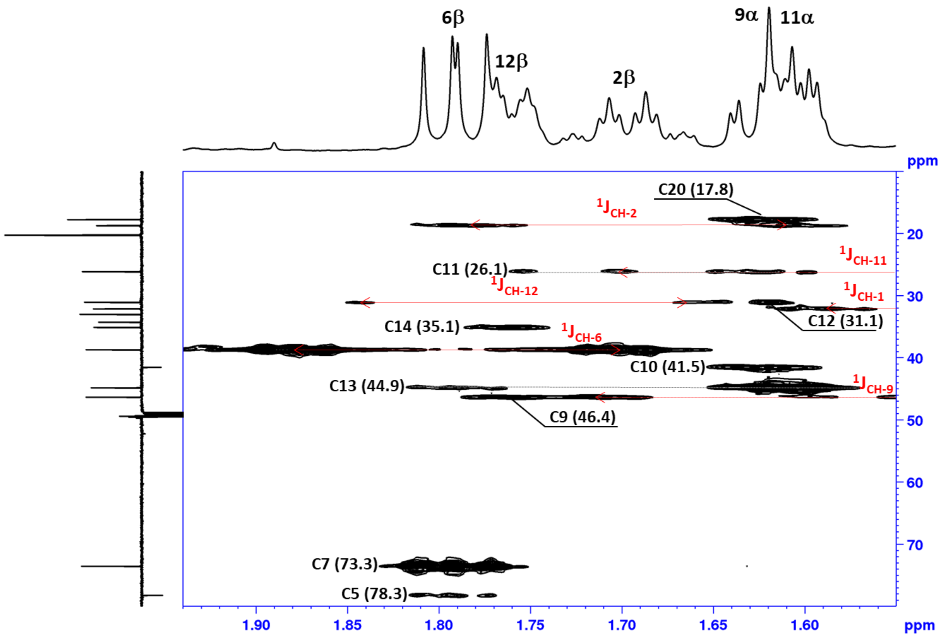

Supplemental Figure 115. Structure elucidation of **6**, ^{1}H-^{13}C HMBC spectrum, detail aliphatic range (δ_H 2—1.5)

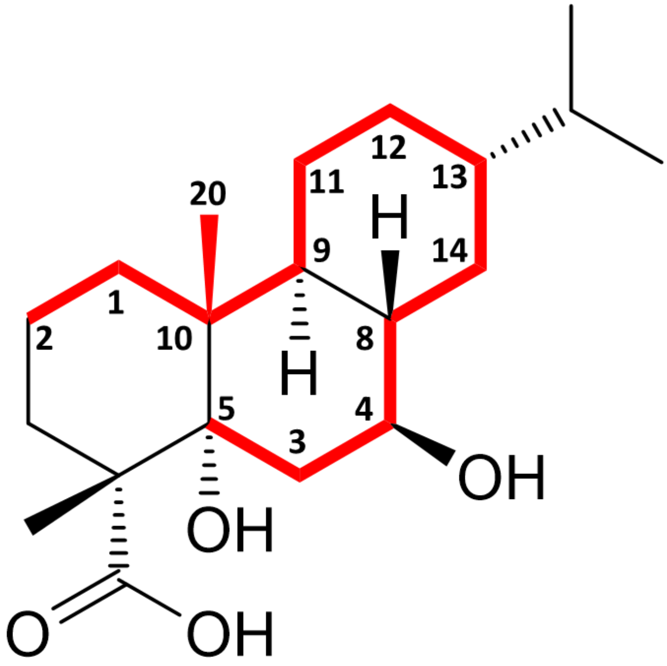

Supplemental Figure 116. Structure elucidation of **6**, structure of **6** with fragments elucidated from HMBC correlations in the aliphatic range (δ_H 2—1.5)

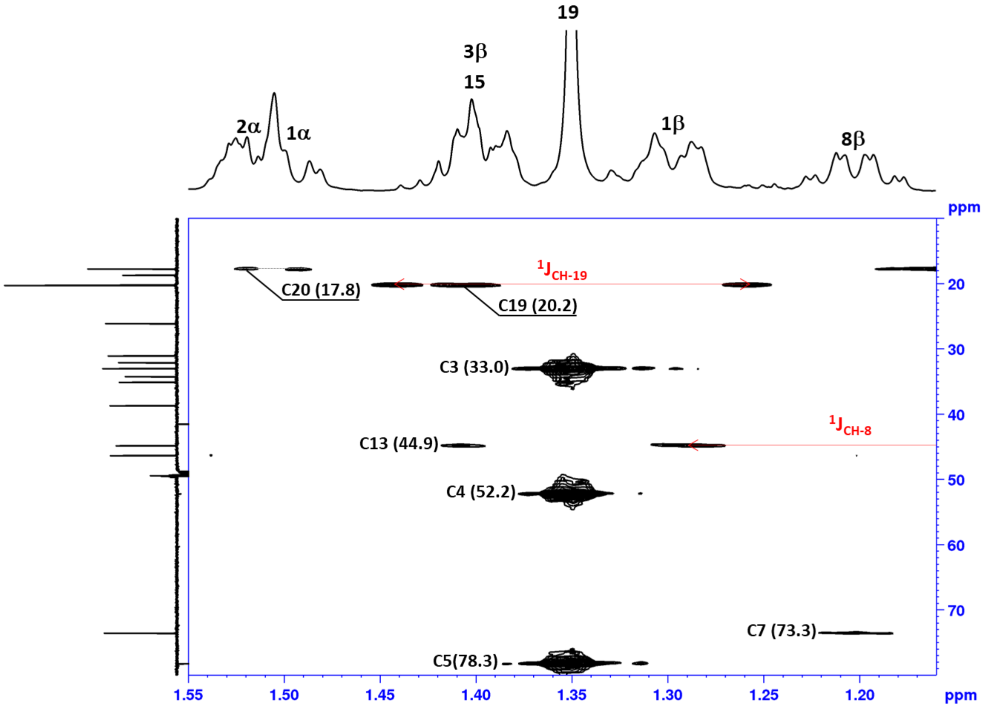

Supplemental Figure 117. Structure elucidation of **6**, ^{1}H-^{13}C HMBC spectrum, detail aliphatic range (δ_H 1.5—1.15)

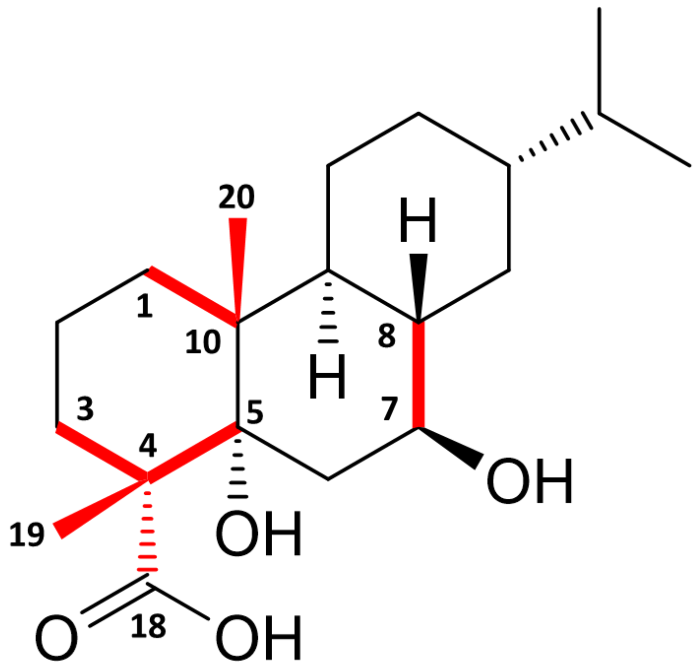

Supplemental Figure 118. Structure elucidation of **6**, structure of **6** with fragments elucidated from HMBC correlations in the aliphatic range (δ_H 1.5—1.15)

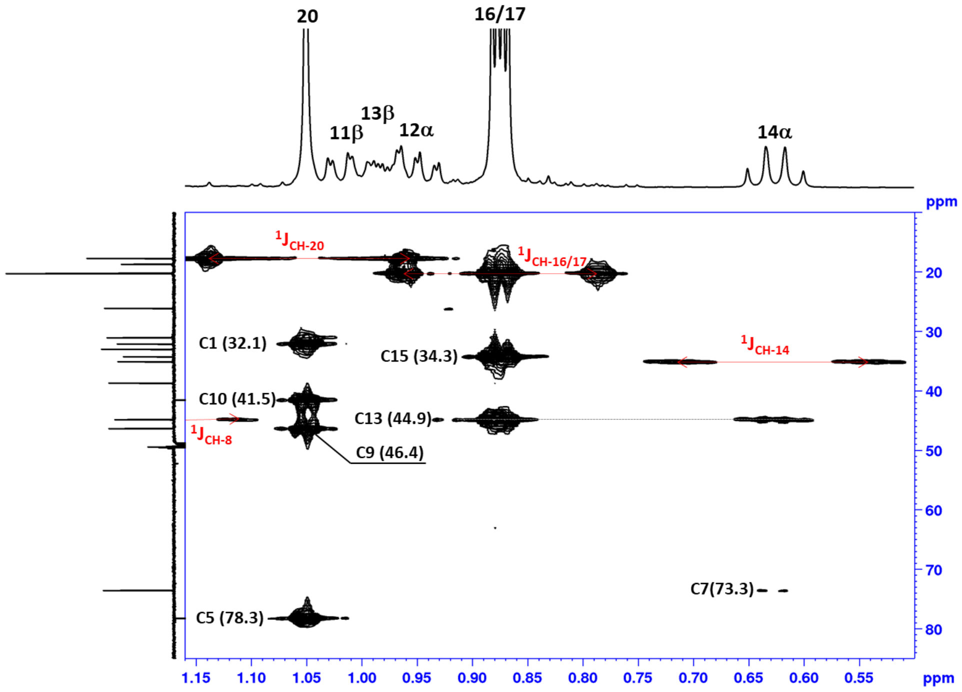

Supplemental Figure 119. Structure elucidation of **6**, ^{1}H-^{13}C HMBC spectrum, detail aliphatic range (δ_H 1.15—0.5)

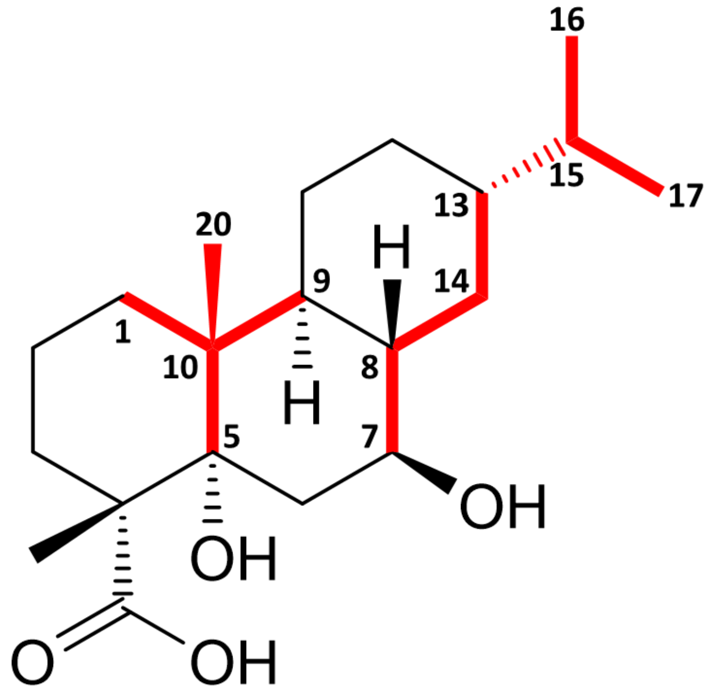

Supplemental Figure 120. Structure elucidation of **6**, structure of **6** with fragments elucidated from HMBC correlations in the aliphatic range (δ_H 1.15—0.5)

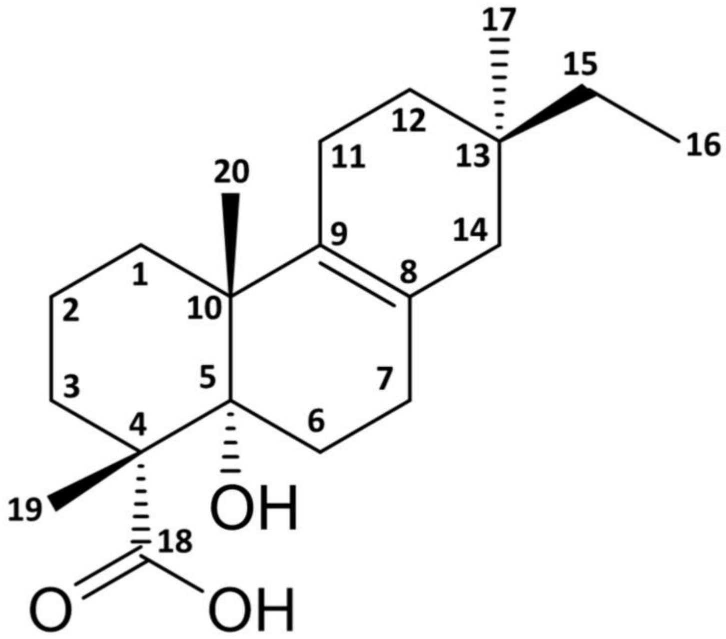

Supplemental Figure 121. Structure elucidation of **7**, numbering of **7**

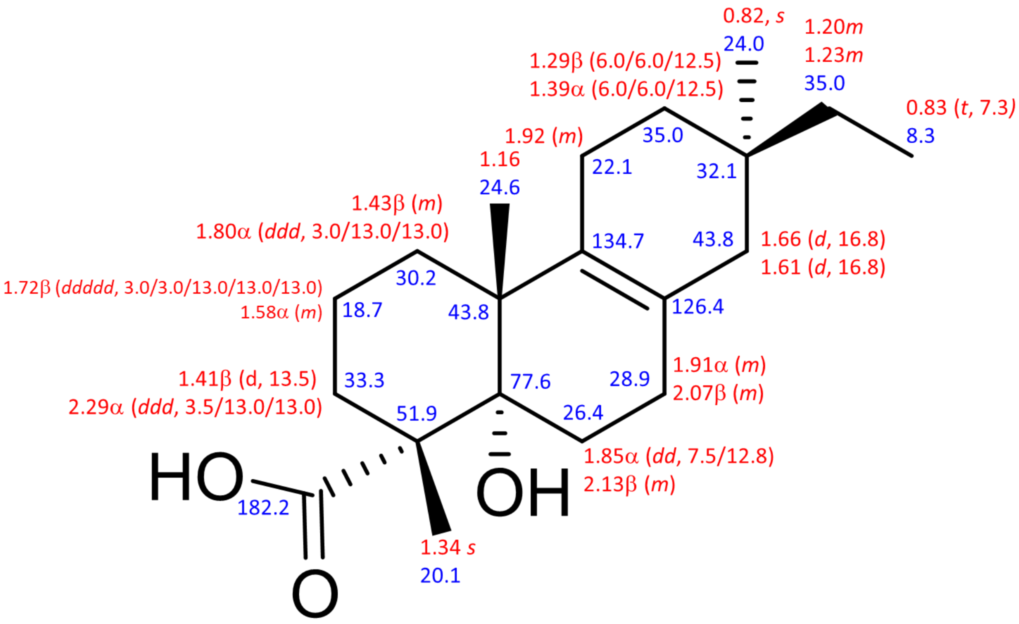

Supplemental Figure 122. Structure elucidation of **7**, overview

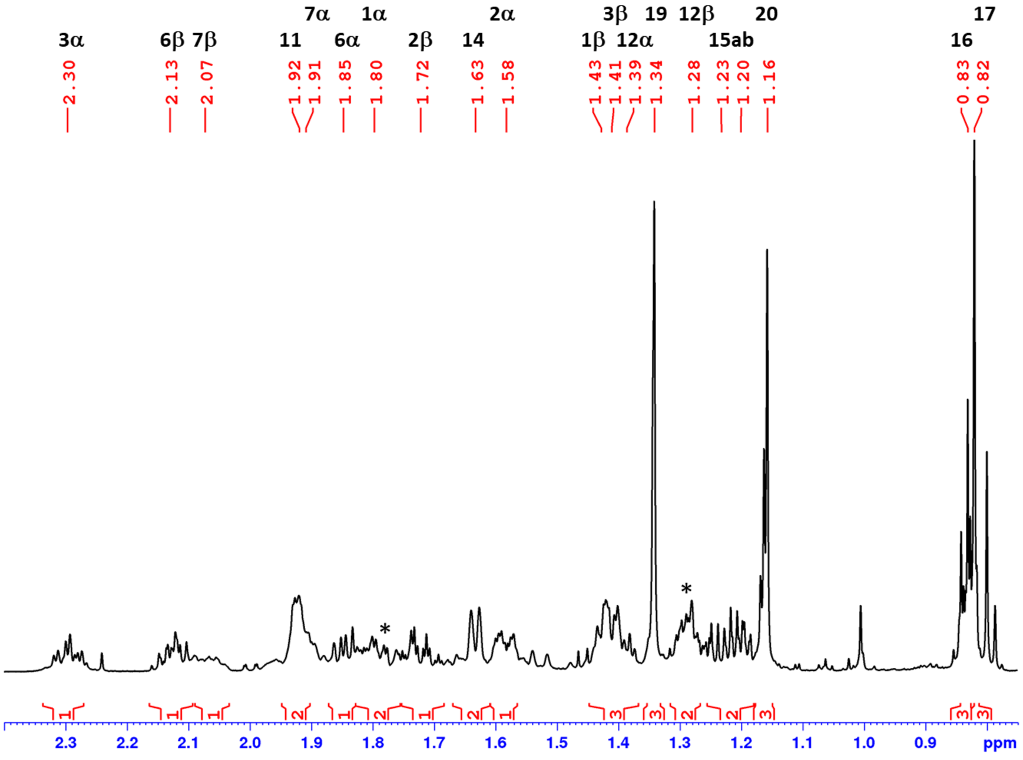

Supplemental Figure 123. Structure elucidation of **7**, ^{1}H-NMR spectrum, full range. Asterisks mark impurities that increase the integrals.

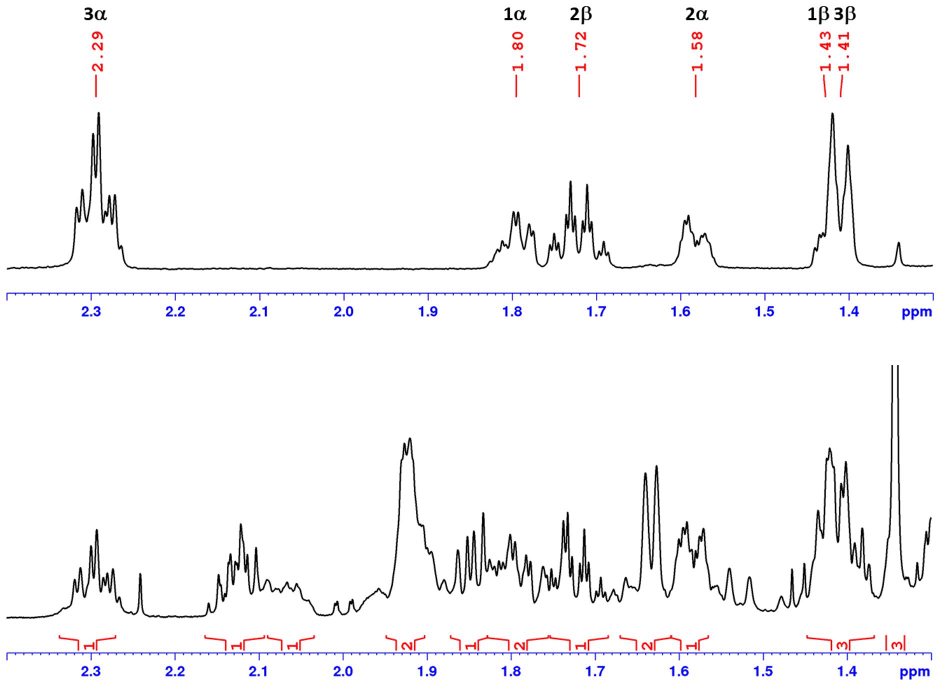

Supplemental Figure 124. Structure elucidation of **7**, comparison ^{1}H-NMR spectrum, detail positions 1 to 3. The upper spectrum is the SELTOCSY with the transmitter set on resonance with H-3α

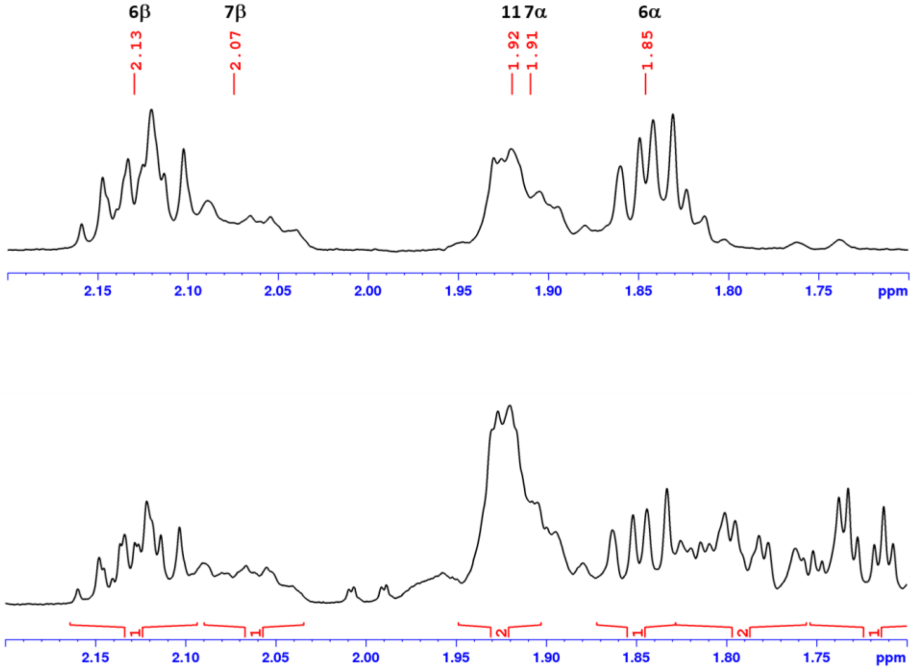

Supplemental Figure 125. Structure elucidation of **7**, comparison ^{1}H-NMR spectrum, detail positions 6 and 7. The upper spectrum is the SELTOCSY with the transmitter set on resonance with H-6α

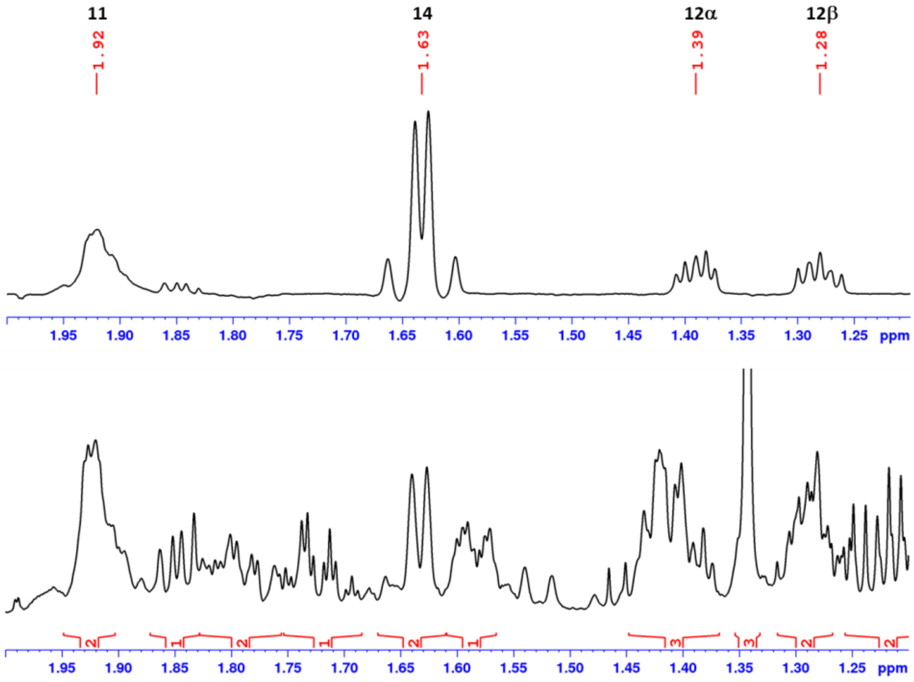

Supplemental Figure 126. Structure elucidation of **7**, comparison ^{1}H-NMR spectrum, detail resonances ring C. The upper spectrum is the SELTOCSY with the transmitter set on resonance with H-14αβ.

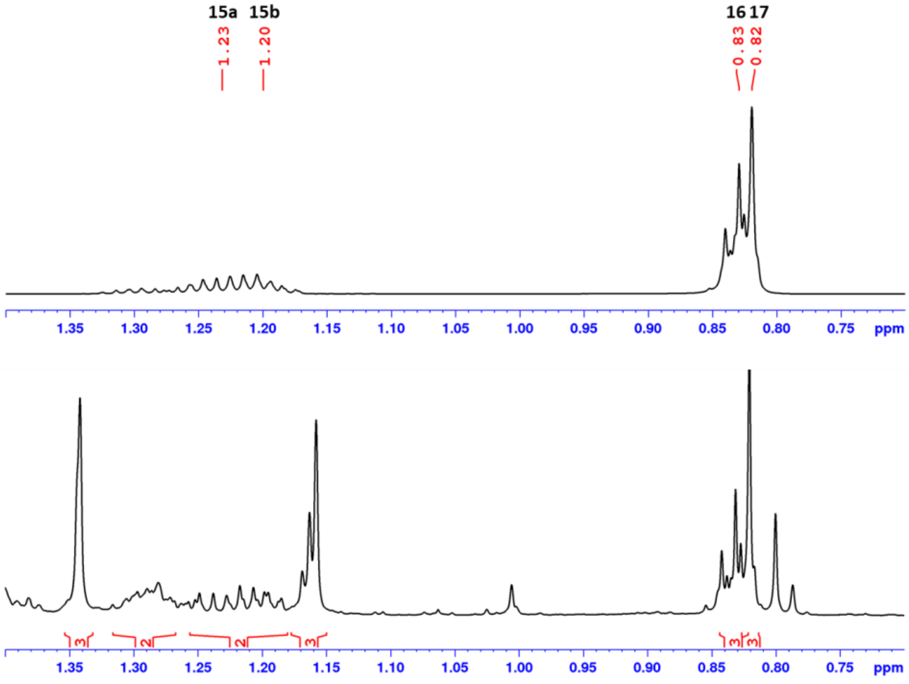

Supplemental Figure 127. Structure elucidation of **7**, comparison ^{1}H-NMR spectrum, detail side chain. The upper spectrum is the SELTOCSY with the transmitter set on resonance with H-15ab.

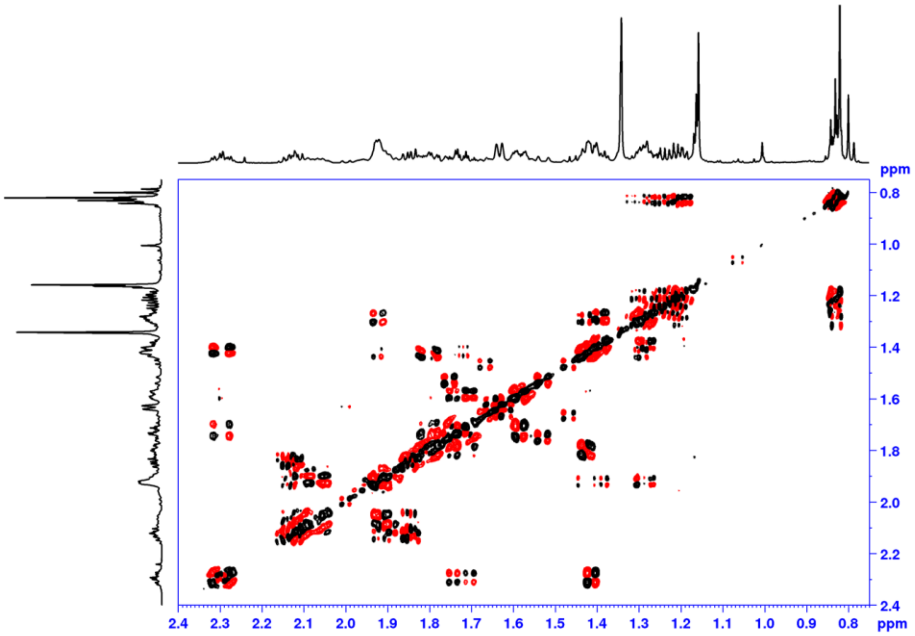

Supplemental Figure 128. Structure elucidation of **7**, ^{1}H-^{1}H COSY spectrum, full range

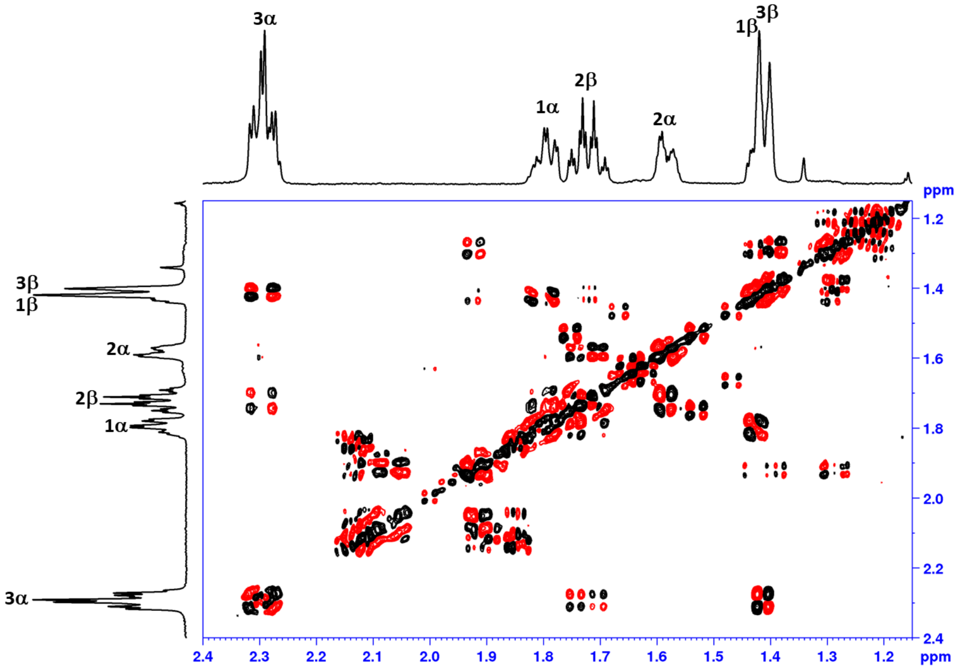

Supplemental Figure 129. Structure elucidation of **7**, ^{1}H-^{1}H COSY spectrum, detail aliphatic range, ring A. The SELTOCSY with the transmitter set on resonance with H-3α is used for the F1 and F2 projections.

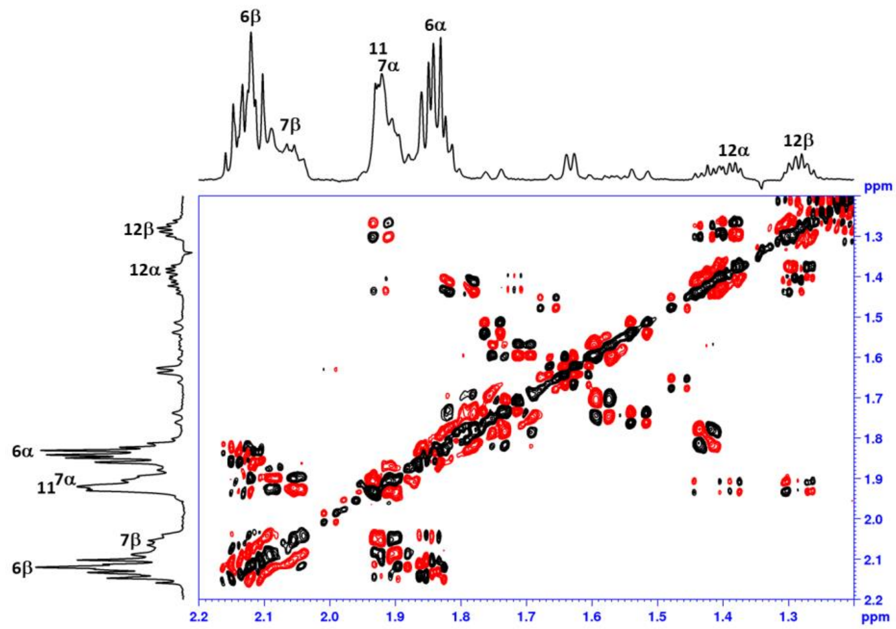

Supplemental Figure 130. Structure elucidation of **7**, ^{1}H-^{1}H COSY spectrum, detail aliphatic range, ring B. The SELTOCSY with the transmitter set on resonance with H-6α is used for the F1 and F2 projections.

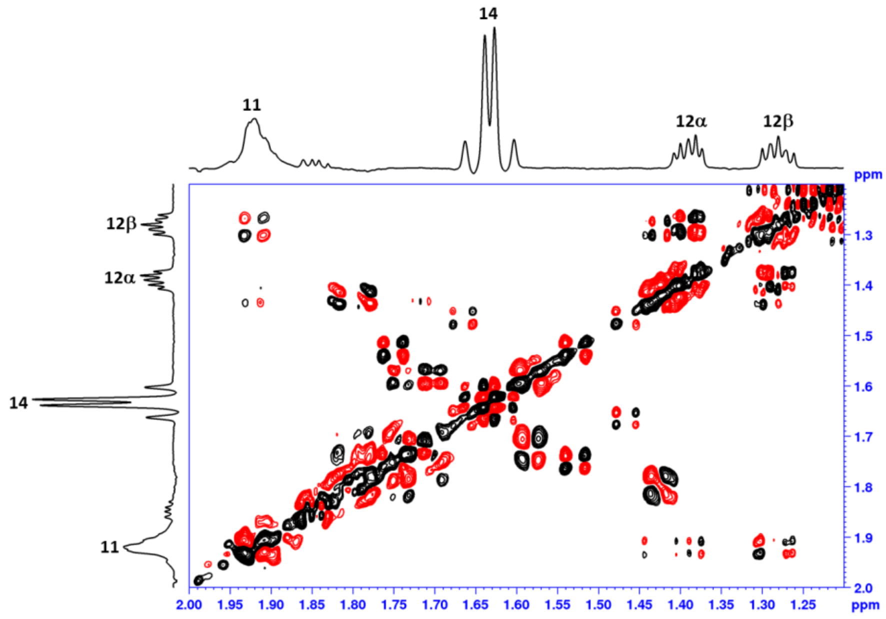

Supplemental Figure 131. Structure elucidation of **7**, ^{1}H-^{1}H COSY spectrum, detail aliphatic range, ring C. The SELTOCSY with the transmitter set on resonance with H-14αβ is used for the F1 and F2 projections.

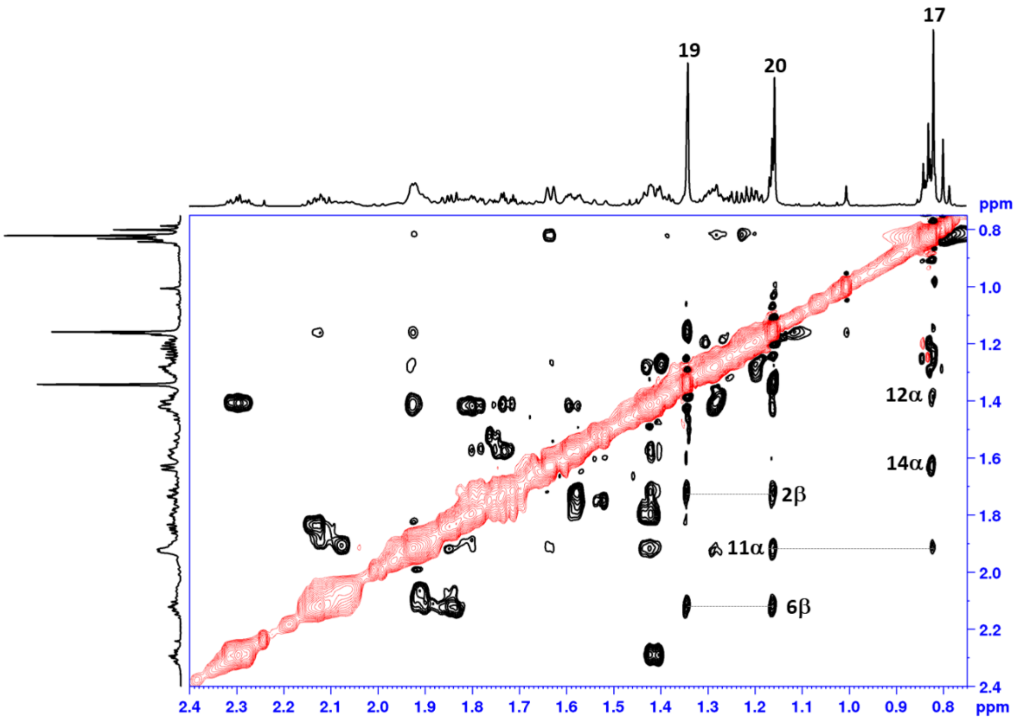

Supplemental Figure 132. Structure elucidation of **7**, ^{1}H-^{1}H ROESY spectrum, full range

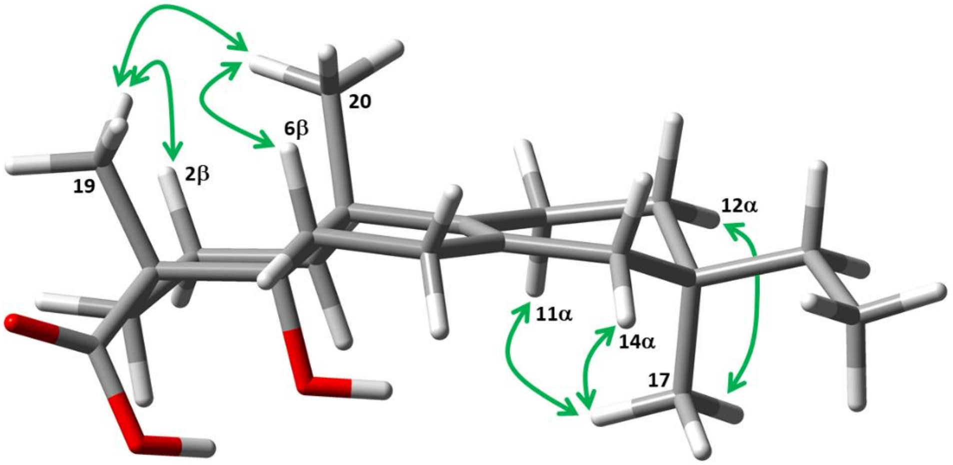

Supplemental Figure 133. Structure elucidation of **7**, structure of **7** with important ROESY correlations

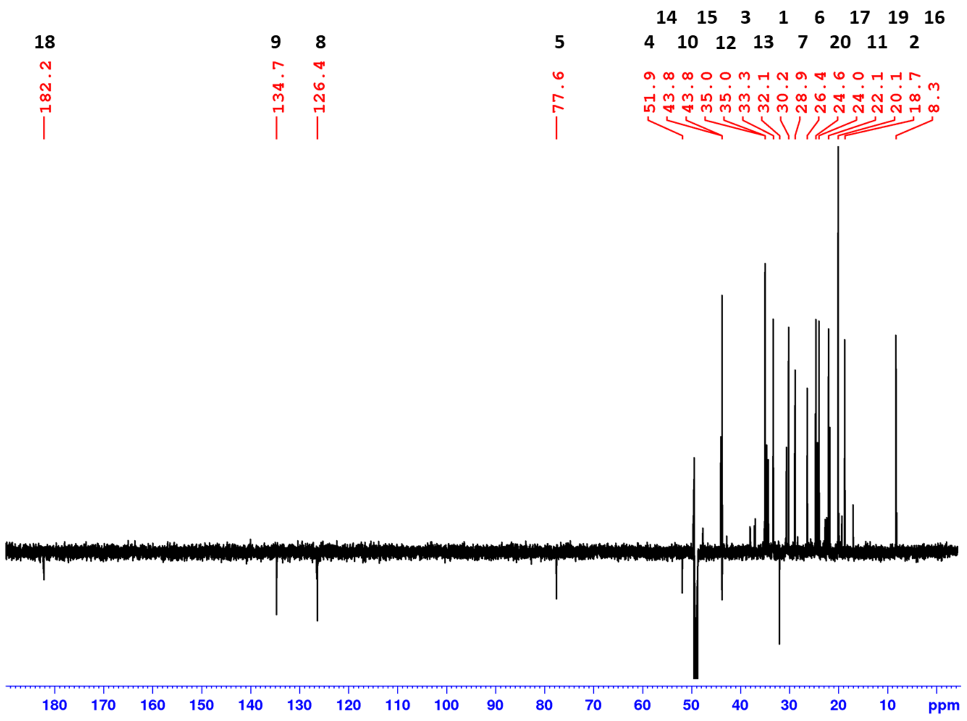

Supplemental Figure 134. Structure elucidation of **7**, ^{13}C-DEPTQ spectrum

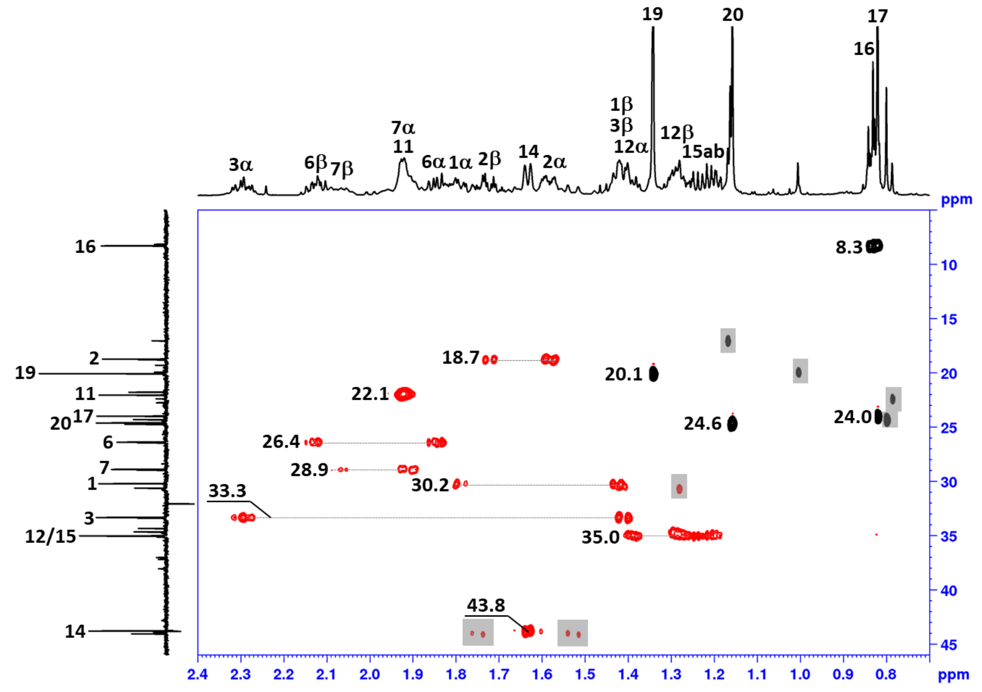

Supplemental Figure 135. Structure elucidation of **7**, ^{1}H-^{13}C HSQC spectrum, full range

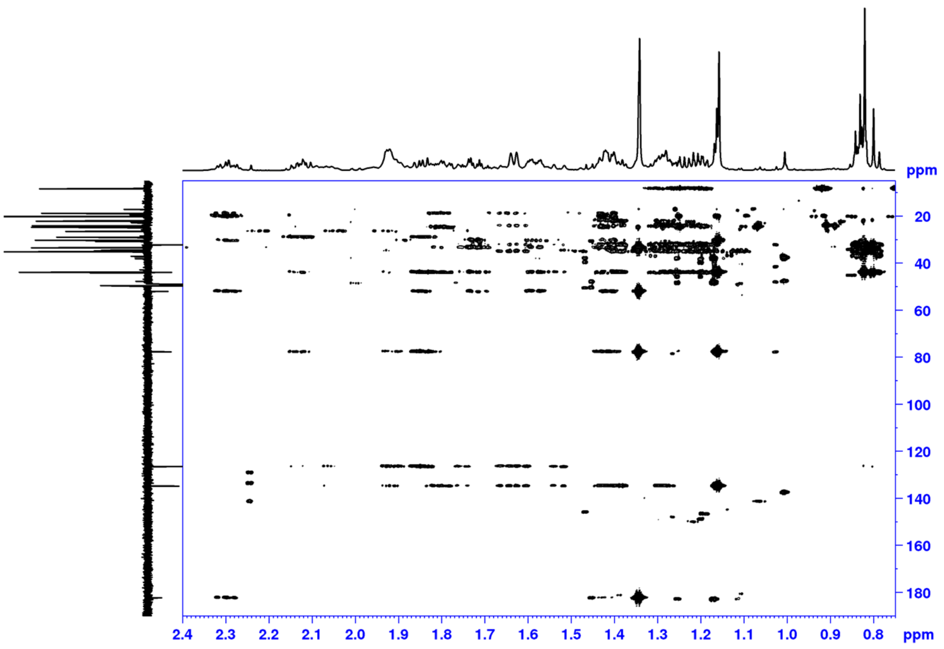

Supplemental Figure 136. Structure elucidation of **7**, ^{1}H-^{13}C HMBC spectrum, full range

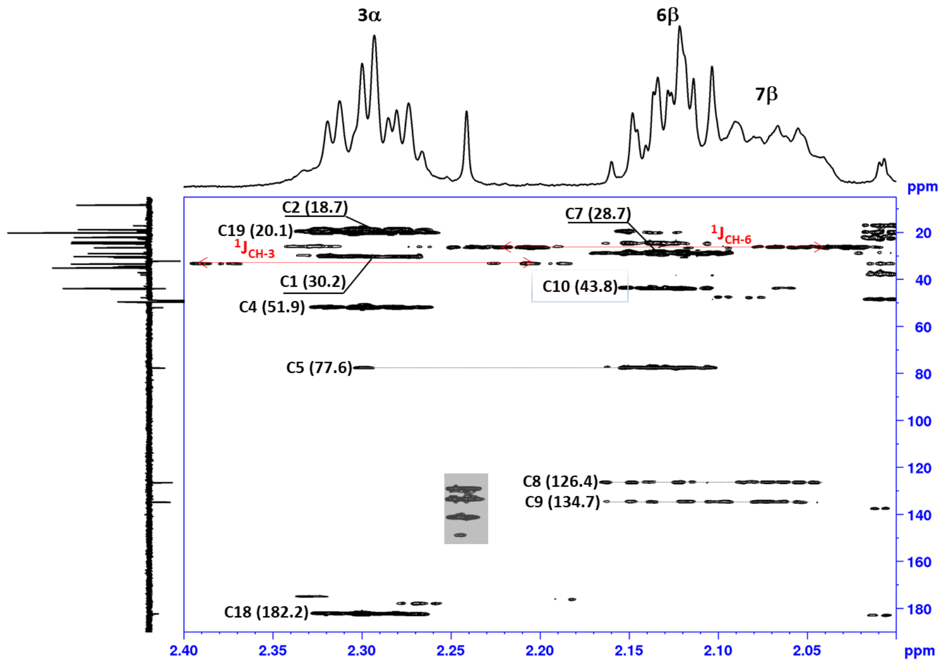

Supplemental Figure 137. Structure elucidation of **7**, ^{1}H-^{13}C HMBC spectrum, detail aliphatic range (δ_H 2.4—2)

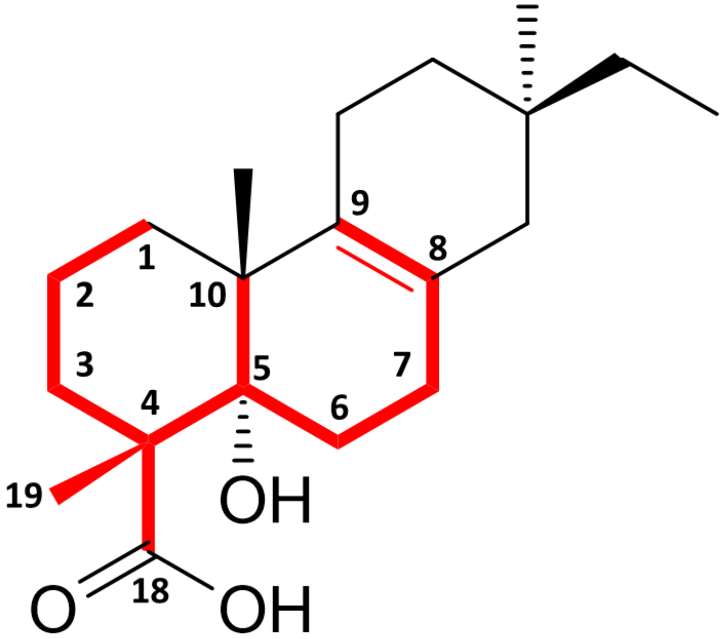

Supplemental Figure 138. Structure elucidation of **7**, structure of **7** with fragments elucidated from HMBC correlations in the aliphatic range (δ_H 2.4—2)

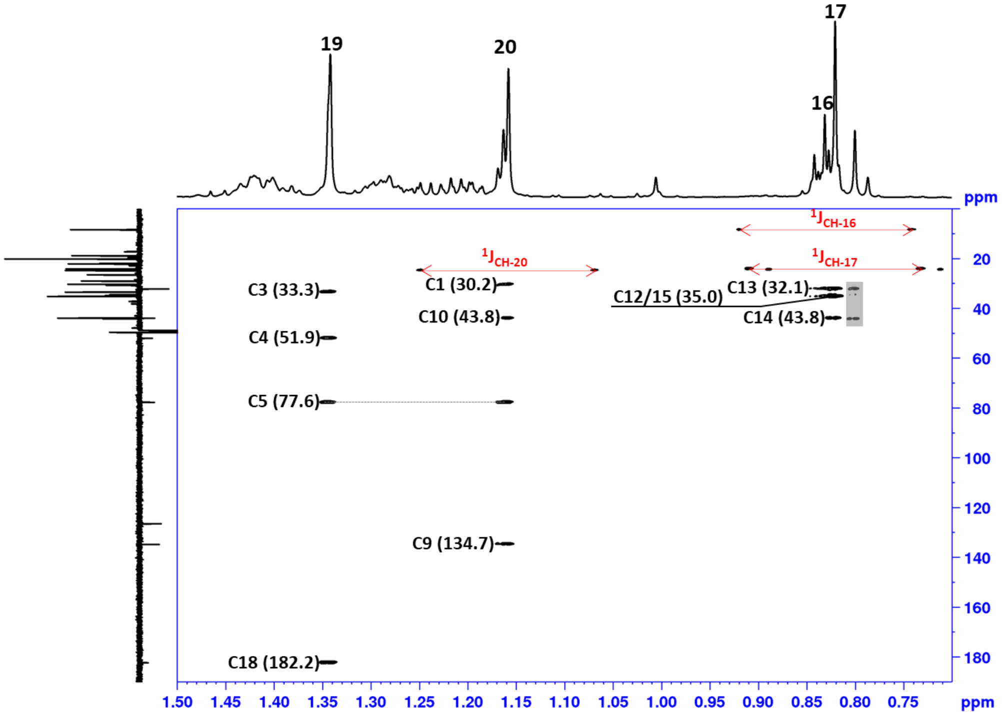

Supplemental Figure 139. Structure elucidation of **7**, ^1H-^{13}C HMBC spectrum, detail aliphatic range (δ_H 1.5—0.7)

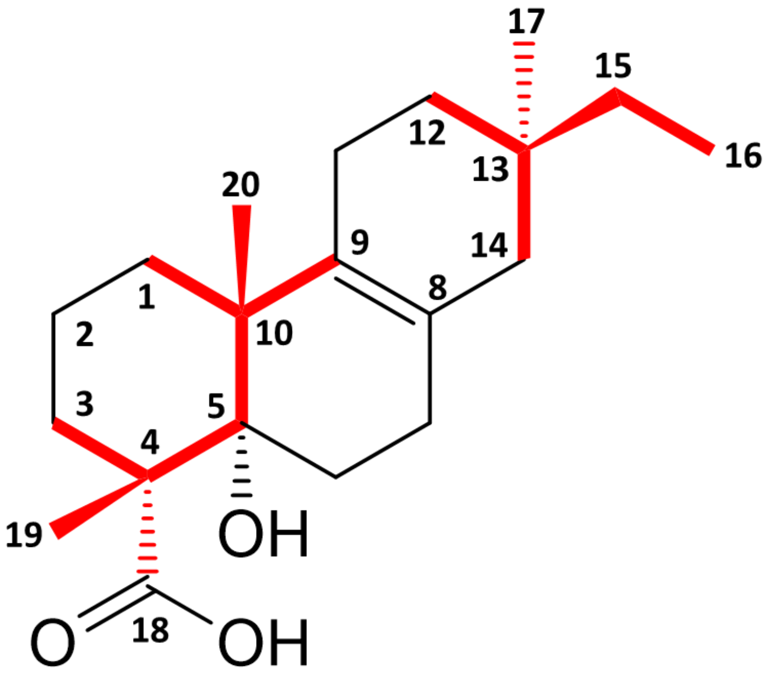

Supplemental Figure 140. Structure elucidation of **7**, structure of **7** with fragments elucidated from HMBC correlations in the aliphatic range (δ_H 1.5—0.7)

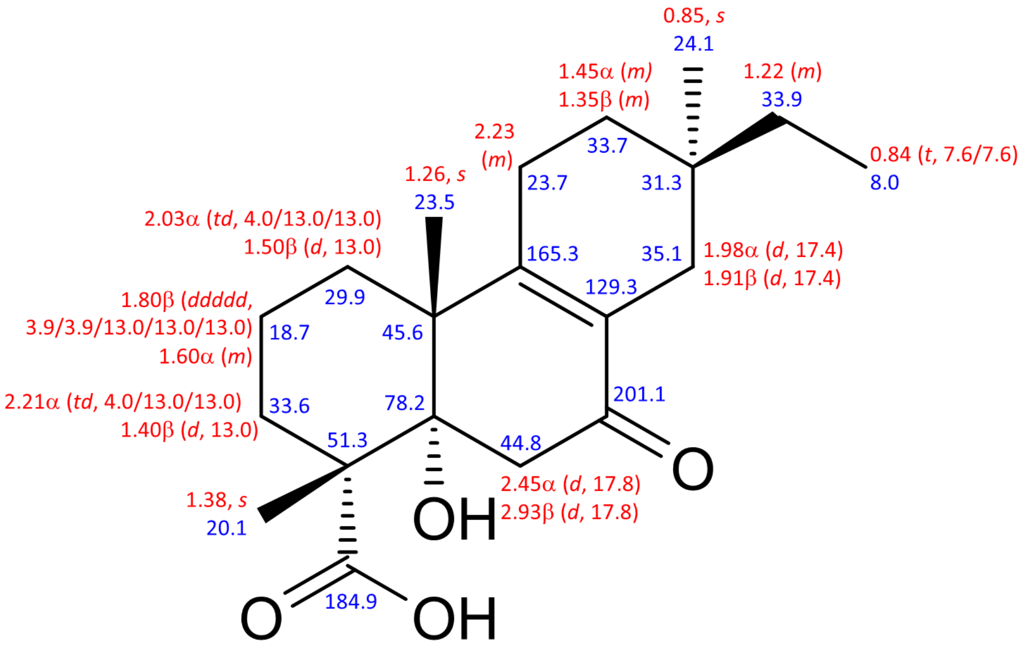

Supplemental Figure 141. Structure elucidation of **8**, overview

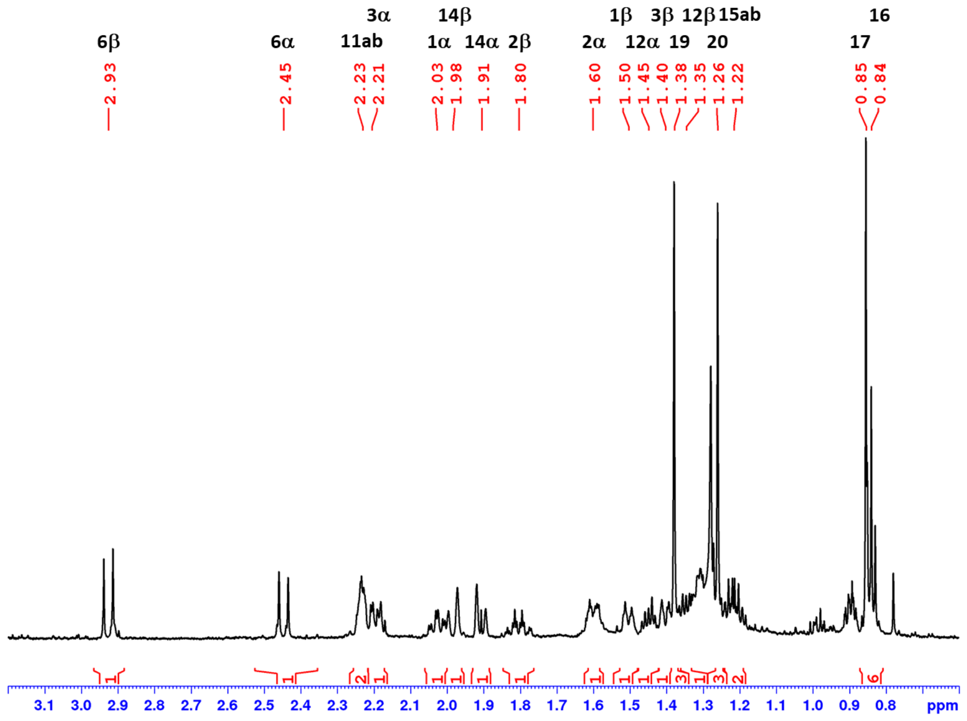

Supplemental Figure 142. Structure elucidation of **8**, ^{1}H-NMR spectrum, full range

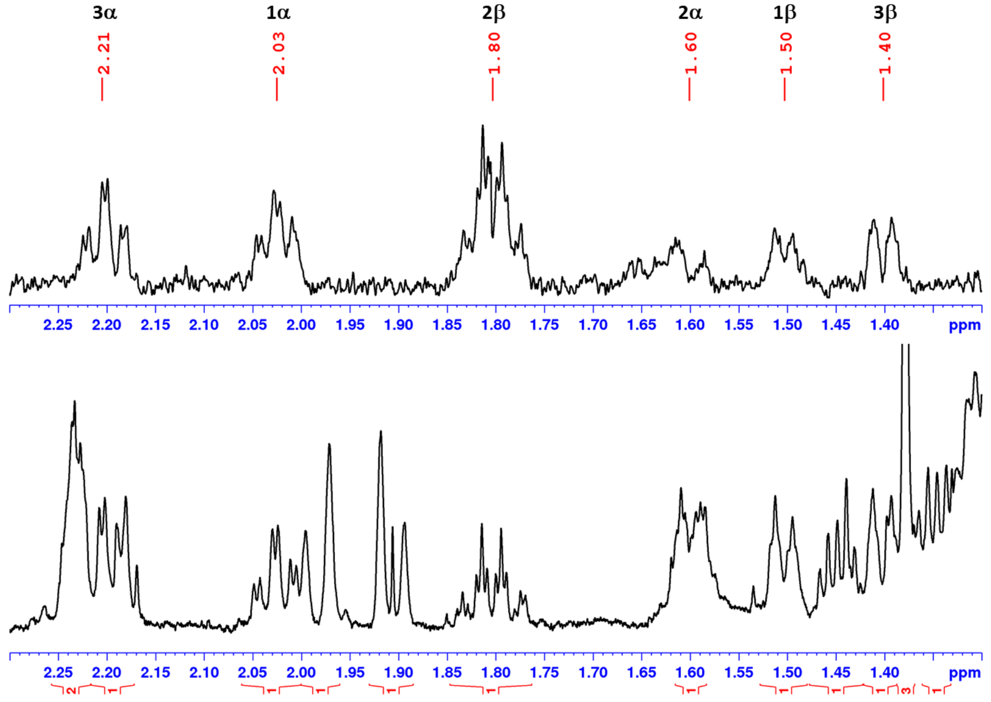

Supplemental Figure 143. Structure elucidation of **8**, comparison ^{1}H-NMR spectrum, detail positions 1 to 3. The upper spectrum is a SELTOCSY with the transmitter set on resonance with H-2β.

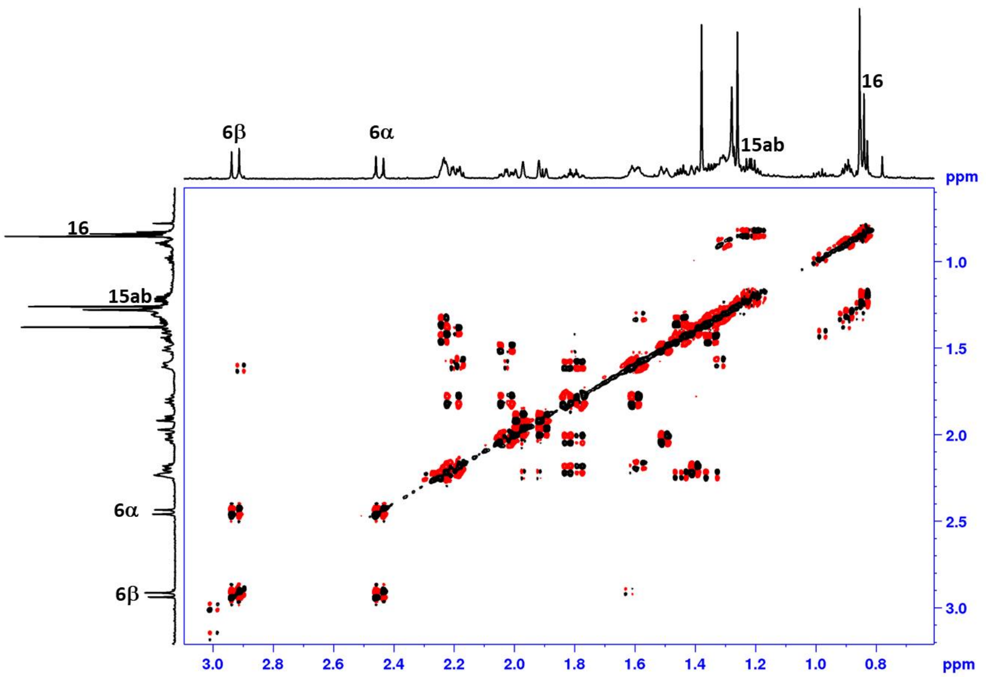

Supplemental Figure 144. Structure elucidation of **8**, ^{1}H-^{1}H COSY spectrum, full range

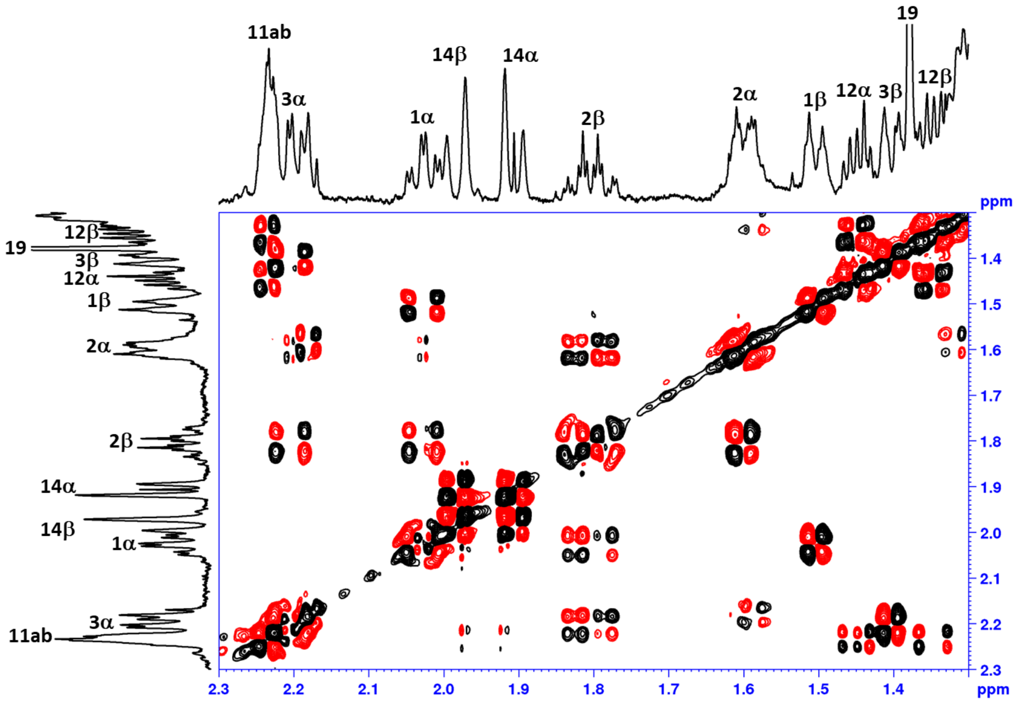

Supplemental Figure 145. Structure elucidation of **8**, ^{1}H-^{1}H COSY spectrum, detail aliphatic range (δ_H 2.3—1.3)

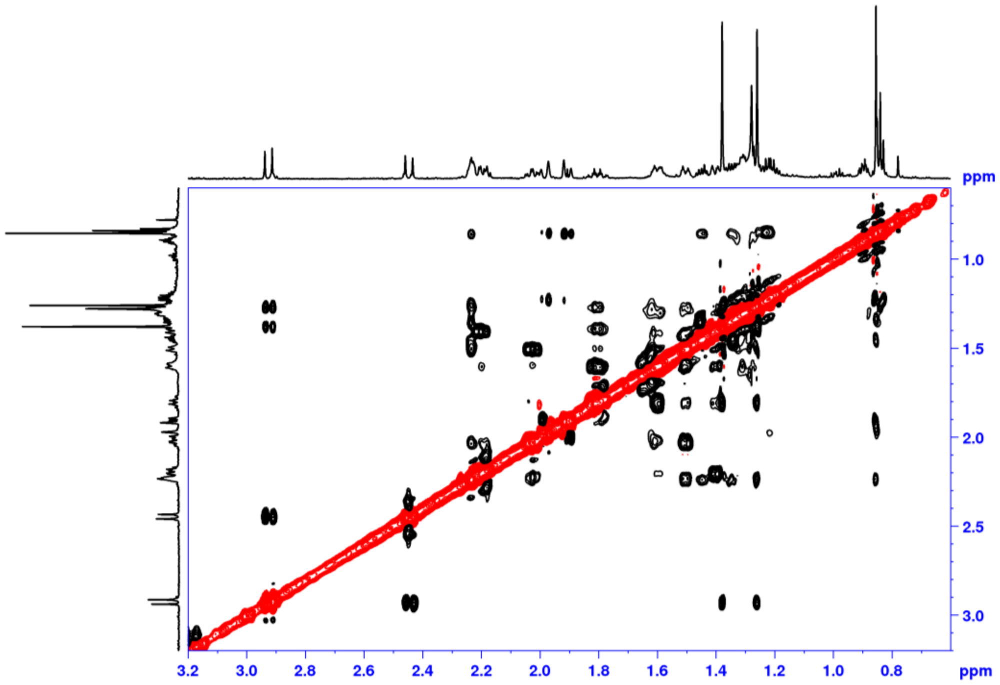

Supplemental Figure 146. Structure elucidation of **8**, ^{1}H-^{1}H ROESY spectrum, full range

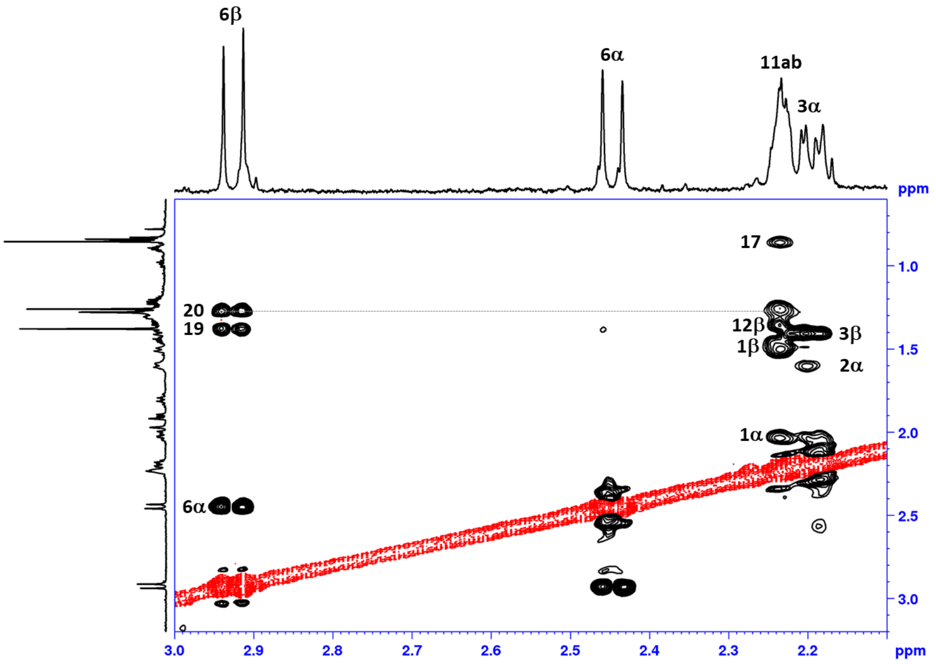

Supplemental Figure 147. Structure elucidation of **8**, ^{1}H-^{1}H ROESY spectrum, detail aliphatic range (δ_H 3.0—2.1)

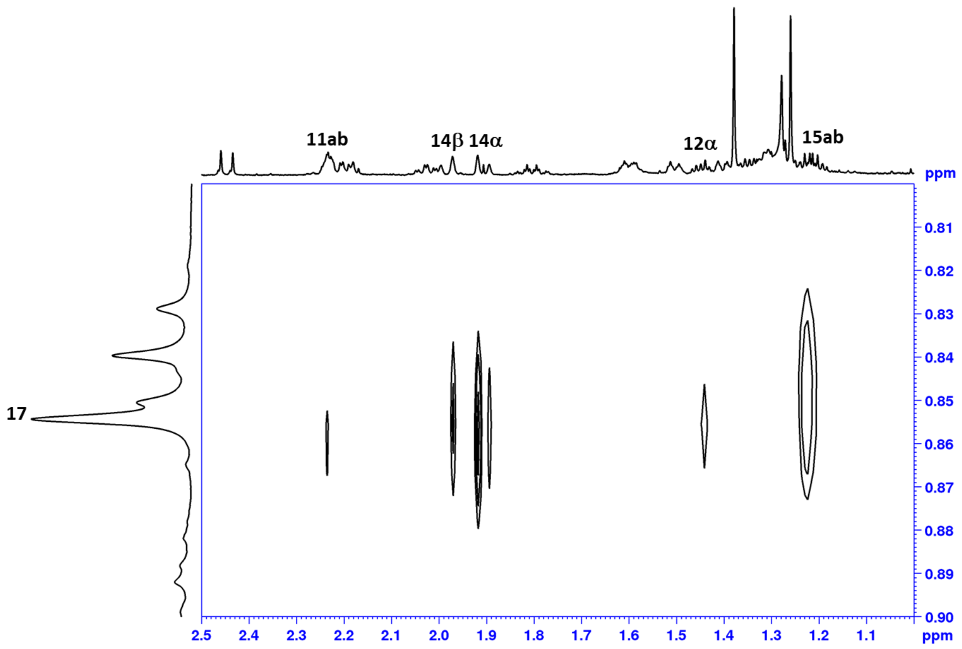

Supplemental Figure 148. Structure elucidation of **8**, ^{1}H-^{1}H ROESY spectrum, correlations from CH_3-17

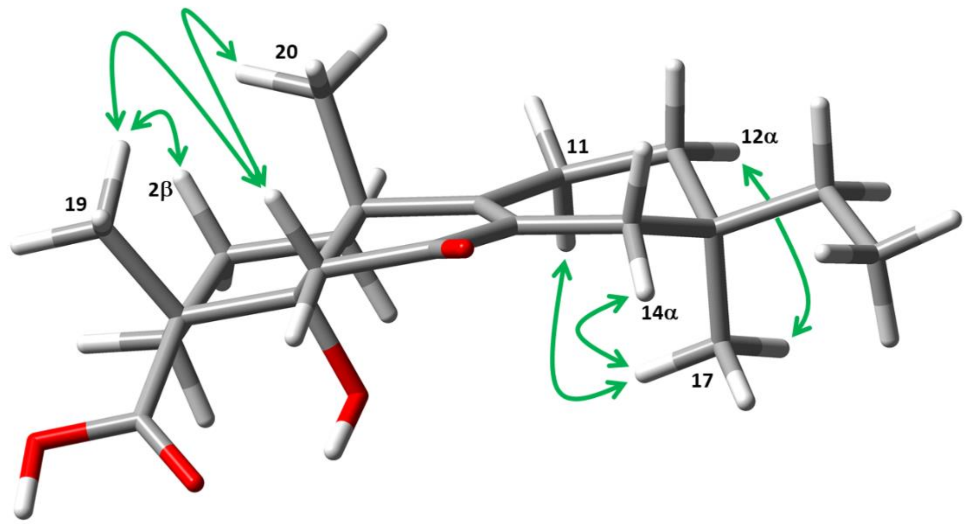

Supplemental Figure 149. Structure elucidation of **8**, structure of **8** with important ROESY correlations

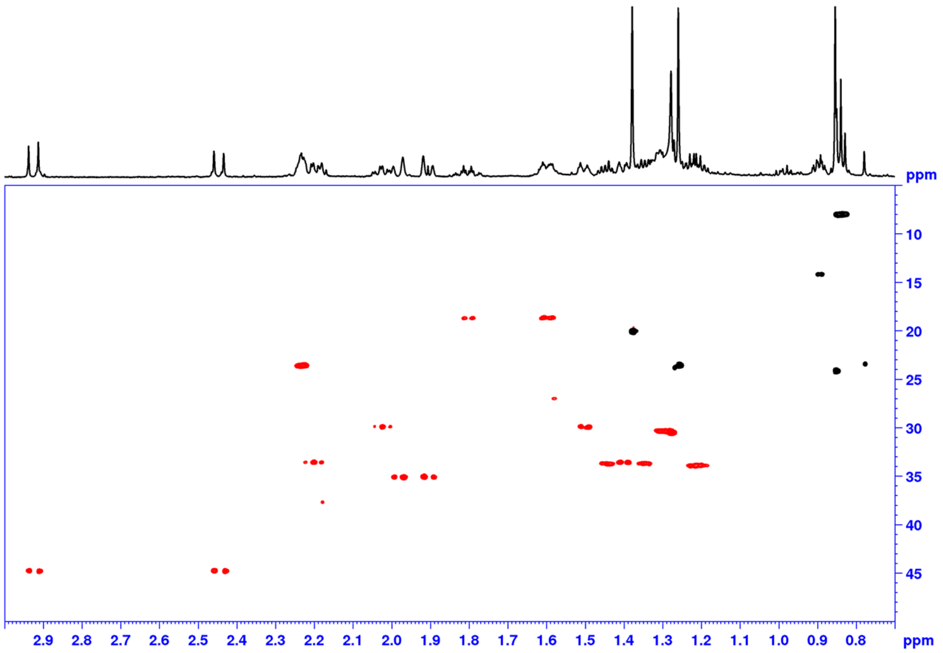

Supplemental Figure 150. Structure elucidation of **8**, ^{1}H-^{13}C HSQC spectrum, full range

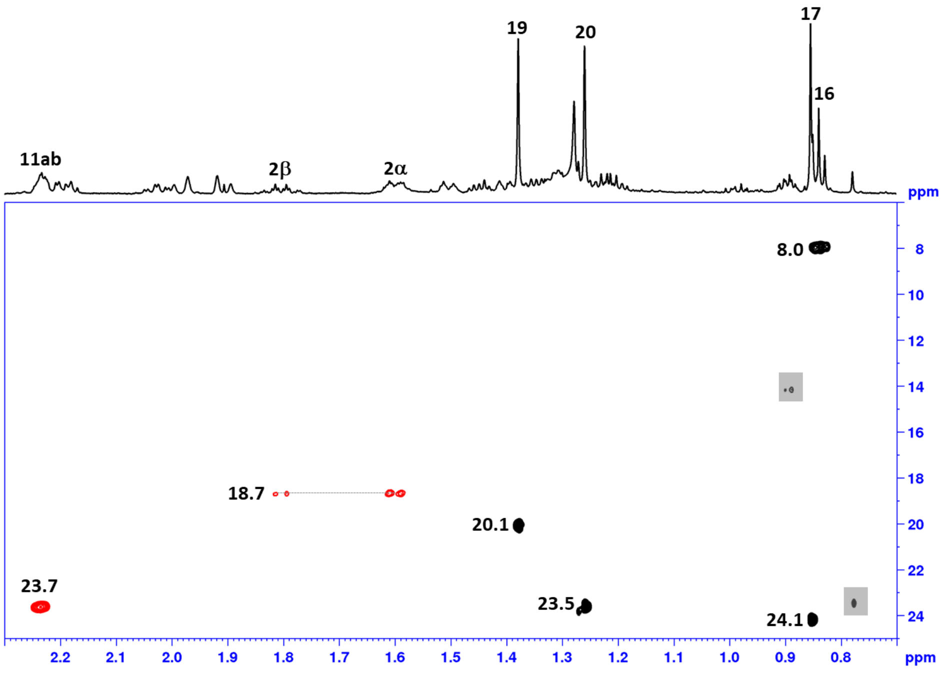

Supplemental Figure 151. Structure elucidation of **8**, ^{1}H-^{13}C HSQC spectrum, detail methyl range

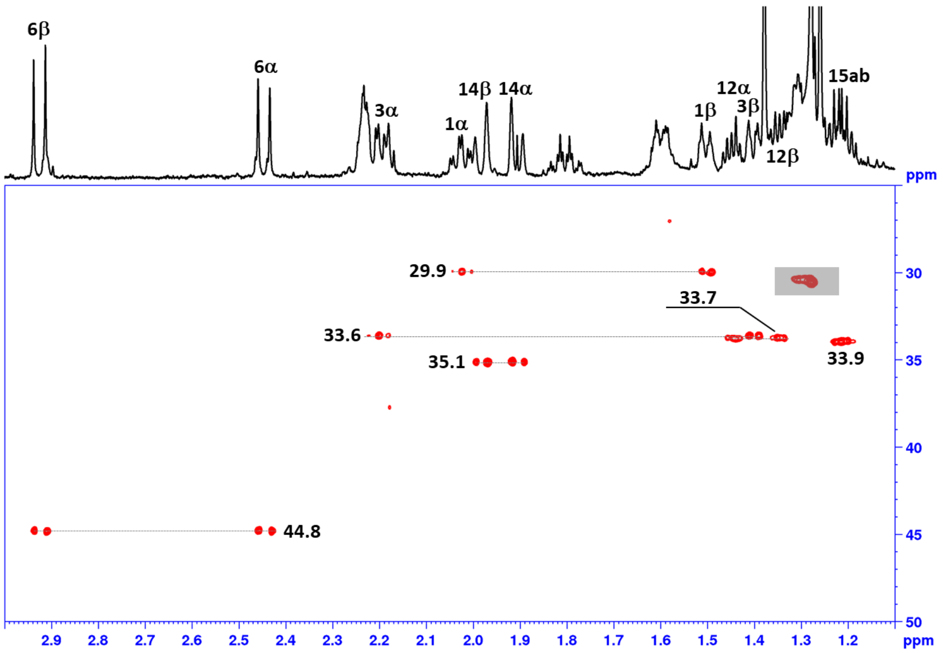

Supplemental Figure 152. Structure elucidation of **8**, ^{1}H-^{13}C HSQC spectrum, detail aliphatic range

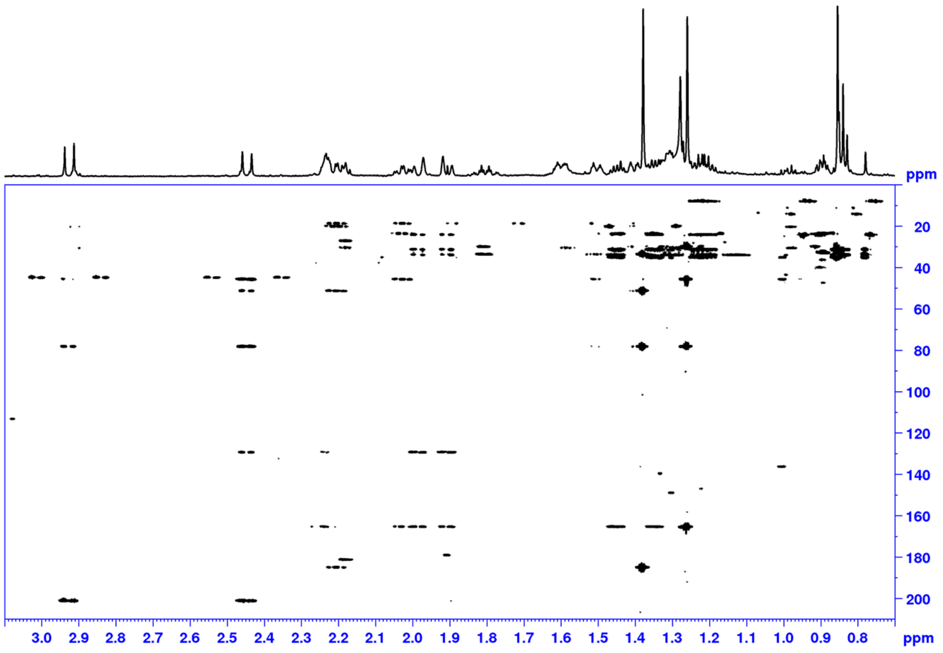

Supplemental Figure 153. Structure elucidation of **8**, ^{1}H-^{13}C HMBC spectrum, full range

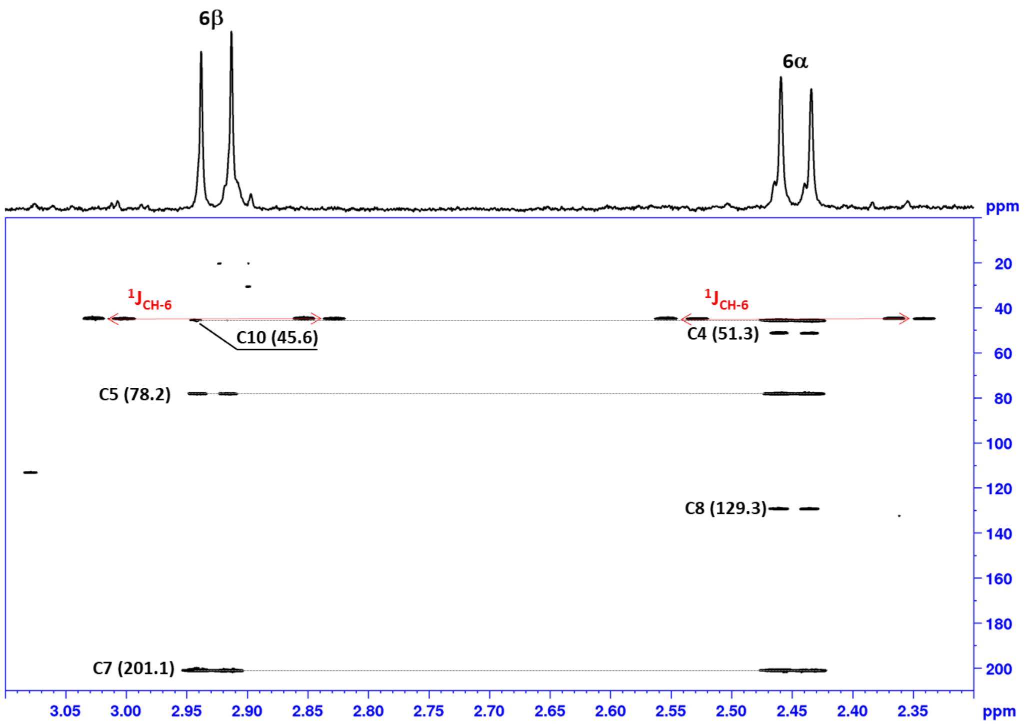

Supplemental Figure 154. Structure elucidation of **8**, ^{1}H-^{13}C HMBC spectrum, detail aliphatic range (δ_H 3.1—2.3)

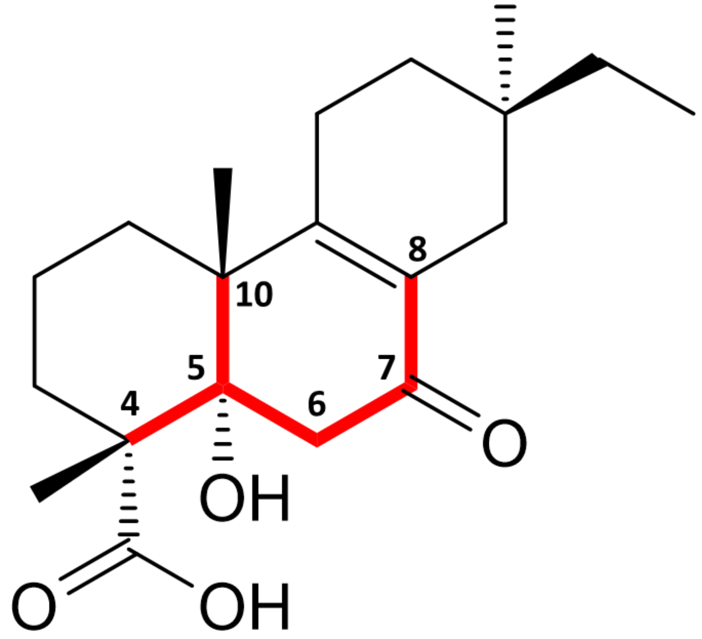

Supplemental Figure 155. Structure elucidation of **8**, structure of **8** with fragments elucidated from HMBC correlations in the aliphatic range (δ_H 3.1—2.3)

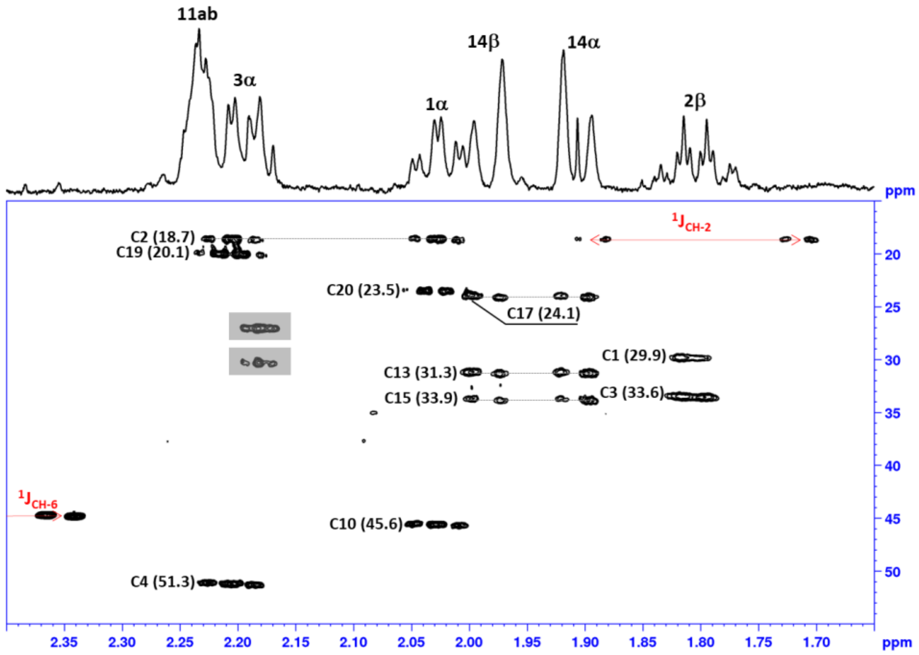

Supplemental Figure 156. Structure elucidation of **8**, ^{1}H-^{13}C HMBC spectrum, detail aliphatic range (δ_H 2.4—1.65), high field F1 range

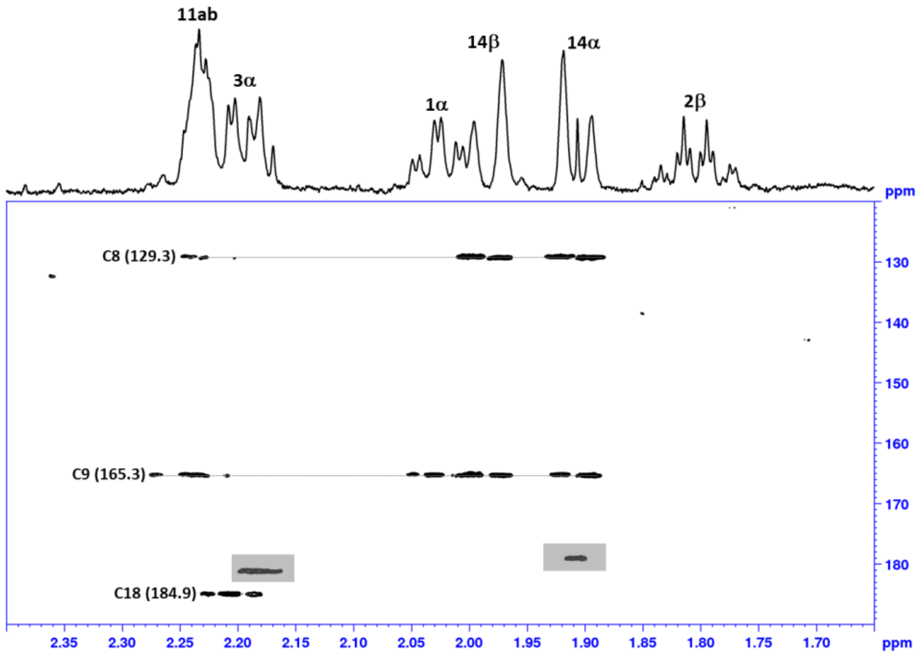

Supplemental Figure 157. Structure elucidation of **8**, ^{1}H-^{13}C HMBC spectrum, detail aliphatic range (δ_H 2.4—1.65), low field F1 range

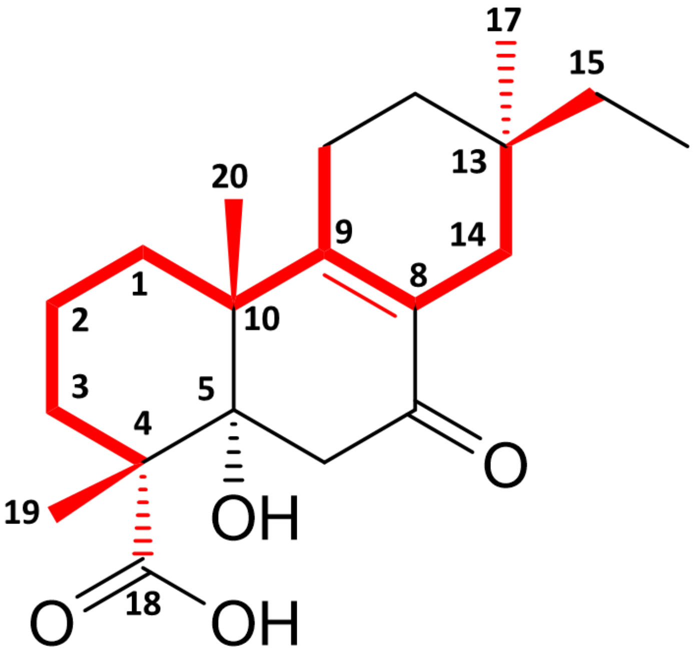

Supplemental Figure 158. Structure elucidation of **8**, structure of **8** with fragments elucidated from HMBC correlations in the aliphatic range (δH 2.4—1.65)

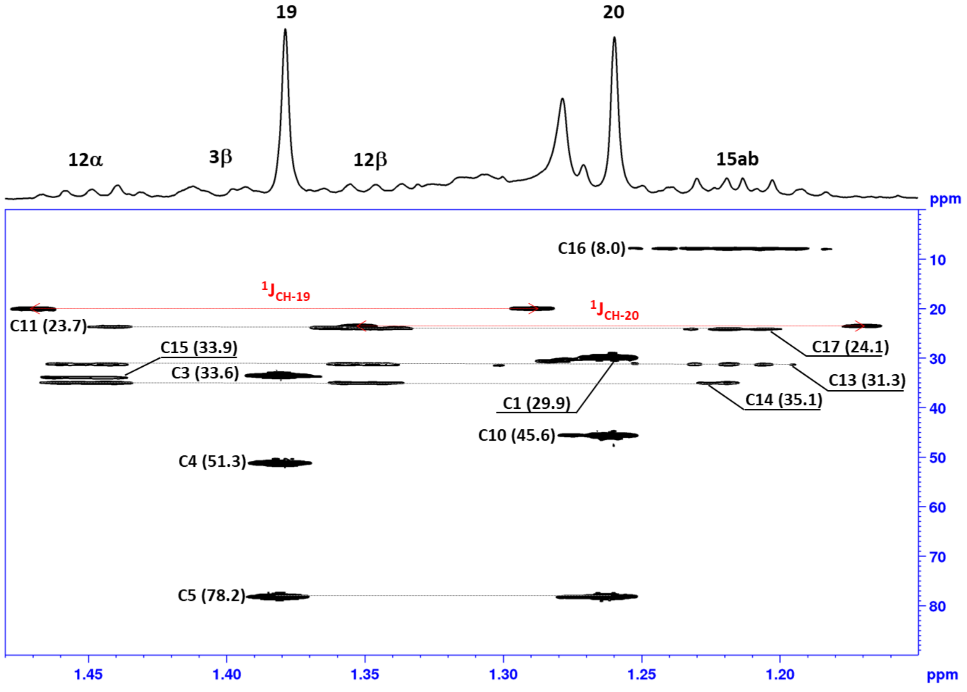

Supplemental Figure 159. Structure elucidation of **8**, ^{1}H-^{13}C HMBC spectrum, detail aliphatic range (δH 1.5—1.15), high field F1 range

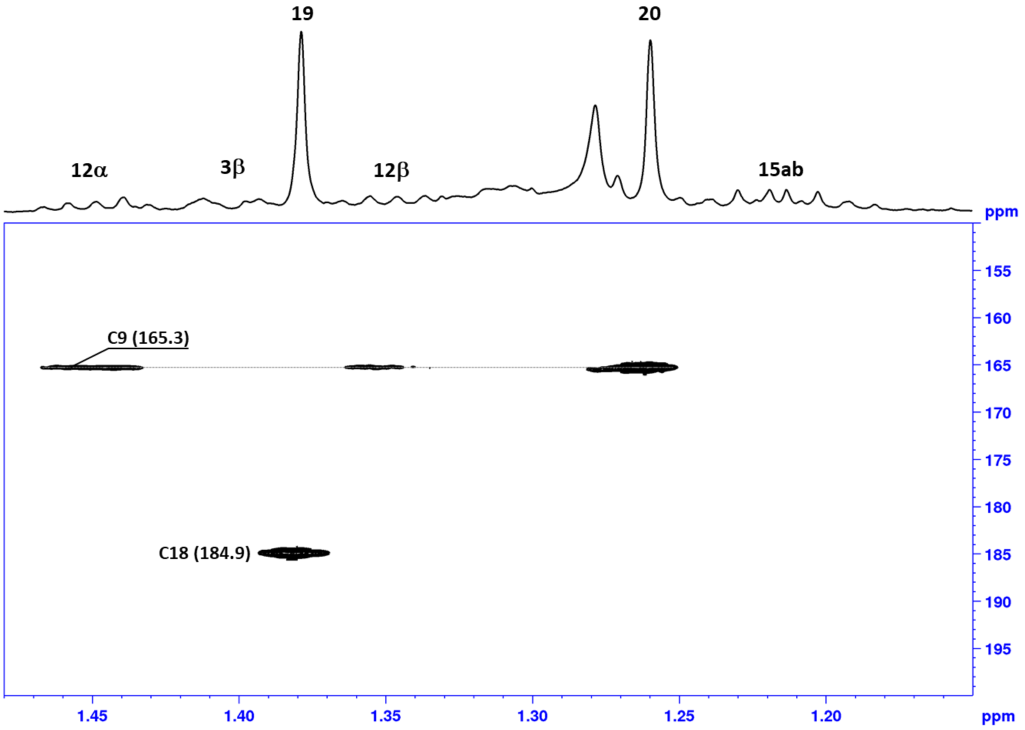

Supplemental Figure 160. Structure elucidation of **8**, ^{1}H-^{13}C HMBC spectrum, detail aliphatic range (δ_H 1.5—1.15), low field F1 range

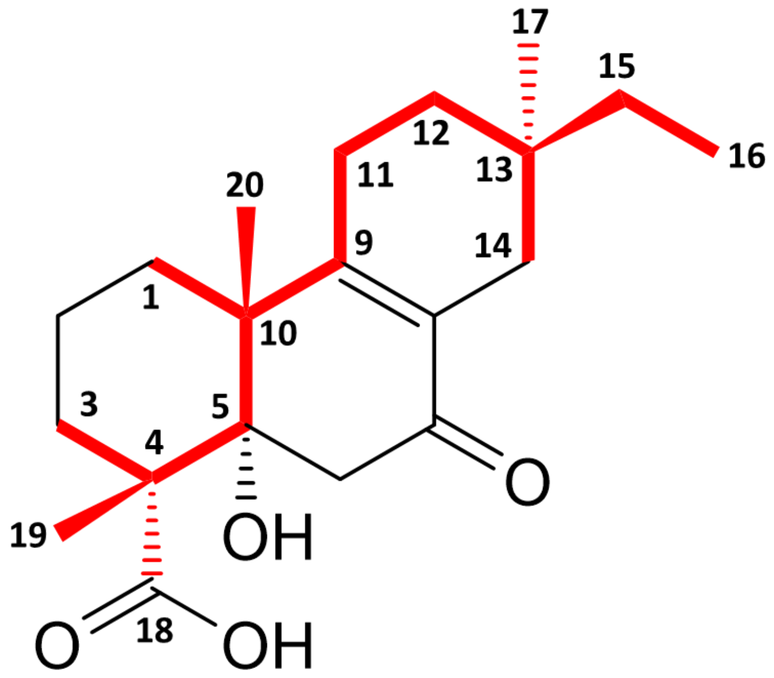

Supplemental Figure 161. Structure elucidation of **8**, structure of **8** with fragments elucidated from HMBC correlations in the aliphatic range (δ_H 1.5—1.15)

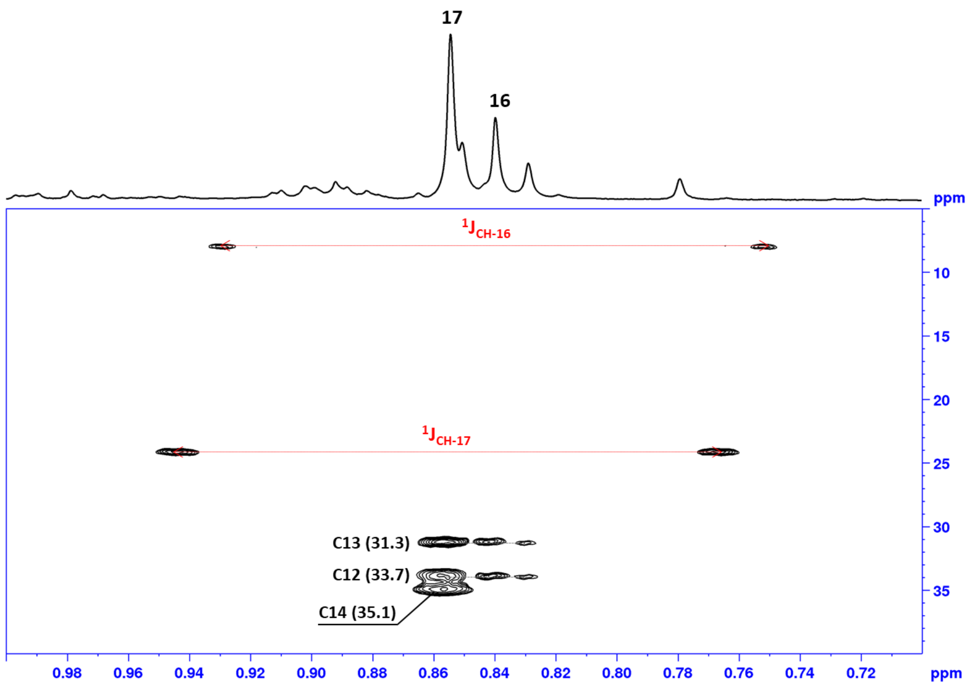

Supplemental Figure 162. Structure elucidation of **8**, ^{1}H-^{13}C HMBC spectrum, detail aliphatic range (δ_H 1.0—0.7), low field F1 range

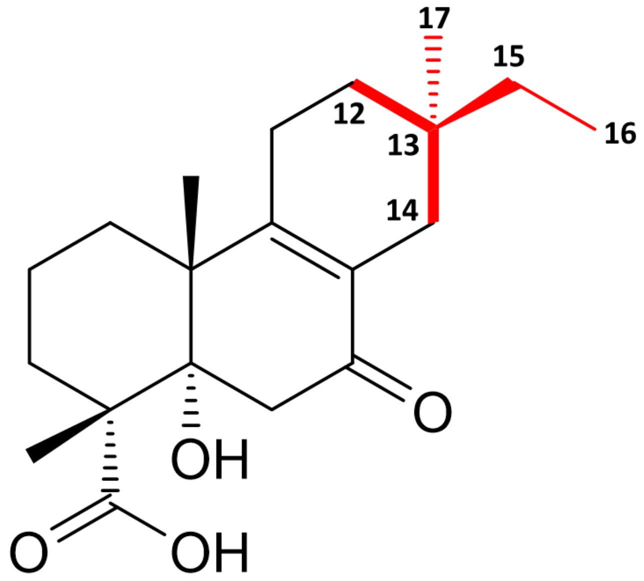

Supplemental Figure 163. Structure elucidation of **8**, structure of **8** with fragments elucidated from HMBC correlations in the aliphatic range (δ_H 1.0—0.7)

Supplemental Table 1. Chemical shifts of positions 1—3 in ring A of compound **2**—**8**

^{13}C chemical shifts

compound	C-1	C-2	C-3	pos. 5
**2**	39.2	19.3	37.6	α-H
**3**	38.2	19.1	37.9	α-H
**4**	37.7	19.0	37.5	α-H
**5**	32.3	19.1	33.6	α-OH
**6**	32.1	18.7	33.0	α-OH
**7**	30.2	18.7	33.3	α-OH
**8**	29.9	18.7	33.6	α-OH

^{1}H chemical shifts

compound	H1α	H1β	H2α	H2β	H3α	H3β	pos. 5
**2**	1.38	2.32	1.68	1.82	1.79	1.65	α-H
**3**	1.59	2.42	1.77	1.89	1.82	1.73	α-H
**4**	1.49	2.24	1.71	1.83	1.83	1.65	α-H
**5**	2.07	1.99	1.71	1.88	2.28	1.50	α-OH
**6**	1.51	1.30	1.53	1.70	2.22	1.39	α-OH
**7**	1.80	1.43	1.58	1.72	2.29	1.41	α-OH
**8**	2.03	1.50	1.60	1.80	2.21	1.40	α-OH

Supplemental Table 2. NMR spectroscopic data (700 MHz, MeOH-d_3) for DHAA derivatives (**2**–**4**).

compound 2 (7β-hydroxy-dehydroabietic acid)			
**position**	**δC [ppm], type**	**δH [ppm], *mult.* (JHH [Hz])**	**HMBC**
**1α**	39.2, CH_2	1.38, *td* (3.0/13.5/13.5)	2, 20, 3, 10, 9
**1β**	39.2, CH_2	2.32, *d* (13.5)	2, 20, 3, 10, 5
**2α**	19.3, CH_2	1.68, *d* (13.5)	1, 3, 10
**2β**	19.3, CH_2	1.82, *ddd* (13.5/13.5/13.5)	1, 3
**3α**	37.6, CH_2	1.79, *td* (3.2/13.5/13.5)	2, 19, 1, 4, 18
**3β**	37.6 CH_2	1.65, *d* (13.5)	
**4**	48.1, C	-	
**5α**	44.8, CH	2.20, *dd* (7.0/7.0)	19, 20, 6, 10, 4, 7
**6α/6β**	33.1, CH_2	1.82, *bs*	10, 5, 7, 8
**7α**	71.1, CH	4.74, *dd* (8.5/8.5)	6, 14, 8, 9
**8**	138.8, C	-	
**9**	147.8, C	-	
**10**	38.3, C	-	
**11**	124.8, CH	7.14, *d* (8.2)	10, 8, 13
**12**	126.2, CH	7.05, *dd* (1.5/8.2)	15, 14, 9
**13**	146.9, C	-	
**14**	126.0, CH	7.35, *d* (1.5)	7, 15, 12, 9
**15**	34.7, CH	2.84, *hept.* (6.9)	16/17, 12, 14, 13
**16**	24.2, CH_3	1.22, *d* (6.9)	17, 15, 13
**17**	24.2, CH_3	1.22, *d* (6.9)	16, 15, 13
**18**	182.7, C	-	
**19**	16.8, CH_3	1.26, *s*	3, 4, 5, 18
**20**	25.5, CH_3	1.27, *s*	1, 10, 5, 9

compound 3 (7-oxo-dehydroabietic acid)			
**position**	**δC [ppm], type**	**δH [ppm], *mult.* (JHH [Hz])**	**HMBC**
**1α**	38.2, CH_2	1.59, *td* (3.1/12.9/12.9)	2, 20, 3, 10, 9
**1β**	38.2, CH_2	2.42, *d* (12.9)	2, 20, 3, 10, 5
**2α**	19.1, CH_2	1.77, *ddddd* (3.1/3.1/3.1/3.1/12.9)	1, 3, 10
**2β**	19.1, CH_2	1.89, *ddddd (*3.1/3.1/12.9/12.9/12.9)	1, 3
**3α**	37.8, CH_2	1.82, *td* (3.1/12.9/12.9)	2, 19, 1, 4, 18
**3β**	37.8, CH_2	1.73, *d* (12.9)	
**4**	47.5, C	-	
**5α**	45.3, CH	2.66, *dd* (3.0/14.1)	19, 20, 6, 10, 4, 9, 18, 7
**6α**	38.6, CH_2	2.38, *dd* (3.0/17.8)	7, 10, 4, 5, 8
**6β**	38.6, CH_2	2.78, *dd* (14.1/17.8)	7, 10, 4, 5
**7**	200.8, C	-	

**8**	131.4, C	-	
**9**	154.9, C	-	
**10**	38.4, C	-	
**11**	124.9, CH	7.39, *d* (8.2)	10, 8, 13,7
**12**	133.8, CH	7.48, *dd* (2.1/8.2)	15, 14, 9
**13**	147.8, C	-	
**14**	125.3, CH	7.80, *d* (2.1)	7, 15, 12, 9
**15**	34.6, CH	2.92, *hept.* (6.7)	16/17, 12, 14, 13
**16**	23.9, CH_3	1.24, *d* (6.7)	17, 15, 13
**17**	23.9, CH_3	1.24, *d* (6.7)	16, 15, 13
**18**	181.8, C	-	
**19**	16.7, CH_3	1.33, *s*	3, 4, 5, 18
**20**	23.5, CH_3	1.28, *s*	1, 10, 5, 9

**compound 4 (11,12-dihydroxy-7-oxoabieta-8,13-dien-18-oic acid)**			
**position**	**δ_C [ppm], type**	**δ_H [ppm], *mult*. (J_{HH} [Hz])**	**HMBC**
**1α**	37.7, CH_2	1.49, *m*	
**1β**	37.7, CH_2	2.24, *m*	
**2α**	19.0, CH_2	1.71, *m*	
**2β**	19.0, CH_2	1.83, *m*	
**3α**	37.5, CH_2	1.83, *m*	
**3β**	37.5, CH_2	1.65, *m*	
**4**	n.d., C	-	
**5α**	45.7, CH	2.52, *m*	
**6α**	37.8, CH_2	2.21, *m*	
**6β**	37.8, CH_2	2.56, *m*	
**7**	n.d., C	-	
**8**	n.d., C	-	
**9**	162.1, C	-	
**10**	n.d., C	-	
**11**	69.0, CH	4.38, *m*	
**12**	72.6, CH	4.24, *m*	
**13**	149.1, C	-	
**14**	111.9, CH	6.22, s	9, 12, 15
**15**	30.0, CH	2.72, *m*	13, 14, 12, 16, 17
**16**	22.7, CH_3	1.07, *d* (6.7)	
**17**	20.4, CH_3	1.13, *d* (6.7)	
**18**	n.d., C	-	
**19**	n.d., CH_3	n.d.	
**20**	18.7, CH_3	1.35, *s*	1, 10, 5, 9
